# CEMP1 Induces Transformation in Human Gingival Fibroblasts

**DOI:** 10.1371/journal.pone.0127286

**Published:** 2015-05-26

**Authors:** Mercedes Bermúdez, Ivan Imaz-Rosshandler, Claudia Rangel-Escareño, Margarita Zeichner-David, Higinio Arzate, Gabriela E. Mercado-Celis

**Affiliations:** 1 Laboratorio de Biología Periodontal, Facultad de Odontología, Universidad Nacional Autónoma de México, Mexico City, México; 2 Instituto Nacional de Medicina Genomica, SSA, Mexico City, México; 3 Ostrow School of Dentistry, University of Southern California, Los Angeles, California, United States of America; Boston University Goldman School of Dental Medicine, UNITED STATES

## Abstract

Cementum Protein 1 (CEMP1) is a key regulator of cementogenesis. CEMP1 promotes cell attachment, differentiation, deposition rate, composition, and morphology of hydroxyapatite crystals formed by human cementoblastic cells. Its expression is restricted to cementoblasts and progenitor cell subpopulations present in the periodontal ligament. CEMP1 transfection into non-osteogenic cells such as adult human gingival fibroblasts results in differentiation of these cells into a “mineralizing” cell phenotype. Other studies have shown evidence that CEMP1 could have a therapeutic potential for the treatment of bone defects and regeneration of other mineralized tissues. To better understand CEMP1’s biological effects in vitro we investigated the consequences of its expression in human gingival fibroblasts (HGF) growing in non-mineralizing media by comparing gene expression profiles. We identified several mRNAs whose expression is modified by CEMP1 induction in HGF cells. Enrichment analysis showed that several of these newly expressed genes are involved in oncogenesis. Our results suggest that CEMP1 causes the transformation of HGF and NIH3T3 cells. CEMP1 is overexpressed in cancer cell lines. We also determined that the region spanning the CEMP1 locus is commonly amplified in a variety of cancers, and finally we found significant overexpression of CEMP1 in leukemia, cervix, breast, prostate and lung cancer. Our findings suggest that CEMP1 exerts modulation of a number of cellular genes, cellular development, cellular growth, cell death, and cell cycle, and molecules associated with cancer.

## Introduction

Cementum extracellular matrix contains specific molecules expressed by cementoblasts and their progenitor cells present in the periodontal ligament. Amongst these unique molecules, Cementum Attachment Protein (PTPLa/CAP) and Cementum Protein 1 (CEMP1) are believed to regulate the biological activities of periodontal ligament cells [1–6]. The presence of these cementum-specific markers, their structural characterization and their patterns of gene expression has brought a better understanding of the molecular mechanisms that control biomineralization during cementum and bone formation [1–7]. In vitro studies using human cementoblasts have shown that CEMP1 is a key regulator of the biomineralization process; it promotes cell attachment and differentiation, regulates the deposition rate, composition and morphology of hydroxyapatite crystals [8]. CEMP1 expression is restricted to cementoblasts, and progenitor cells subpopulations present in the human periodontium [9]. Recent studies have shown that CEMP1 transfection into non-mineralizing cells like adult human gingival fibroblasts (HGF/CEMP1) resulted in the transdifferentiation of these cells toward a mineralizing cell phenotype [10]. Application of these properties towards translational studies have provided evidence that human recombinant CEMP1 protein (hrCEMP1) promotes bone regeneration in critical-size calvarial defects in rats suggesting a therapeutic potential of this protein for the treatment of bone defects as well as regeneration of mineralized tissues [11].

All previous in-vitro studies using CEMP1 were carried under conditions favoring the induction of mineralized phenotypes, therefore to further understand the biological properties of CEMP1, we need to determine the effects of inducing non-mineralizing cells like HGF cells grown in non-mineralizing conditions. In this study, we report the results of the analysis of gene expression of HGF/CEMP1 cells using microarrays. Several mRNAs whose expression is modified by CEMP1 overexpression in these cells were identified and several of these genes are involved in cancer. Besides, soft agar assays showed that CEMP1 causes the transformation of HGF and NIH3T3 cells. Furthermore, we found that CEMP1 is over expressed in several cancer cell lines and it was determined that the chromosomal region spanning the CEMP1 locus is commonly amplified in a variety of cancers, like leukemia, cervix, breast, prostate and lung cancer. Our results suggest that CEMP1 functions in the modulation of a number of cellular genes like those involved in development, growth, cell death, cell cycle and molecules associated with cancer.

## Materials and Methods

### Ethics Statement

The use of human tissue from the oral cavity for the generation and culturing of human fibroblasts was reviewed and approved by the Ethics Committee at the National University of Mexico School of Dentistry (UNAM). Tissue samples were obtained from the donors that underwent routine oral surgery procedures.

### Cell culture

Human gingival fibroblasts (HGF) were isolated and grown as previously describe [12]. NIH-3T3 fibroblasts were purchased from ATCC (CRL-1658). Cells between the 2^nd^ and 5^th^ passage were used for the experimental. The cells were grown in DMEM media supplemented with 10% FBS in a 5% CO2 and 95% air atmosphere in a 100% humidity.

### Construction of pcDNA40-CEMP1 expressing vector and transfection into human gingival fibroblast cells and NIH-3T3 Fibroblasts

The coding region of CEMP1, (GenBank Accession No. NM_001048212) was subcloned into the pENTR/SD/D vector (Invitrogen, Carlsbad, CA). The resultant pENTR/SD/D-CEMP1 cDNA construct was ligated into a pcDNA40 (+) vector [CEMP1-pcDNA40 (+)] (Invitrogen, Carlsbad, CA). The plasmid, pcDNA40-CEMP1, was transfected into HGF (HGF/CEMP1) and NIH-3T3 (NIH-3T3/CEMP1) cells using Lipofectamine 2000 (Invitrogen, Carlsbad, CA). Controls HGF and NIH 3T3 cells were transfected with pcDNA40 empty vector. Stably expressing cells were selected with G418 600 μg/mL, Sigma Chemical Co., St. Louis, MO) up to eight weeks.

### RNA Isolation

HGF/CEMP1, NIH-3T3/ CEMP1, HGF and NIH-3T3 cells were cultured in DMEM media supplemented with 10% FBS. RNA was isolated using RNeasy Mini Kit according to the manufacturer´s directions (Qiagen, Valencia, CA, USA). RNA was quantified spectrometrically (Nanodrop, Thermo Fisher Scientific, Wilmington, DE, USA). For microarrays the integrity of HGF/CEMP1 and HGF RNA was assessed by capillary gel electrophoresis using RNA 6000 Nano Chip (Agilent 2100 Bioanalyzer, Agilent Technologies). Samples with RNA integrity numbers ≥ 7.0 were used for these studies [13].

### Microarray Hybridization, Detection and Preprocessing

HGF/CEMP1 and HGF cells were harvested after 3, 7 and 14 days in culture. Three microarrays/time point, for a total of 18 arrays were used. All protocols were conducted as described in the Affymetrix GeneChip Expression Analysis Technical Manual (Affymetrix Inc., Santa Clara, CA, USA). Briefly, 200 ng of total RNA was converted to first-strand cDNA using Superscript II reverse transcriptase primed with a poly (T) oligomer that incorporated the T7 promoter. Second strand cDNA synthesis was followed by in vitro transcription to generate cRNA. The cRNA products were used as templates for a second cycle cDNA synthesis in order to incorporate dUTPs to the new strand. The cDNA was fragmented using uracil-DNA glycosylase and purin-pirymidin endonuclease. Fragments (40–70mers) were labeled by biotin-labeled deoxynucleotide terminal addition. The labeled cDNA was denatured and hybridized to the Human Gene 1.0 ST microarray (Affymetrix Inc., Santa Clara, CA, USA) for 17 ± 1 hr at 45°C. The microarrays were washed with low (6X SSPE) and high (100mM MES, 0.1M NaCl) stringency solutions and stained with a streptavidin-phycoerythrin conjugate. Fluorescence was amplified by adding biotinylated anti-streptavidin and an additional aliquot of streptavidin-phycoerythrin stain. A confocal scanner (Affymetrix GeneChip Scanner 3000 7G plus) was used to collect the fluorescence signal at 3μm resolution after excitation at 570 nm. The average signal from two sequential scans was calculated for each microarray.

### Microarray Data Analysis

Microarray data analysis was performed using R and Bioconductor software (http://www.bioconductor.org). The Microarray data obtained from this study has been deposited in Gene Expression Omnibus (GEO) under Accession No. GSE53929 provided by NCBI, (NIH, Bethesda, MD.). All samples were normalized with Robust Multiarray Average (RMA) [14], which includes background correction, normalization and calculation of expression values. After pre-processing, a principal components analysis (PCA) was performed as an unsupervised method in order to determine if different phenotypic groups were identifiable at different time points.

Times series analysis was carried out using the Bioconductor time course package [15]. Genes were ranked according to the Hotelling T2 test [16], in order to find genes with expression patterns across time changing between HGF/CEMP1 and normal HGF.

Differential expression analysis between HGF and HGF/CEMP1 cells was performed using limma [17]. Differentially expressed genes between HGF and HGF/CEMP1 were selected based on a fold- change of 2 in absolute value. Benjamini and Hochberg false discovery rate [18] was applied for multiple hypotheses testing, the genes with an adjusted p-value < 0.001 were accepted. Enrichment analysis with IPA (Ingenuity Systems, www.ingenuity.com) and DAVID (Database for Annotation, Visualization, and Integrated Discovery) was performed on each list of selected genes [19–21].

### Quantitative Real-Time PCR

Validation of the microarray analysis was done using quantitative real-time PCR (qRT-PCR). First strand cDNA was synthesized from total RNA (1 μg) using High Capacity cDNA Archive kit according to manufacturer instructions (Applied Biosystems de México, México). Taqman gene expression assays were purchased from Applied Biosystems (Applied Biosystems de México, México). qRT-PCR reactions were carried out at 10 μl total volume and measured with ABI *ViiA*·*7* Sequence Detection *System* (Applied Biosystems de México, México) in 384-well plates. *18S ribosomal* RNA (rRNA, Hs99999901_s1) gene was used as a control of RNA integrity. Samples were run in triplicate, absolute quantification (AQ) was performed using the standard curve method and the average result was reported. The genes included for validation were, CDH1 (Hs01023894_m1), CDH2 (Hs00983061_m1), FGFR2 (Hs01552926_m1), HBEGF (Hs00181813_m1), HMGB2 (Hs01127828_g1) and HOXA5 (Hs00430330_m1). In order to verify the expression of CEMP1 (Hs04185363_s1) in cancer-derived cell lines such as RH28, SMS, PC3, T47D, RD, MCF7, HS578T, MDAMB28, HEPG2, 22RV1, A673, HELA were screened. These lines represent alveolar rhabdomyosarcoma, embryonal rhabdomyosarcoma, prostate cancer, Ewing sarcoma, breast carcinoma and cervical epithelial carcinoma.

### Western blot

To confirm the overexpression of CEMP1 at the protein level, total proteins were isolated from HGF/CEMP1, NIH-3T3/CEMP1, HGF and NIH-3T3 and western blots were performed using anti-human rhCEMP1 polyclonal antibodies [10]. Validation of selected genes was done using, anti-E-Cadherin, anti-N-Cadherin, anti-fibroblast growth factor receptor (FGFR-2), anti-high mobility group (HMG) proteins (HMG-1/2/3 (FL-215), anti-heparin binding epidermal-like growth factor (HB-EGF), anti-HoxA5 and anti-c-Met oncogene were used (Santa Cruz Biotech, CA, USA). Total proteins were isolated from cell layer cultures and all the antibodies were produced against human proteins.

### In silico Analysis of CEMP1 expression in human cancer

To determine the expression of CEMP1 in human tumor samples and their normal tissue counterparts, we used publicly available microarray data from several databases, GEO (http://www.ncbi.nlm.nih.gov/geo/), ArrayExpress (http://www.ebi.ac.uk/arrayexpress/), TBROWSER (http://tagc.univ-mrs.fr/tbrowser/), GENEVESTIGATOR (https://www.genevestigator.com/gv/), RAD (http://www.cbil.upenn.edu/RAD), SMD ((http://smd.princeton.edu/), PEPR (http://microarray.cnmcresearch.org/pgadatatable.asp), YMD (Yale Microarray Database http://medicine.yale.edu/keck/ymd/index.aspx), DRAGON Database (http://pevsnerlab.kennedykrieger.org/dragon.htm), AMAD (Another MicroArray Database. http://www.microarrays.org/AMADFaq.html), SOURCE Search (http://smd.princeton.edu/cgi-bin/source/sourceSearch). The datasets were filtered using >1.5 ó <-1.5 Fold Change cutoff. The Catalogue of Somatic Mutations in Cancer (COSMIC) (http://www.sanger.ac.uk/cosmic) was reviewed in order to determine if CEMP1 somatic mutations are implicated in human cancer.

### Design and Synthesis of CEMP1 siRNA and Vector Construction

siRNA sequences for CEMP1 (NM_001048212.3; GI:313677962) were designed using Invitrogen’s RNAi Designer. The synthesized complementary DNA oligos (Invitrogen, Carlsbad, CA, USA), were annealed to generate a double-stranded oligo and cloned into the linearized pcDNA 6.2-GW/EmGFP-miR vector (Invitrogen, Carlsbad, CA, USA), using T4 DNA ligase. The Neg-miRNA control plasmid was included in the Block-iT-Pol II miR RNAi Expression Vector Kit. All of the vectors were transformed into One Shot TOP10 Chemically Competent E. coli (Invitrogen, Carlsbad, USA), and the colonies containing spectinomycin-resistant transformants were further analyzed for the desired expression. The recombinant vectors were purified with a purification kit (Invitrogen, Carlsbad, USA) and confirmed by sequencing.

### Cell Culture and Stable Transfection of HGF/CEMP1 with miRNA

The vectors with CEMP1-miRNA, CEMP1-miRNA2 or the Neg-miRNA were transfected into HGF/CEMP1 using Lipofectamine RNAiMAX Transfection Reagent (Invitrogen, Carlsbad, USA) according to the manufacturer’s protocol. The cells were transiently transfected with each miRNA during 48 hours, when the percentage of fluorescent cells was more than 80%. For effective screening of RNAi sequences targeting CEMP1 quantitative real-time polymerase chain reaction (PCR) and western blotting were used. For further study, transfectants were selected by fresh DMEM medium containing 12.5-μg/mL blasticidin (Invitrogen Corp.) every 3 to 4 days up to 8 weeks until blasticidin-resistant colonies could be identified.

### Soft-agar colony formation assay

In vitro tumorigenicity was determined on the basis of cell growth in a soft agar colony assay. Soft agar colony-forming assay was performed in triplicate of at least three independent experiments. HGF, HGF/CEMP1, siRNA-HGF/CEMP1, NIH3T3 and NIH3T3/CEMP1 cells were analyzed by colony-forming assay. Cells (1x10^4^cells/well) were plated in 6-well plates in DMEM medium containing 0.35% low melting point agar (LMP) coated with a 0.7% LMP agar layer. The cells were cultured at 37°C in 5% CO^2^ for 30 days. Every 7 days 500 μl of fresh medium was added to each well and visible colonies were photographed and counted using Open CFU 3.3 as previously described [22].

### Statistical analysis

Results are presented as mean ± SD. Statistical analysis was carried out with Student’s t-test to compare HGF vs HGF/CEMP1 and NIH-3T3 vs NIH-3T3/CEMP1 expression as well as NIH-3T3 vs NIH-3T3/CEMP1 colony formation. Expression and group means were compared by ANOVA, followed by Dunnet test against control if the former was significant. Pearson´s test was carried out to correlate the expression at RNA and protein level. A p value of less than 0.05 was considered statistically significant.

## Results

### Expression of CEMP1 in HGF cells

**Fig 1:**
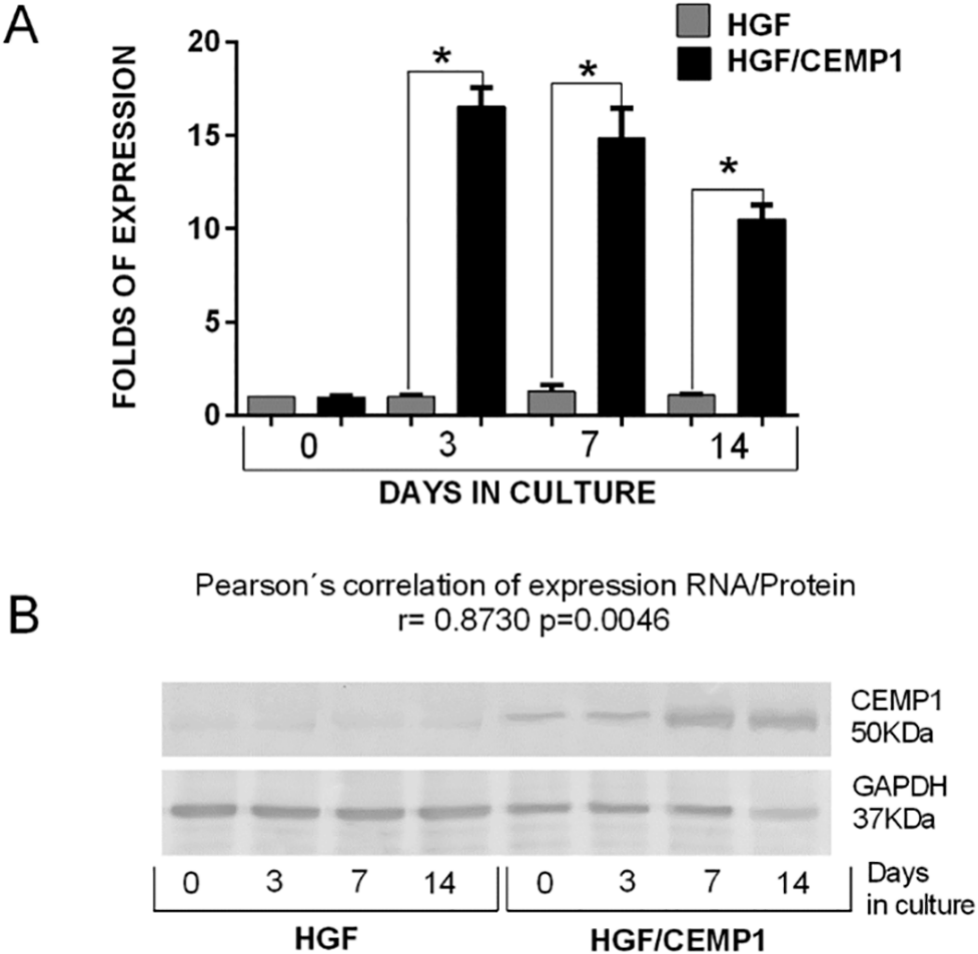
Expression of CEMP1 in HGF/CEMP1. The expression of CEMP1 after transfection in HGF and HGF/CEMP1 was evaluated using quantitative real time PCR at 3, 7 and 14 days. Fold increase of CEMP1 expression are represented, the graph shows that expression was higher in HGF/CEMP1 in all time points. * p = 0.0001 (A). CEMP1 protein expression levels were corroborated by western blot assays (B).

The expression of CEMP1 in HGF/CEMP1 and HGF cells at 0, 3, 7 and 14 days was determined using quantitative Real-time PCR and Western blot analysis as shown in **Fig 1.** The data shows background expression of CEMP1 in both cell lines at day 0, however, after 3 days in culture, CEMP1 expression was significantly higher in the HGF/CEMP1 while no changes were seen in the HGF cells alone, thus demonstrating that both, mRNA and protein are expressed in these cells.

### Genome-wide expression profiles of HGF-CEMP1 vs HGF

**Fig 2:**
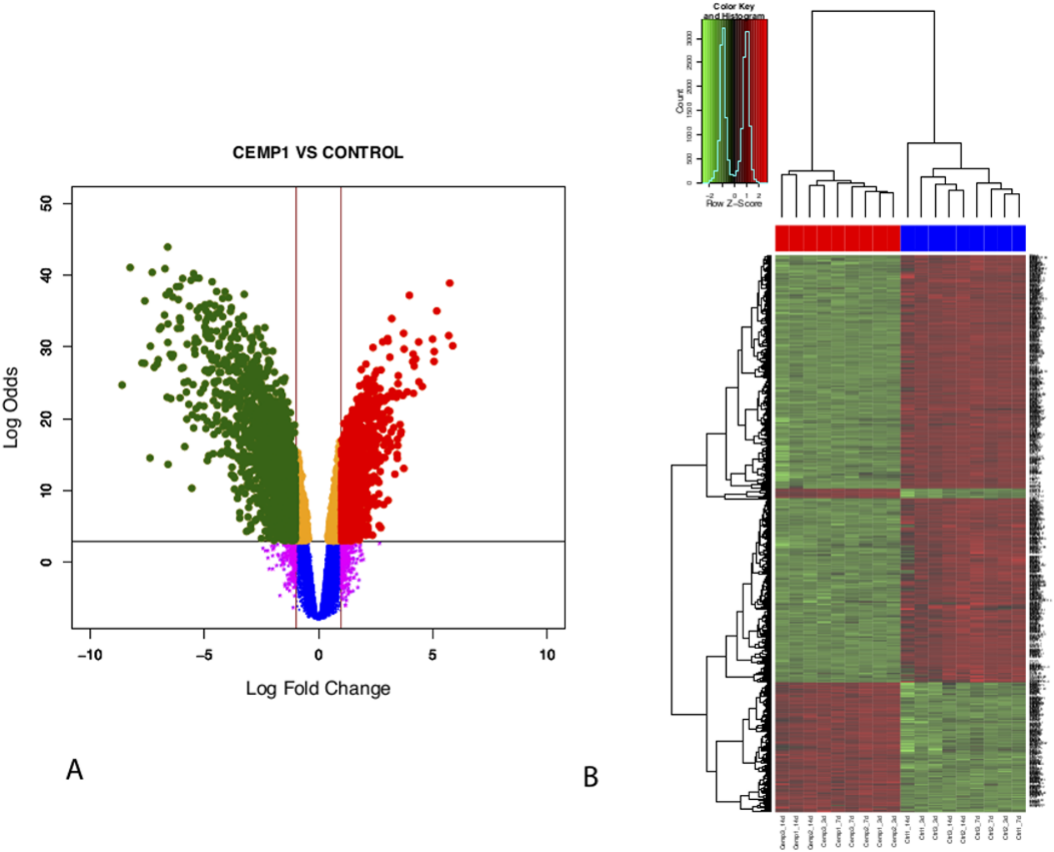
Genome-wide expression profiles HGF-CEMP1 vs. HGF. Total RNA was extracted from HGF and HGF/CEMP1 cell lines. Results from 3 independent experiments were subjected to a microarray analysis. (A). Volcano plot of 1039 differentially expressed genes between HGF-CEMP1 vs. HGF controls. Genes were selected on the basis of the significance of the differential gene expression (vertical red line; *P* < 0.001) and the level of induction or repression (horizontal red line; fold-change ≥ 2). (B). Heat-map of genes differentially expressed in microarray analysis.

In order to gain insight into the biological functions of CEMP1, the gene expression profile of HGF/CEMP1 cells was analyzed using Human Gene 1.0 ST platform (Affymetrix) and compared with HGF controls, and the data was analyzed with R 2.11.0 software. Analysis of the original normalized data set revealed a total of 1,039 genes that were differentially expressed with a p < 0.0001 and at least a 2 FC as shown in **Fig 2**. Amongst these genes, 260 were upregulated and 779 downregulated (75%) suggesting that CEMP1 expression in HGF fibroblasts induces transcriptional inactivation. The full list of up and downregulated genes in HGF/CEMP1 cells is provided as supplementary materials (**S1 Table**). No significant changes in gene expression were found at different time points (3d, 7d, and 14d), suggesting that all major changes in transcription due to CEMP1 expression take place at earlier time points.

### Functional annotations

**Fig 3:**
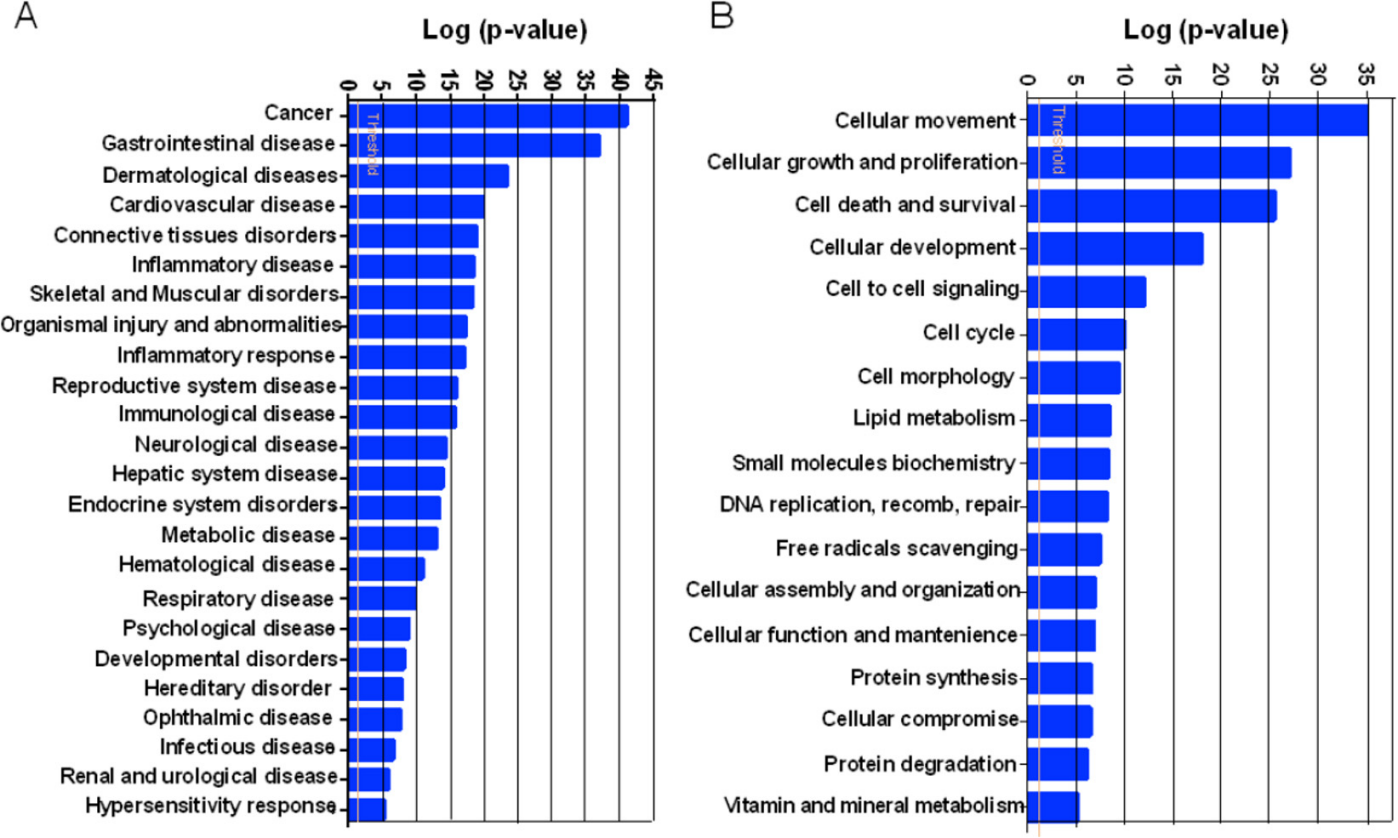
Functional analysis of differentially expressed HGF-CEMP1 genes. Ingenuity pathway analysis showed Diseases and Bio-Functions significantly modulated by CEMP1 expression in HGF/CEMP1 cells (p < 0.05). A total of 1,390 genes were differentially expressed: 260 were up-regulated and 779 were down-regulated. The most significant diseases (A) and bio-functions (B) are shown. Genes that met the p-values <0.05 threshold were associated with biological functions or diseases in the Ingenuity Pathway knowledge. Fischer's exact test was used to calculate the p-value to determine the probability that each biological function/disease assigned to the data set is not due to chance alone.

The biological relevance of CEMP1 expression was analyzed using DAVID (Database for Annotation, Visualization and Integrated Discovery) and IPA (IPA; www.ingenuity.com) for functional annotation clustering. Differentially expressed genes were classified according to their molecular and cellular function. DAVID analysis reported 39 functional clusters with an enrichment score greater than 2. The main clusters were cellular development, proliferation and cellular growth, cell death, and cell cycle. Genes that met the p-values <0.05 threshold were associated with biological functions or diseases in the Ingenuity Pathway knowledge. Fischer's exact test was used to calculate the p-value to determine the probability that each biological function/disease assigned to the data set is not due to chance alone. The most significant diseases (A) and bio-functions (B) are shown in **Fig 3**.

The main molecular and cellular functions associated with these set of genes were Cellular Movement (p = 1.37E-35–2.17E-06, 234 molecules), Cellular Growth and Proliferation (p = 2.58E-28–2.05E-06, 323 molecules), Cell Death and Survival (p = 1.29E-26–1.99E-06, 292 molecules), Cellular Development (p = 6.27E-19–1.56E-06, 285 molecules) and Cell-To-Cell Signaling and Interaction (p = 3.55E-12–2.02E-06, 227 molecules). The analysis showed that 599 molecules were associated with Cancer (p-value 1.62E-41–1.98E-06). Biological processes associated with upregulated genes were mainly cell growth and proliferation, cell death and survival, cellular development, cell morphology, cell-to-cell signaling, and molecules are associated with cancer (141 molecules). Downregulated genes also were involved in cellular proliferation, survival and development; however the main inhibited cellular function was movement (**Fig 3**).

The IPA Regulator Effects algorithm connects upstream regulators, dataset molecules and downstream functions or diseases. Using this algorithm, two main possible regulators were identified; the highest score for upstream regulators was CTNNB1, beta-catenin protein is an integral part of the canonical Wnt signaling pathway that plays a role in development as well as maintenance and renewal of stem cells, and the transcription factor E2F1, which mediate cell proliferation and apoptosis (**S1 Fig**).

### Transcriptional and translational level validation of key genes

**Fig 4:**
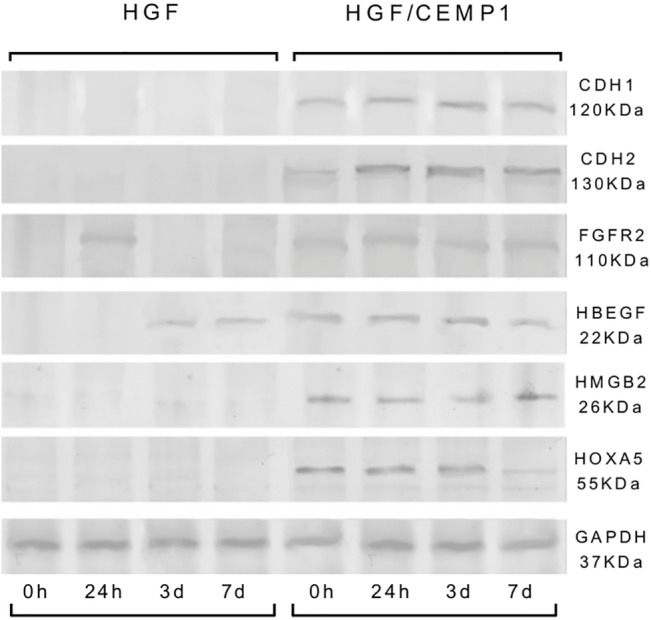
Western Blot validation of microarray results for key genes. Validation at the protein level by Western Blot was made at different time terms (basal, 24 hr, 3, and 7 days). Western blot demonstrated protein expression was higher in CEMP1 expressing cells than in controls.

In order to validate the differential expression of key genes across all the observed functional clusters, qRT-PCR was performed on 6 genes that are associated with oncogenesis; cell death, tissue development, proliferation, angiogenesis and invasion. The genes selected were: developmental transcription factor HOXA5 (p 7.45E-11 FC 3.29); cell-cell adhesion glycoproteins CDH1 (p 7.08E-11, FC 2.34) and CDH2 (p 1.45E-12, FC 4.15); the growth factor HBEGF (p 5.67E-09, FC 2.49); the growth factor receptor FGFR2 (p 1.33E-10, FC 2.02) and the growth factor that codifies for a chromatin associated protein HMGB2 (p 1.16E-12, FC 2.34) These genes were selected for further study because they were differentially expressed between CEMP1 expressing cells and controls and are known to be involved in oncogenesis, proliferation, cell death, and development. The findings indicated a good concordance of expression profiles between the microarray and qRT-PCR results (**Table 1**). These genes were also validated at the protein level by Western Blot. Expression was analyzed at different time terms (**Fig 4**) (basal, 24 hr., 3, and 7 days). The results show higher protein expression in CEMP1 expressing cells than in controls.

**Table 1:** RT-qPCR validation set.

Gene	HGF	HGF/CEMP1	p value
**CEMP1**	1,78	46,92	< 0.0001
**CDH2**	28,19	46,78	< 0.002
**FGFR2**	0,42	22,67	< 0.0001
**HBEGF**	0,39	25,15	< 0.0001
**HMGB2**	1,40	10,71	< 0.0001
**HOXA5**	0,035	28,56	< 0.0001
**CDH1**	0.0023	8.23	< 0.0001

RT-qPCR was performed for CEMP1 and a total of 6 genes that represent the main functional clusters related with oncogenesis; cell death, tissue development, proliferation, angiogenesis and invasion. The findings indicated a good concordance of expression profiles between the microarray and qRT-PCR results.

The expression of the key genes was quantified using qRT_PCR in a cementoblastic-like cell line (CEM), which normally express CEMP1. CEM cells showed only basal expression of most of the mRNAs, only CDH2 exhibited a higher expression (**S2 Fig).**

**Fig 5:**
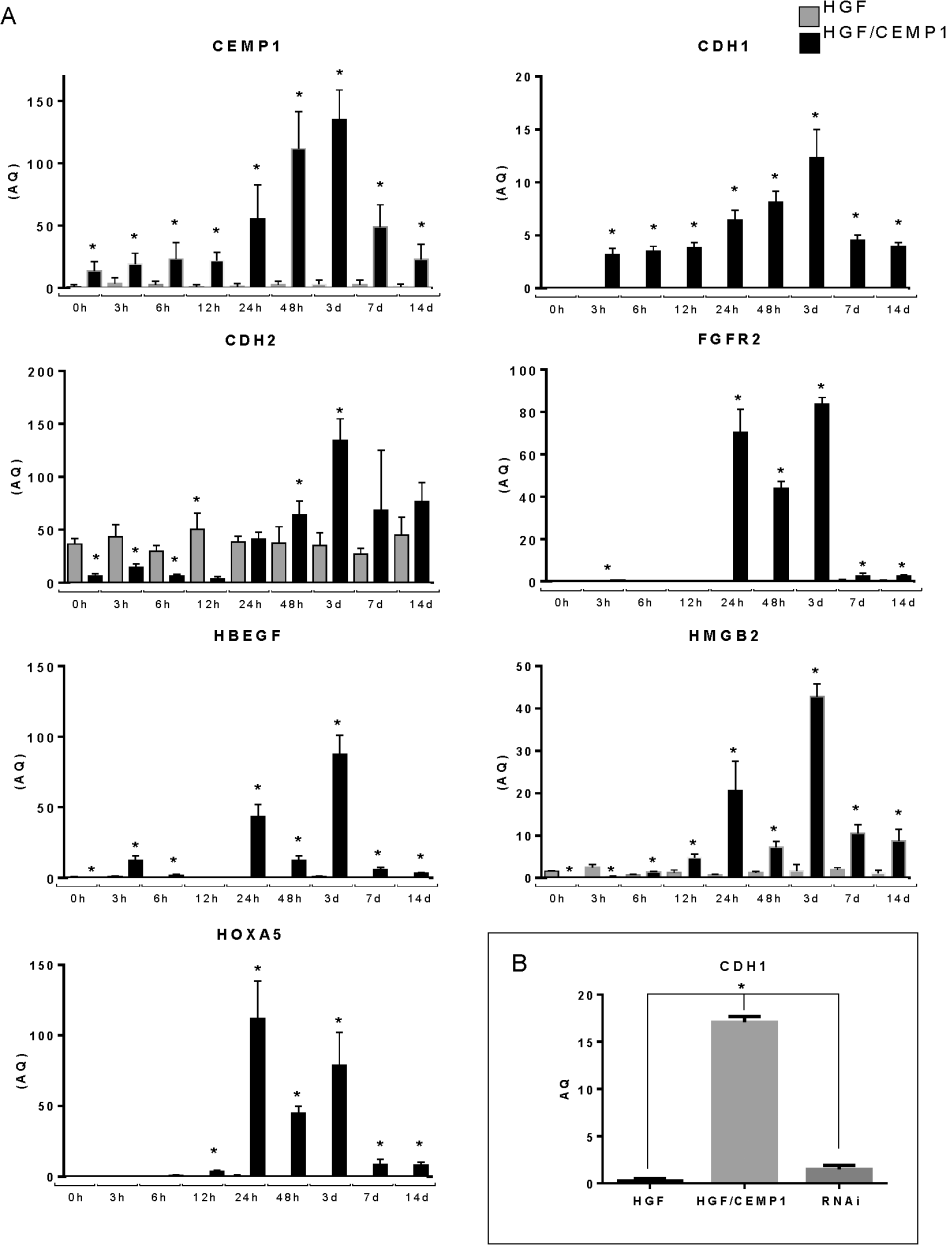
Time series expression pattern of key genes. **Expression of CDH1 before and after RNAi of CEMP1.** Expression patterns of the key genes were obtained in an independent time series experiment at 3, 6, 12, 24, 48, hours and 3, 7 and 14 days. The major expression changes as a result of CEMP1 overexpression initiated 24hrs (A). The relationship between CEMP1 and CDH1 was evaluated by RT-qPCR before and after knockdown of CEMP1 expression by RNAi. The RNAi directed against CEMP1 resulted in a significant decrease of CDH1 expression (B).

Time series analysis did not show any significant differences in expression at 3, 7 and 14 days, thus, we hypothesize that the phenotype changes resulting from CEMP1 overexpression take place at earlier times. Therefore, the expression patterns of the key genes were evaluated in an independent time series experiment at 3, 6, 12, 24, 48, hours and 3, 7 and 14 days by qRT-PCR. Although CEMP1 is over expressed in the experimental cells, the highest level of expression is showed in day 3 decreasing thereafter. All genes tested showed significant increase in expression at 24 hrs after transfection with CEMP1 and the highest expression at day 3, with the only exception of HOXA5 showing highest expression at 24 hrs. Similar to CEMP1, the expression of the selected 6 genes decreased at 7 and 14days. CDH1 and CDH2 showed a similar expression pattern as CEMP1, however, CDH2 showed expression in non-transfected cells as well (**Fig 5A**).

To further demonstrate the relationship between CEMP1 and CDH1 we decided to evaluate the expression of CDH1 before and after knockdown of CEMP1 expression by RNAi. The RNAi directed against CEMP1 resulted in a significant decrease of CDH1 expression (**Fig 5B**).

### CEMP1 induces anchorage-independent growth

**Fig 6:**
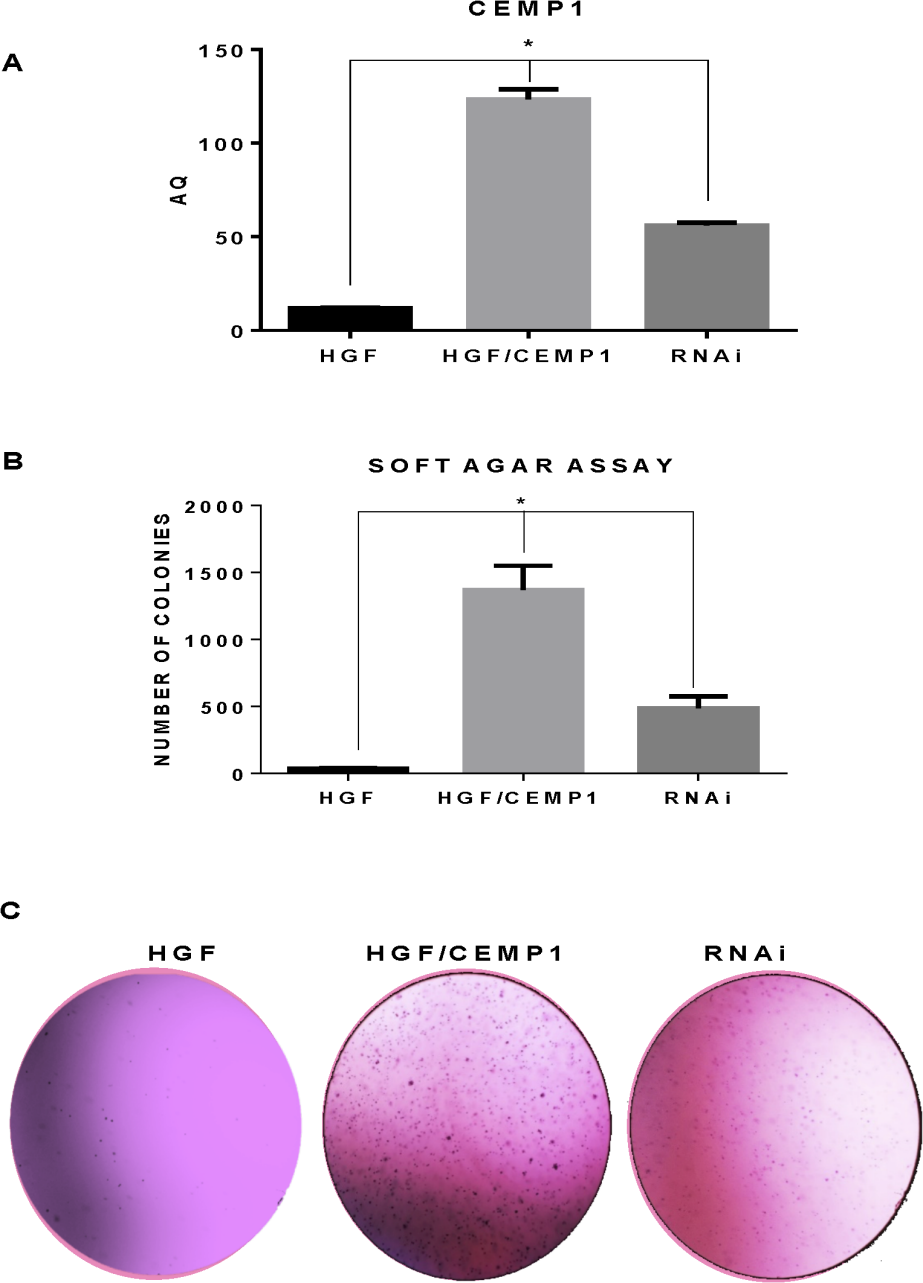
CEMP1 induces anchorage-independent growth in HGF cells. Cells were plated in a soft agar assay and the number of colonies was evaluated 21 days after the seeding. Representative plates of HGF, HGF/CEMP1 and HGF/CEMP/RNAi are presented. RT-qPCR showed that RNAi against CEMP1 decreased its expression in more than 50% at transcriptional level (A). All soft agar assays were performed in triplicates, and the mean ± standard deviations are presented (B and C).

In order to determine if CEMP1 has transforming capacity we performed an anchorage-independent growth assay. HGF cells were stably transfected with CEMP1 constructs and cells transfected with empty vectors were used as controls. After 21 days, macroscopic colonies could be detected in the dishes containing HGF/CEMP1 transfected cells (**Fig 6**). The number of colonies was significantly abundant (40.41 folds) in CEMP1/HGF compared to controls (p < 0.0001) (**Table 2**).

**Table 2:** CEMP1 induces anchorage-independent growth in HGF.

*CELL LINE*	# OF COLONIES AVERAGE PER WELL	# OF COLONIES PER WELL MIN AND MAX	FOLDS OF CEMP1 INDUCTION COMPARED TO CONTROL
*HGF*	33.9	26–41	1.0
*HGF/CEMP1*	1370	1012–1620	40.41
*HGF/CEMP1/ RNAi*	482.2	352–623	14.22

To eliminate the possibility that the transformation of HGF cells was caused by a nonspecific effect, we tested whether knockdown of CEMP1 expression by RNAi could influence the ability of HGF cells to form colonies in soft agar. Forty-eight hours after transfection of RNAi, the cells were placed into medium with soft agar, and colonies were counted after 3 weeks. Inhibition of CEMP1 expression with RNAi resulted in a significant decrease (64.8%, p < 0.0001) in colony formation in HGF cells (**Fig 6B**). These results showed that reduction in CEMP1 expression decreased the ability of HGF cells to form colonies in soft agar, confirming that CEMP1 is responsible for this specific phenotype.

**Fig 7:**
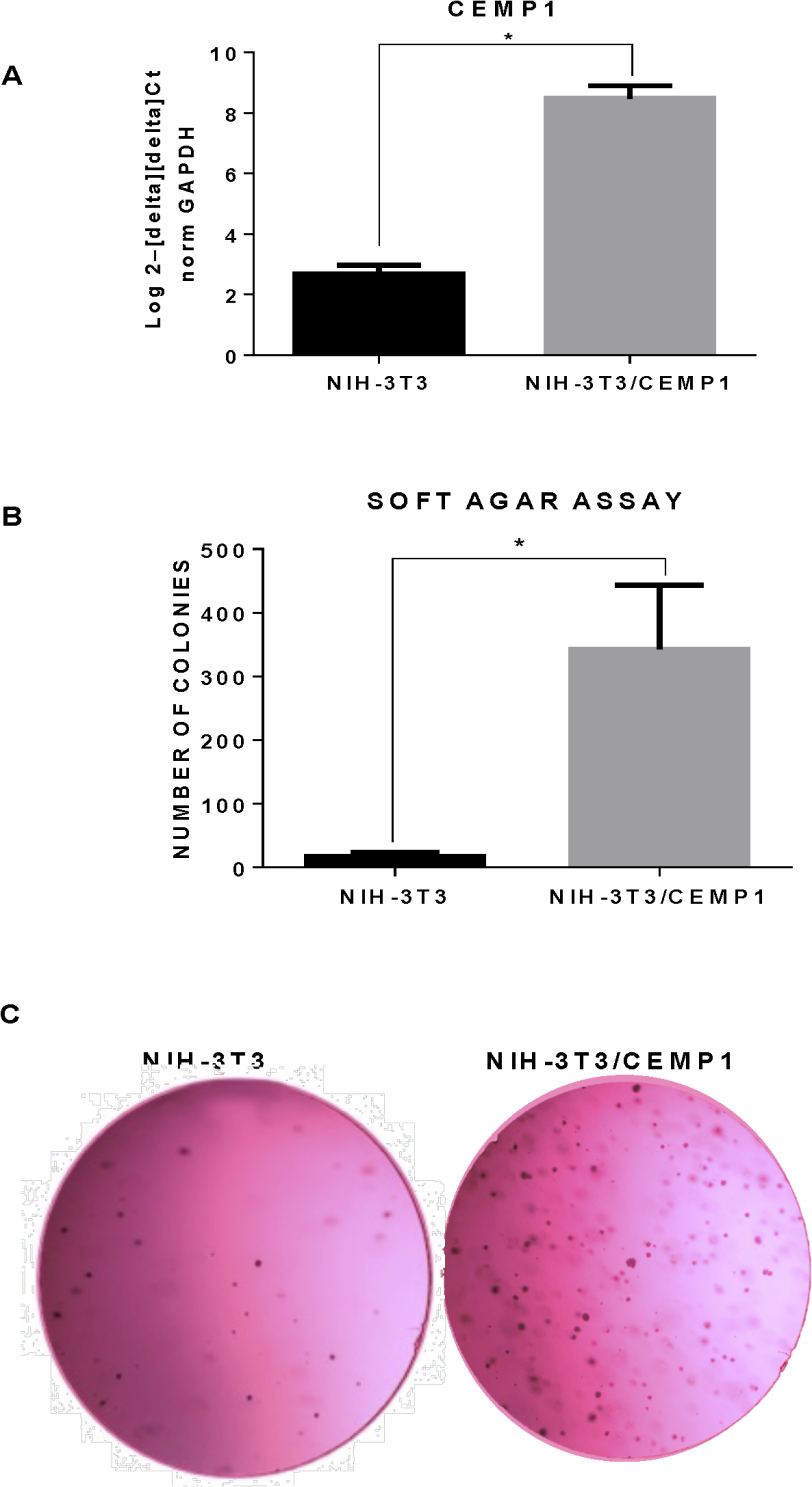
CEMP1 induces anchorage-independent growth in NIH-3T3cells. Cells were plated in a soft agar assay and the number of colonies was evaluated 21 days after the seeding. RT-qPCR supported overexpression of CEMP1 in NIH-3T3 in experimental group (A).Representative plates of NIH-3T3 and NIH-3T3 /CEMP are presented. All soft agar assays were performed in triplicates, and the mean ± standard deviations are presented (B and C).

To corroborate the capability of CEMP1 to induce anchorage-independent cell growth we used another type of cell line; NIH-3T3 cells. Results from qRT-PCR (**Fig 7A**) and Western blot (**S3 Fig**) revealed that after transfection with CEMP1, NIH-3T3 fibroblasts showed a 3 fold increase at the mRNA level and 4 fold increase at the protein level of CEMP1 expression as compared to controls. The anchorage-independent cell growth analysis showed macroscopic colonies in the dishes containing transfected cells and the colonies were significantly more abundant (20.4 folds) (**Table 3**) in NIH-3T3/CEMP1 cells compared to controls (p < 0.0001) (**Fig 7B and 7C**).

**Table 3:** CEMP1 induces anchorage-independent growth in NIH3T3.

CELL LINE	# OF COLONIES AVERAGE PER WELL	# OF COLONIES PER WELL MIN AND MAX	FOLDS OF CEMP1 INDUCTION COMPARED TO CONTROL
NIH3T3	16.5	12–34	1.0
NIH3T3/ CEMP1	338	245–584	20.4

### CEMP1 is expressed in cancer cells

**Fig 8:**
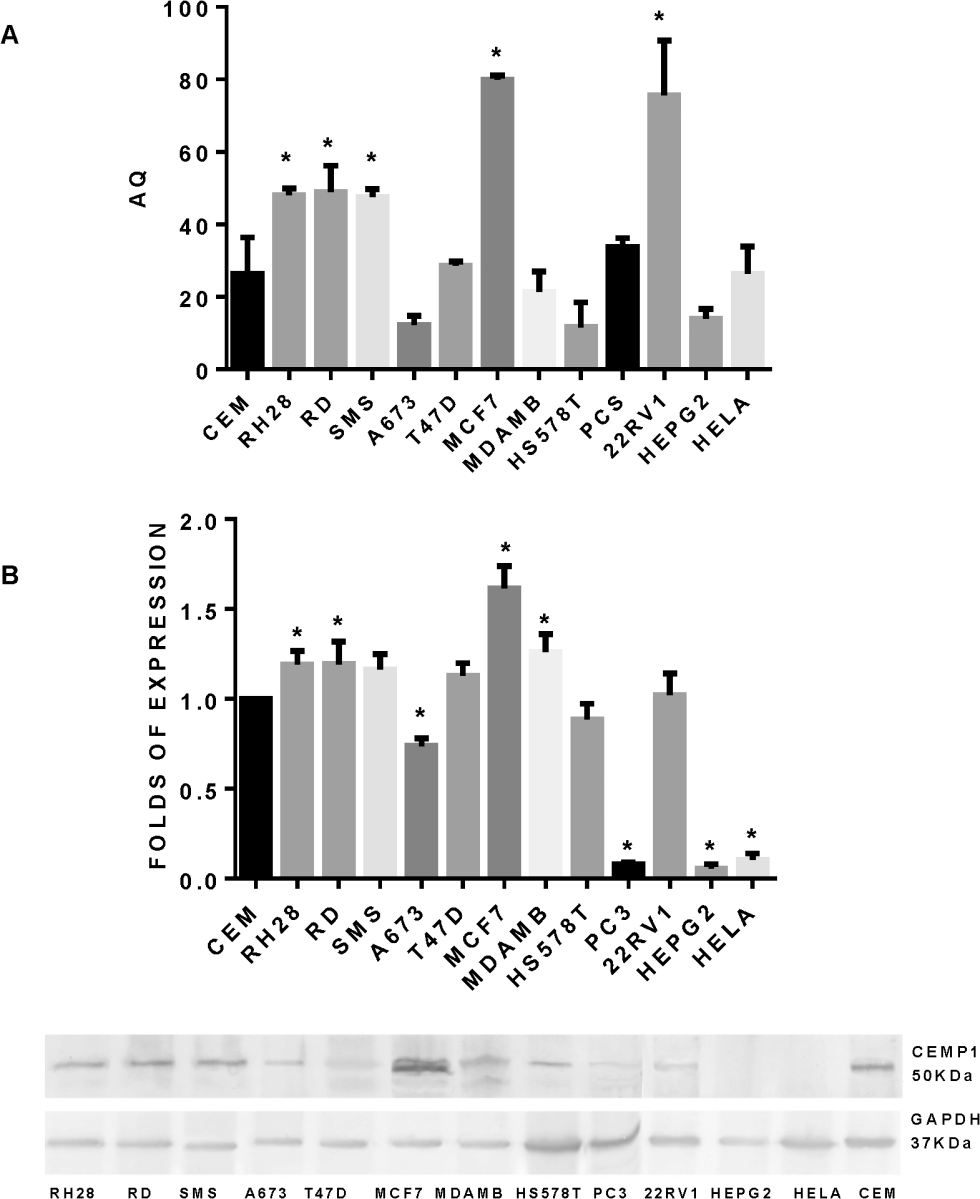
CEMP1 is expressed on cancer cells. CEMP1 expression screening on a variety of human cancer cell lines by qRT-PCR. the mean ± standard deviations are presented (A). Western Blot was developed and Fold of CEMP1 expression compared against cementoblastic-like cell line (CEM) is presented (B).

Previous reports have shown the expression of CEMP1 is limited to cementoblasts and their progenitor cells. Given the results presented above suggesting an oncogenic role for CEMP1, we decided to evaluate CEMP1 expression on a variety of human cancer cell lines by qRT-PCR and Western Blot. Cell lines analyzed were: breast cancer (MCF7, T47D, MDAMB, HS578T), bone and soft tissue sarcomas (RD, SMS-CTR and RH28, A673), prostate cancer (PC3, 22RV1), hepatocellular carcinoma (HEPG2) and cervix adenocarcinoma (HELA). The expression was compared with a cementoblastic-like cell line (CEM)[23]. The results show CEMP1 expression in all cancer cell lines tested, both at the mRNA and protein levels. The breast cancer cell line MCF7 showed the highest CEMP1 expression (0.6 fold), followed by two rhabdomyosarcoma cell lines (RH28 (0.18 fold) and RD (0.18 fold)). Lowest expression was observed in the prostate cell line PC3 (-0.91 folds) and hepatocellular carcinoma HEP2G (-0.94 fold) cell lines. However all cancer cell lines tested, showed increase expression of CEMP1 at some level, and some of them expressed CEMP1 at higher levels than the cementoblastic-like cell line (**Fig 8**).

Since CEMP1 is expressed in several cancer cell lines, we wanted to expand these studies to human tumor samples and their normal tissue counterparts contained in publicly available microarray databases. CEMP1 expression was found in additional oral, breast, cervical, prostate and lung cancer samples, as well as leukemia samples (**S2 Table**). Further information was obtained from the Catalogue of Somatic Mutations in Cancer (COSMIC v70) (http://www.sanger.ac.uk/cosmic), a publicly available resource, providing information on somatic mutations implicated in human cancer. It is well known that oncogenes can become activated by different mechanisms; the most common are gene amplification, point mutations and chromosomal rearrangements. The analysis reported that from a total of 19345 unique samples, 21 samples had mutations in CEMP1 and the most frequent mutation type was missense substitution (80.95%) of which 9 (42.86%) were G>A transitions. Copy number variations (CNV) gain in 16p13.3 region was reported in 44 unique samples; overexpression was present in 203 samples and the highest frequency in tissue samples were Breast (8.79%), Kidney (7.69%), Large intestine (7.29%), Ovary (6.25%), Endometrium (5.4%), Lung (5.38%), Hematopoietic and lymphoid (5.2%), Central nervous system (5.13%). Breast cancer samples showed the highest percentage of CNV gains (3.62%) and urinary tract tissue showed the highest percentage of point mutations (0.82%).

## Discussion

This study reports the use of microarrays to evaluate the potential biological effects of CEMP1 transduced into human gingival fibroblasts. Using two enrichment analysis tools, DAVID and IPA, we found a statistically significant increase in the expression of a high numbers of genes associated with cellular development, proliferation, growth, cell death and cell cycle. These results were surprising since previous studies have shown that CEMP1 plays a key role during the mineralization process by nucleating octacalcium phosphate crystals. Furthermore, we have also shown that CEMP1 promotes bone regeneration in critical-size defects in rat calvaria confirming the osteoinductive properties of this protein and its potential to be used for bone tissue regeneration [11].

Other molecules, particularly growth factors like TGF-β, BMPs, etc., have shown promising clinical successes for tissue regeneration; however their use is still controversial due to their oncogenic potential since several studies have reported overexpression of these molecules in different types of cancer [24]. On the other hand, it has been proven that the expression of oncogenic molecules like c-myc, and c-fos are crucial for tissue regeneration in the first hours after hepatectomy, and other molecules like c-met and jun are used in order to regenerate peripheral axons and myocites [25–27]. Therefore, it is important to first establish optimal concentration and timing for the use of these molecules, in order to have a successful clinical outcome without side effects such as tumor development.

Perhaps CEMP1 can fall in the same category as some of these growth factors. Based on the results obtained in this study, the cellular functions altered by the presence of CEMP1 appear to be associated with some aspects of cancer development, thus sustaining the idea that CEMP1, similar to some growth factors, could also function as an oncogene. This possibility is further supported by the fact that the original CEMP1 protein was isolated and characterized from a human cementoblastoma, a neoplasm of odontogenic ectomesenchyme origin.[28, 29].

CEMP1 was originally identified as a cementum specific protein, and its expression is restricted to cementoblasts, some periodontal ligament cell subpopulations and mesenchymal stem cells located paravascularly in the PDL [2, 23]. Transfection of CEMP1 gene into ‘non-mineralizing’ cells such as human gingival fibroblasts enhanced their proliferation rate, induced the expression of cementum/bone-associated proteins and extracellular matrix mineralization [10]. Our results show that CEMP1 expression in fibroblast growing in non-mineralizing media changed normal mouse and human cells into transformed cells, showing cancerous phenotypes in all tested in vitro tumor-forming assays. Previous studies have shown expression of CEMP1 in breast and prostate cancer with bone metastasis and in MCF-7 and PC3 cell lines [30]. Furthermore, key molecules related to oncogenesis, cell death, tissue development, proliferation, angiogenesis and invasion like HOXA5, CDH1, CDH2, HBEGF, FGFR2 and HMGB2, are overexpressed as a result of CEMP1 expression in fibroblasts cells. Regarding to Cadherins, they play an important role in cell adhesion forming *adherens* junctions. CDH1 and CDH2 are expressed in most normal tissues and reduced or loss of expression in cancer cells is well documented. However, CDH2 overexpression has been reported in hepatocellular carcinoma [31]. CDH1 is found in metastatic breast and prostate cancer cells in bone [32, 33]. Furthermore, in our time series assays, CDH1 showed a similar expression pattern to CEMP1 and knockdown of CEMP1 expression by RNAi resulted in a significant decrease in CDH1 expression. These results suggest that CEMP1 and CDH1 could co-participate in the metastatic process although additional work is required to further test this hypothesis. HOXA5 is highly expressed in gastric cancer cell lines and oral squamous cell carcinomas (OSCC) tissues and cell lines [34]. HBEGF is a potent mitogen for keratinocytes, hepatocytes, smooth muscle cells and fibroblasts, and its expression is elevated in human cancers, including hepatocellular and gastric carcinoma, breast carcinoma, melanoma, colon cancer, pancreatic cancer, glioma and glioblastoma [35]. Overexpression of HBEGF is induced by Ras and Raf oncogenes [36]. HMGB2, is highly expressed during embryogenesis, but has limited expression in adult tissues, mainly in lymphoid organs and testes. Overexpression of HMGB2 has been recognized in several types of tumors, including skin cancer [37], hepatocellular carcinoma [38], bladder carcinoma and had been correlated with tumor progression and angiogenesis [39]. Aberrant expression of FGFR2 can contribute to the proliferation of some malignant cancers such as gastric cancer [40], prostate cancer [41] and esophageal carcinoma [42] and facilitates cell survival in many different cancers including prostate cancer, breast cancer, and gastric cancer [43].

Another interesting fact about CEMP1 is that it maps on human chromosome 16, more precisely, at 16p13.3. Amplification of the 16p13.3 region has been reported in multiple solid tumors, like prostate, breast, and lung cancer as well as in pediatric tumors like glioblastoma multiforme and osteosarcoma [44–46]. Published studies have shown that 16p13.3 gain is associated with poor survival in breast cancer patients and a cytogenetic study of lung tumors found that the 16p13.3 gain is associated with poor differentiation and late stage disease [47–49]. Gain on 16p and loss of 16q was reported in a group of male breast cancers with low propensity to develop lymph node metastases [50]. In previous studies, PDK1 was identified as candidate oncogene in this chromosomal region, but the involvement of additional causative oncogenes could be expected [47].

There is not much information related to the role of CEMP1 in cancer. Our *in silico* COSMIC analysis showed that the frequency of CEMP1 activation is low, a few samples showed amplification, point mutations and the vast majority had CEMP1 overexpression. It is well known that cancer is a disease caused by accumulated genetic and epigenetic changes, but only a subset will contribute to tumor development. Mutations that conferred growth advantage to cancer cell and are positively selected in the microenvironment of the tissue in which the cancer arises are called driver mutations [51]. To be able to distinguish between these “driver” mutations from the neutral “passenger” mutations is crucial to gain insight in cancer biology and improve patient treatment. One important filter that has been used is mutation frequency, however, frequency will differ between tumor-types and requires better estimates of the Background mutation rate (BMR) in order to report rarely mutated genes with high confidence [52, 53]. Different groups are testing different approaches to identify true driver genes, like mutation patterns, positive selection across tumors, rate of cancer mutations above background, clustering patterns of mutations, and functional impact [54, 55]. More research needs to be carried on to fully understand the role of CEMP1, if any, on cancer development.

## Conclusions

In previous communications we have reported that CEMP1 is a potentially powerful therapeutic molecule for tissue mineralized tissue regeneration. The new information reported in this study however cautions about a possible oncogenic potential for this molecule as well. Depending on the environmental conditions, CEMP1 can induce biomineralization, nevertheless, our microarray expression and functional studies results are consistent with the possiblility that CEMP1 could act as a novel oncogene. CEMP1 shows some of the hallmarks of an oncogene: ectopic expression of CEMP1 transforms NIH 3T3 fibroblasts and human fibroblast, and confers anchorage-independent growth in soft agar. Nevertheless future work will be needed to discover if CEMP1 is a passenger of the 16p13.3 locus or a significant oncogenic molecule.

## Supporting Information

S1 Fig IPA Regulatory effects. Connections to upstream regulator with the highest score is CTNNB1.
(TIF)

S2 Fig Expression of key genes in Cementoblastic-like cells. The expression of key genes was evaluated at 3 days by triplicated using qRT-PCR. The CEM cell showed expression of most of the molecules in a basal fashion, only CDH2 exhibited a higher expression.
(TIF)

S3 Fig Expression at protein level of HGF/CEMP1/RNAi and NIH-3T3/CEMP1. The expression of CEMP1 after transfection in NIH-3T3 and NIH-3T3/CEMP1 was evaluated using Western Blot at 3 days by triplicated. The expression levels increased more than 3 times when compared to control.
(TIF)

S1 Table List of up and downregulated genes in HGF/CEMP1 vs HGF. (DOCX)

S2 Table Publicly available microarray data. CEMP1 overexpression in human cancer samples.
(DOCX)
