# Supplementary figures and images for: CEMP1 Induces Transformation in Human Gingival Fibroblasts
Source: PLoS One. 2015 May 26;10(5):e0127286. doi: 10.1371/journal.pone.0127286 (PMC4444236; doi:10.1371/journal.pone.0127286)

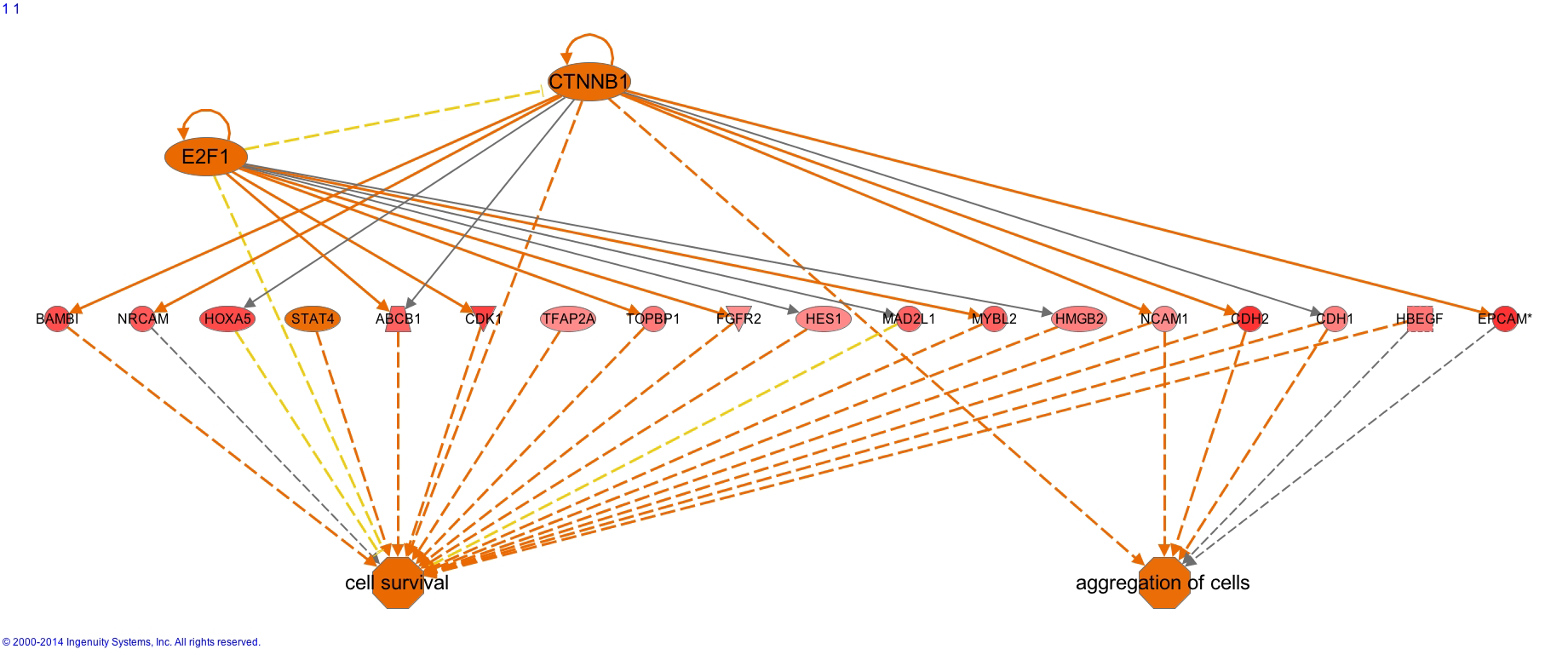

Supplement: S1 Fig — Connections to upstream regulator with the highest score is CTNNB1. (TIF) [file pone.0127286.s001.tif]

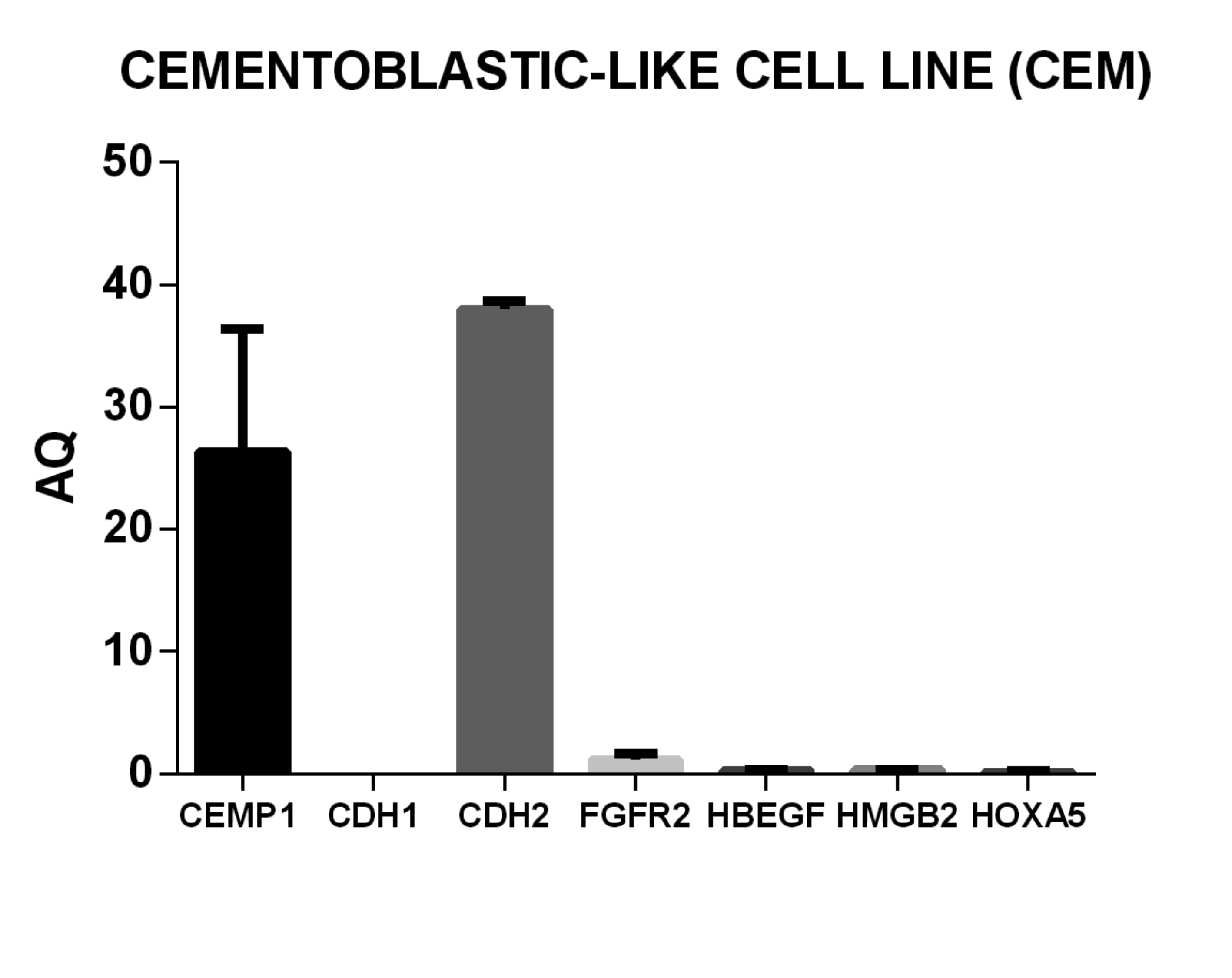

Supplement: S2 Fig — The expression of key genes was evaluated at 3 days by triplicated using qRT-PCR. The CEM cell showed expression of most of the molecules in a basal fashion, only CDH2 exhibited a higher expression. (TIF) [file pone.0127286.s002.tif]

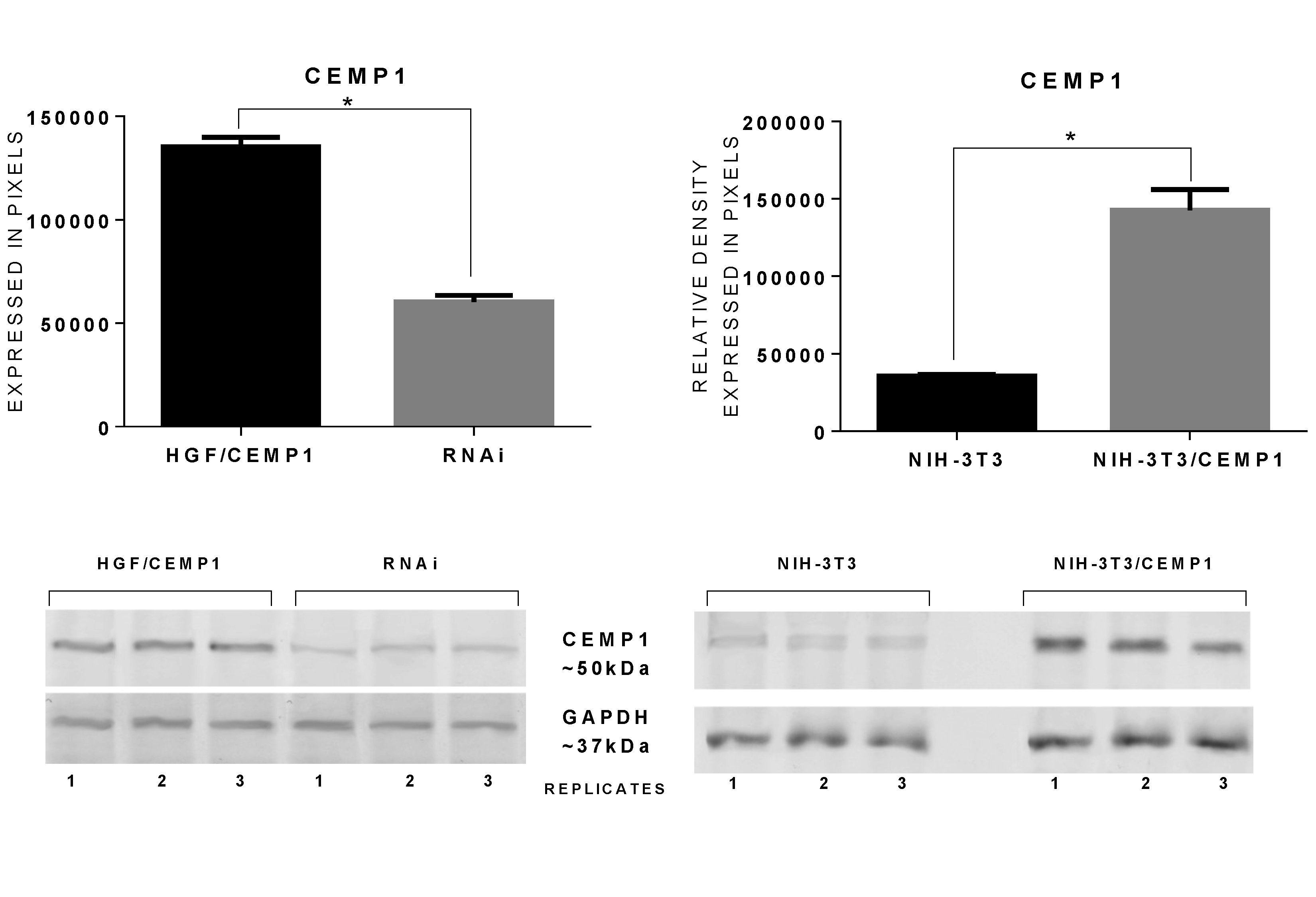

Supplement: S3 Fig — The expression of CEMP1 after transfection in NIH-3T3 and NIH-3T3/CEMP1 was evaluated using Western Blot at 3 days by triplicated. The expression levels increased more than 3 times when compared to control. (TIF) [file pone.0127286.s003.tif]
