# Supplementary material for: CEMP1 Induces Transformation in Human Gingival Fibroblasts
Source: PLoS One. 2015 May 26;10(5):e0127286. doi: 10.1371/journal.pone.0127286 (PMC4444236; doi:10.1371/journal.pone.0127286)
Supplement: S1 Table — (DOCX) [file pone.0127286.s004.docx]

Table S1.

|  | **symbols** | **ID** | **logFC** | **AveExpr** | **t** | **P.Value** | **adj.P.Val** | **B** |
| --- | --- | --- | --- | --- | --- | --- | --- | --- |
|  |  |  |  |  |  |  |  |  |
|  | **Downregulated** |  |  |  |  |  |  |  |
| 1 | NA | 8165661 | -8.5944206 | 8.93855717 | -20.66517 | 4.40E-15 | 5.45E-13 | 24.7134091 |
| 2 | MMP1 | 7951271 | -8.2406017 | 8.82243687 | -48.665544 | 1.86E-22 | 2.41E-18 | 41.0759807 |
| 3 | NA | 8165665 | -7.7184643 | 9.10258405 | -24.160522 | 2.09E-16 | 4.45E-14 | 27.7807358 |
| 4 | NA | 8165690 | -7.6123444 | 9.05937731 | -24.109299 | 2.18E-16 | 4.57E-14 | 27.7390882 |
| 5 | MPLKIP | 8165648 | -7.6001028 | 6.90024427 | -37.785505 | 2.96E-20 | 3.99E-17 | 36.429727 |
| 6 | NA | 8165646 | -7.3727435 | 9.64831468 | -12.177944 | 8.93E-11 | 2.46E-09 | 14.5671476 |
| 7 | NA | 8165644 | -7.3547709 | 8.16194205 | -27.225853 | 2.00E-17 | 6.32E-15 | 30.120301 |
| 8 | MMP3 | 7951284 | -7.2771326 | 8.29045332 | -46.852537 | 3.99E-22 | 3.07E-18 | 40.3968357 |
| 9 | NA | 8165705 | -7.1848211 | 7.66727383 | -23.436147 | 3.79E-16 | 7.22E-14 | 27.1832683 |
| 10 | SH3KBP1 | 8165674 | -7.0619996 | 7.78986659 | -23.968229 | 2.45E-16 | 5.01E-14 | 27.623923 |
| 11 | GREM1 | 7982377 | -7.0144609 | 9.37979796 | -24.729133 | 1.32E-16 | 3.10E-14 | 28.2370859 |
| 12 | NA | 8165686 | -6.9604542 | 8.55688215 | -30.752409 | 1.79E-18 | 9.44E-16 | 32.490141 |
| 13 | PCDH18 | 8102792 | -6.8815329 | 7.51884709 | -31.23766 | 1.31E-18 | 7.86E-16 | 32.7930369 |
| 14 | NA | 7895026 | -6.7377811 | 6.45424168 | -34.063787 | 2.34E-19 | 1.94E-16 | 34.4595162 |
| 15 | TNFRSF11B | 8152512 | -6.7159261 | 7.82400205 | -48.222773 | 2.24E-22 | 2.41E-18 | 40.9131506 |
| 16 | DPP4 | 8056222 | -6.6633808 | 7.37618246 | -39.538479 | 1.20E-20 | 2.15E-17 | 37.2810209 |
| 17 | COL1A2 | 8134263 | -6.6208815 | 9.82370595 | -18.952451 | 2.34E-14 | 2.27E-12 | 23.019369 |
| 18 | GALNT5 | 8045835 | -6.5995642 | 7.54447942 | -57.391325 | 6.75E-24 | 2.18E-19 | 43.9333515 |
| 19 | NA | 7895246 | -6.593626 | 6.07861785 | -27.956061 | 1.18E-17 | 4.11E-15 | 30.6369865 |
| 20 | DCN | 7965410 | -6.5889038 | 7.50539601 | -31.947114 | 8.40E-19 | 5.43E-16 | 33.226692 |
| 21 | NA | 8174970 | -6.5846789 | 10.5130474 | -11.598134 | 2.13E-10 | 5.16E-09 | 13.6717392 |
| 22 | C1S | 7953603 | -6.5239512 | 8.35158583 | -40.547176 | 7.23E-21 | 1.51E-17 | 37.7508244 |
| 23 | NA | 7896438 | -6.4865421 | 7.94667214 | -25.69589 | 6.24E-17 | 1.65E-14 | 28.9887769 |
| 24 | COL6A3 | 8059905 | -6.4611107 | 8.43193509 | -18.763878 | 2.83E-14 | 2.65E-12 | 22.8239966 |
| 25 | NA | 7892972 | -6.4063195 | 6.53482492 | -25.188696 | 9.23E-17 | 2.26E-14 | 28.5981239 |
| 26 | IL6 | 8131803 | -6.3778069 | 8.03728962 | -38.907381 | 1.65E-20 | 2.67E-17 | 36.9797677 |
| 27 | NA | 7946563 | -6.3553884 | 6.36520463 | -25.629493 | 6.56E-17 | 1.72E-14 | 28.9380928 |
| 28 | WNT5A | 8088180 | -6.2118185 | 8.11550175 | -41.987767 | 3.59E-21 | 8.93E-18 | 38.397967 |
| 29 | NT5E | 8120967 | -6.1690479 | 7.918312 | -30.938538 | 1.59E-18 | 9.01E-16 | 32.6069387 |
| 30 | NA | 7895203 | -6.1318318 | 5.36442019 | -38.164899 | 2.43E-20 | 3.41E-17 | 36.6178632 |
| 31 | NA | 7893619 | -6.0729388 | 5.85644 | -33.352857 | 3.57E-19 | 2.62E-16 | 34.0552455 |
| 32 | FAP | 8056257 | -6.0692146 | 7.68377437 | -42.114651 | 3.38E-21 | 8.93E-18 | 38.453674 |
| 33 | NA | 7893956 | -6.0600559 | 8.79822835 | -18.875411 | 2.53E-14 | 2.41E-12 | 22.9397744 |
| 34 | CTSK | 7919815 | -6.0539527 | 8.64057391 | -32.60499 | 5.60E-19 | 3.77E-16 | 33.6194015 |
| 35 | NA | 8165676 | -6.0370486 | 8.14738789 | -30.983405 | 1.54E-18 | 8.91E-16 | 32.6349784 |
| 36 | TRPA1 | 8151341 | -5.9971914 | 6.76139383 | -23.256478 | 4.41E-16 | 8.10E-14 | 27.0321829 |
| 37 | SLC14A1 | 8021081 | -5.9439317 | 7.21760189 | -44.704829 | 1.02E-21 | 4.13E-18 | 39.5478034 |
| 38 | NA | 7895985 | -5.9187618 | 5.99576404 | -23.411974 | 3.87E-16 | 7.32E-14 | 27.1630097 |
| 39 | NA | 7896369 | -5.8808996 | 7.69386346 | -21.023539 | 3.15E-15 | 4.14E-13 | 25.0506479 |
| 40 | NA | 8165650 | -5.8538053 | 9.78378597 | -13.250072 | 1.94E-11 | 6.85E-10 | 16.1379654 |
| 41 | NA | 7892630 | -5.7828825 | 6.29292441 | -19.648436 | 1.17E-14 | 1.26E-12 | 23.7248102 |
| 42 | NA | 8165669 | -5.7609758 | 8.20374645 | -16.19631 | 4.66E-13 | 2.80E-11 | 19.9648358 |
| 43 | RPL15 | 8078270 | -5.7452302 | 6.41857955 | -27.621377 | 1.50E-17 | 5.00E-15 | 30.4019646 |
| 44 | NA | 7892820 | -5.7214564 | 7.99186808 | -21.20127 | 2.68E-15 | 3.67E-13 | 25.2158214 |
| 45 | NA | 8165653 | -5.6725826 | 8.05708383 | -18.004595 | 6.24E-14 | 5.28E-12 | 22.0181758 |
| 46 | ANPEP | 7991335 | -5.6432399 | 8.33399257 | -36.341686 | 6.45E-20 | 7.44E-17 | 35.6930702 |
| 47 | PRRX1 | 7907222 | -5.5779762 | 7.7359971 | -44.115838 | 1.33E-21 | 4.79E-18 | 39.3060016 |
| 48 | NA | 8165692 | -5.5434571 | 5.09430143 | -9.6245783 | 5.33E-09 | 8.02E-08 | 10.3528924 |
| 49 | NA | 8165682 | -5.5236649 | 6.97467743 | -16.718819 | 2.56E-13 | 1.72E-11 | 20.5787935 |
| 50 | NA | 8165698 | -5.4899508 | 6.77511111 | -20.725769 | 4.16E-15 | 5.21E-13 | 24.7708337 |
| 51 | THY1 | 7952268 | -5.4668927 | 8.48436436 | -46.447011 | 4.75E-22 | 3.07E-18 | 40.2403289 |
| 52 | NA | 7893573 | -5.4633759 | 6.30469459 | -15.203452 | 1.53E-12 | 7.66E-11 | 18.7478267 |
| 53 | ENPP2 | 8152522 | -5.4259708 | 7.7988366 | -26.605887 | 3.14E-17 | 9.15E-15 | 29.6700107 |
| 54 | GLIPR1 | 7957260 | -5.4132929 | 6.51470843 | -27.894714 | 1.24E-17 | 4.25E-15 | 30.5941315 |
| 55 | PTGS2 | 7922976 | -5.3992519 | 7.4759286 | -25.722806 | 6.11E-17 | 1.63E-14 | 29.0092845 |
| 56 | KGFLP1 | 8161455 | -5.38485 | 5.68207861 | -28.843583 | 6.38E-18 | 2.48E-15 | 31.2459958 |
| 57 | NA | 8165700 | -5.3572545 | 6.80614466 | -17.482557 | 1.09E-13 | 8.44E-12 | 21.4455493 |
| 58 | GPNMB | 8131844 | -5.3338729 | 8.78777094 | -44.911067 | 9.32E-22 | 4.13E-18 | 39.6315354 |
| 59 | MMP2 | 7995681 | -5.3164897 | 9.55322801 | -35.175286 | 1.24E-19 | 1.21E-16 | 35.0728224 |
| 60 | MT2A | 7995783 | -5.2984521 | 9.77943357 | -16.358352 | 3.86E-13 | 2.41E-11 | 20.1571247 |
| 61 | NA | 8111210 | -5.2743913 | 9.09392852 | -28.074431 | 1.09E-17 | 3.85E-15 | 30.7193937 |
| 62 | IFITM1 | 7937335 | -5.2556659 | 8.04815484 | -33.442676 | 3.38E-19 | 2.54E-16 | 34.1068531 |
| 63 | THBS2 | 8130867 | -5.2270759 | 9.28086785 | -44.814591 | 9.74E-22 | 4.13E-18 | 39.5924262 |
| 64 | CCL2 | 8006433 | -5.2052809 | 8.66827064 | -17.855515 | 7.32E-14 | 5.94E-12 | 21.8562355 |
| 65 | SLIT2 | 8094301 | -5.1817315 | 7.18223983 | -35.99549 | 7.81E-20 | 8.49E-17 | 35.5113903 |
| 66 | HAS2 | 8152617 | -5.165663 | 8.14147533 | -19.564572 | 1.27E-14 | 1.35E-12 | 23.6410807 |
| 67 | NA | 7894974 | -5.0381419 | 7.28517459 | -16.427885 | 3.56E-13 | 2.25E-11 | 20.2391118 |
| 68 | NA | 7892803 | -5.0373711 | 9.19264038 | -20.152873 | 7.15E-15 | 8.25E-13 | 24.2213078 |
| 69 | NA | 7893264 | -5.0115926 | 8.75125499 | -17.799006 | 7.77E-14 | 6.23E-12 | 21.7945245 |
| 70 | SLC16A6 | 8017843 | -4.988757 | 7.76405114 | -26.637398 | 3.07E-17 | 9.02E-15 | 29.6931622 |
| 71 | NA | 7895578 | -4.9789316 | 5.82112526 | -26.396371 | 3.67E-17 | 1.05E-14 | 29.5153432 |
| 72 | ITGA8 | 7932254 | -4.9787004 | 6.9005309 | -31.287641 | 1.27E-18 | 7.75E-16 | 32.8239417 |
| 73 | C3 | 8033257 | -4.9640762 | 7.99164849 | -36.737909 | 5.19E-20 | 6.46E-17 | 35.8985671 |
| 74 | CYP1B1 | 8051583 | -4.9617884 | 7.58660306 | -22.879055 | 6.07E-16 | 1.03E-13 | 26.7109405 |
| 75 | NA | 7895954 | -4.9554128 | 7.01303913 | -20.849747 | 3.70E-15 | 4.72E-13 | 24.8878092 |
| 76 | LPAR1 | 8163257 | -4.9336761 | 8.32011908 | -32.517217 | 5.91E-19 | 3.90E-16 | 33.5675178 |
| 77 | IFIT1 | 7929065 | -4.9286947 | 7.12628981 | -15.12802 | 1.68E-12 | 8.30E-11 | 18.6525282 |
| 78 | MME | 8083494 | -4.9262088 | 9.40747933 | -30.874258 | 1.66E-18 | 9.18E-16 | 32.5666893 |
| 79 | CDH11 | 8001800 | -4.925874 | 7.79198856 | -29.518059 | 4.03E-18 | 1.74E-15 | 31.6955299 |
| 80 | DDR2 | 7906878 | -4.9242529 | 8.03460771 | -35.169328 | 1.24E-19 | 1.21E-16 | 35.0695938 |
| 81 | GNG11 | 8134257 | -4.9228949 | 6.59210849 | -23.001106 | 5.47E-16 | 9.48E-14 | 26.8154028 |
| 82 | PDGFRA | 8095080 | -4.9073443 | 7.54952465 | -30.37237 | 2.29E-18 | 1.14E-15 | 32.2492435 |
| 83 | CPXM2 | 7936835 | -4.9032653 | 7.56601388 | -30.325733 | 2.36E-18 | 1.14E-15 | 32.2194554 |
| 84 | DDR2 | 7906900 | -4.898442 | 6.76865732 | -30.222395 | 2.53E-18 | 1.18E-15 | 32.1532725 |
| 85 | B2M | 7983360 | -4.8955188 | 9.80072117 | -30.426076 | 2.21E-18 | 1.14E-15 | 32.2834853 |
| 86 | NA | 7895417 | -4.868493 | 5.35182185 | -15.946231 | 6.25E-13 | 3.60E-11 | 19.6646689 |
| 87 | POSTN | 7971077 | -4.8631636 | 6.35870618 | -11.937848 | 1.27E-10 | 3.34E-09 | 14.2004406 |
| 88 | COX6A1 | 7959153 | -4.8608227 | 8.10895619 | -29.84195 | 3.25E-18 | 1.44E-15 | 31.9074781 |
| 89 | IGFBP4 | 8007100 | -4.8483458 | 10.083075 | -33.484019 | 3.30E-19 | 2.54E-16 | 34.1305559 |
| 90 | NA | 7894527 | -4.8300311 | 9.86982032 | -28.849731 | 6.35E-18 | 2.48E-15 | 31.2501441 |
| 91 | KIAA1199 | 7985317 | -4.8111225 | 8.21120367 | -22.560112 | 7.99E-16 | 1.26E-13 | 26.4352996 |
| 92 | MX1 | 8068713 | -4.8002916 | 7.51329356 | -12.38326 | 6.61E-11 | 1.95E-09 | 14.8762777 |
| 93 | NA | 8165707 | -4.779752 | 8.24372576 | -34.330558 | 2.01E-19 | 1.71E-16 | 34.608766 |
| 94 | NA | 8165709 | -4.7633609 | 6.16175139 | -36.974913 | 4.57E-20 | 5.91E-17 | 36.0202641 |
| 95 | FST | 8105302 | -4.7489483 | 7.83445592 | -25.444407 | 7.57E-17 | 1.93E-14 | 28.7960853 |
| 96 | EMP1 | 7954090 | -4.7382842 | 8.74329507 | -23.01008 | 5.43E-16 | 9.48E-14 | 26.8230615 |
| 97 | PSG4 | 8037283 | -4.7139188 | 8.22715173 | -25.812209 | 5.71E-17 | 1.55E-14 | 29.0772429 |
| 98 | NA | 7958197 | -4.7073929 | 8.41613841 | -30.325588 | 2.36E-18 | 1.14E-15 | 32.2193628 |
| 99 | STC2 | 8115851 | -4.6995562 | 7.69529584 | -22.99279 | 5.51E-16 | 9.48E-14 | 26.808303 |
| 100 | GREM2 | 7925452 | -4.6862976 | 7.93858104 | -28.778453 | 6.67E-18 | 2.50E-15 | 31.2019898 |
| 101 | NA | 7894698 | -4.661564 | 5.88998873 | -15.547925 | 1.00E-12 | 5.45E-11 | 19.1778267 |
| 102 | TFPI2 | 8141016 | -4.6600693 | 7.21906515 | -24.68769 | 1.37E-16 | 3.16E-14 | 28.2041897 |
| 103 | PAMR1 | 7947512 | -4.6528243 | 7.38775054 | -43.618811 | 1.67E-21 | 5.41E-18 | 39.0988179 |
| 104 | RNU5E-1 | 7897801 | -4.6457526 | 6.383738 | -23.211356 | 4.58E-16 | 8.27E-14 | 26.9940552 |
| 105 | FGF7 | 7983630 | -4.6193079 | 6.22187532 | -33.255989 | 3.78E-19 | 2.72E-16 | 33.9994123 |
| 106 | PTX3 | 8083594 | -4.6089838 | 7.34978344 | -12.569199 | 5.05E-11 | 1.57E-09 | 15.152758 |
| 107 | NA | 7893339 | -4.5946707 | 5.88283611 | -18.487439 | 3.76E-14 | 3.39E-12 | 22.5342166 |
| 108 | NA | 7895382 | -4.5774242 | 9.06901923 | -14.498257 | 3.70E-12 | 1.61E-10 | 17.840406 |
| 109 | TRIM22 | 7938035 | -4.5711501 | 6.26579291 | -21.107501 | 2.92E-15 | 3.88E-13 | 25.1288472 |
| 110 | NA | 7893564 | -4.549688 | 11.0029421 | -24.69356 | 1.36E-16 | 3.16E-14 | 28.2088525 |
| 111 | KCNJ2 | 8009502 | -4.5482199 | 7.66574654 | -20.940276 | 3.40E-15 | 4.44E-13 | 24.9727975 |
| 112 | VCAN | 8106743 | -4.5399144 | 7.07246183 | -21.156274 | 2.79E-15 | 3.76E-13 | 25.1741333 |
| 113 | PAPPA | 8157487 | -4.5363943 | 7.60640861 | -36.515874 | 5.86E-20 | 7.02E-17 | 35.7837287 |
| 114 | SERPINB2 | 8021635 | -4.5152171 | 7.15237924 | -19.446204 | 1.42E-14 | 1.48E-12 | 23.5223182 |
| 115 | CCRL1 | 8122334 | -4.4989943 | 6.51754715 | -24.546681 | 1.53E-16 | 3.49E-14 | 28.0918355 |
| 116 | CD248 | 7949588 | -4.4971821 | 7.88702979 | -19.796398 | 1.01E-14 | 1.14E-12 | 23.8717017 |
| 117 | EIF2AK2 | 8051501 | -4.4792422 | 7.02024766 | -16.323709 | 4.02E-13 | 2.49E-11 | 20.1161598 |
| 118 | CLMP | 7952341 | -4.459548 | 7.83561966 | -40.523344 | 7.31E-21 | 1.51E-17 | 37.7398866 |
| 119 | IFITM3 | 7945371 | -4.4580656 | 7.53811795 | -34.428342 | 1.90E-19 | 1.66E-16 | 34.6631442 |
| 120 | FTH1 | 7948656 | -4.4543723 | 9.1040216 | -30.325322 | 2.36E-18 | 1.14E-15 | 32.2191928 |
| 121 | LUM | 7965403 | -4.4474192 | 5.18456239 | -16.653405 | 2.75E-13 | 1.81E-11 | 20.502888 |
| 122 | IL1R1 | 8043995 | -4.4423718 | 7.33122154 | -25.150916 | 9.50E-17 | 2.29E-14 | 28.5687002 |
| 123 | ATP8B1 | 8023497 | -4.4412353 | 7.81737993 | -15.913458 | 6.49E-13 | 3.72E-11 | 19.625021 |
| 124 | GALNT15 | 8078155 | -4.4282693 | 7.44765893 | -17.875411 | 7.16E-14 | 5.83E-12 | 21.8779209 |
| 125 | NA | 7896064 | -4.4252017 | 8.0385402 | -19.491311 | 1.36E-14 | 1.43E-12 | 23.5676567 |
| 126 | NA | 7894990 | -4.3850746 | 8.55144762 | -21.343937 | 2.35E-15 | 3.27E-13 | 25.3474281 |
| 127 | PARP14 | 8082100 | -4.3822052 | 6.4554499 | -14.913574 | 2.19E-12 | 1.03E-10 | 18.3793251 |
| 128 | AK5 | 7902452 | -4.3620459 | 6.1632581 | -23.871553 | 2.65E-16 | 5.31E-14 | 27.544601 |
| 129 | TMTC1 | 7962058 | -4.3554226 | 7.12952908 | -27.299967 | 1.89E-17 | 6.05E-15 | 30.1734074 |
| 130 | SERPINF1 | 8003667 | -4.3494826 | 7.75679044 | -27.843983 | 1.28E-17 | 4.34E-15 | 30.5586173 |
| 131 | NA | 7896529 | -4.3402016 | 7.26621434 | -22.566262 | 7.95E-16 | 1.26E-13 | 26.440652 |
| 132 | CFH | 7908459 | -4.3390122 | 6.53899268 | -34.720897 | 1.60E-19 | 1.44E-16 | 34.8247962 |
| 133 | KYNU | 8045539 | -4.3263747 | 7.06057072 | -22.606221 | 7.68E-16 | 1.23E-13 | 26.4753887 |
| 134 | STC1 | 8149825 | -4.2905343 | 8.18063511 | -12.548731 | 5.20E-11 | 1.61E-09 | 15.1224837 |
| 135 | NRP1 | 7932985 | -4.2817519 | 7.84612608 | -35.72359 | 9.08E-20 | 9.47E-17 | 35.3672848 |
| 136 | SNORD57 | 8060503 | -4.2783523 | 7.90722915 | -19.226733 | 1.77E-14 | 1.79E-12 | 23.3002763 |
| 137 | SLIRP | 7975989 | -4.2724967 | 7.75603767 | -22.587826 | 7.80E-16 | 1.24E-13 | 26.459405 |
| 138 | FBN2 | 8113800 | -4.2600987 | 7.70320354 | -19.470551 | 1.39E-14 | 1.45E-12 | 23.5468021 |
| 139 | CXCL1 | 8095697 | -4.2181447 | 9.00151681 | -12.737285 | 3.98E-11 | 1.28E-09 | 15.3999032 |
| 140 | NA | 7896169 | -4.2108507 | 8.69518723 | -29.145237 | 5.19E-18 | 2.15E-15 | 31.4484302 |
| 141 | PRNP | 8060758 | -4.1940768 | 8.58063238 | -38.502949 | 2.03E-20 | 2.99E-17 | 36.7836564 |
| 142 | SNORD41 | 8034512 | -4.1937484 | 6.376897 | -33.648253 | 2.99E-19 | 2.36E-16 | 34.2243896 |
| 143 | NA | 7896370 | -4.1659318 | 8.55222192 | -15.365697 | 1.25E-12 | 6.55E-11 | 18.9514074 |
| 144 | NA | 7895252 | -4.1423448 | 5.46688103 | -15.461547 | 1.12E-12 | 5.91E-11 | 19.0707939 |
| 145 | SNORD3A | 8013329 | -4.1340761 | 8.02326462 | -24.275058 | 1.91E-16 | 4.13E-14 | 27.873535 |
| 146 | NA | 7893497 | -4.1255638 | 5.1483035 | -16.068515 | 5.41E-13 | 3.19E-11 | 19.8119663 |
| 147 | NID2 | 7979133 | -4.1176407 | 7.0947942 | -23.793246 | 2.82E-16 | 5.56E-14 | 27.4801114 |
| 148 | COL3A1 | 8046922 | -4.1159338 | 8.06118229 | -15.548919 | 1.00E-12 | 5.45E-11 | 19.1790544 |
| 149 | NA | 7892610 | -4.1067819 | 4.11737481 | -18.011814 | 6.19E-14 | 5.25E-12 | 22.0259855 |
| 150 | RECK | 8155169 | -4.1050886 | 8.73603306 | -30.817188 | 1.72E-18 | 9.25E-16 | 32.5308777 |
| 151 | NA | 8165694 | -4.1045126 | 7.67102497 | -24.747929 | 1.31E-16 | 3.08E-14 | 28.2519868 |
| 152 | RGMB | 8107100 | -4.0938995 | 8.17757124 | -40.476079 | 7.49E-21 | 1.51E-17 | 37.7181713 |
| 153 | PLAU | 7928429 | -4.0858752 | 8.67196958 | -29.991978 | 2.94E-18 | 1.34E-15 | 32.0048107 |
| 154 | NA | 7896095 | -4.0700221 | 4.97849437 | -17.424063 | 1.17E-13 | 8.95E-12 | 21.3804045 |
| 155 | NA | 7892598 | -4.0690577 | 6.87641379 | -14.511002 | 3.64E-12 | 1.59E-10 | 17.8571406 |
| 156 | KITLG | 7965322 | -4.0553336 | 5.84182456 | -18.674599 | 3.10E-14 | 2.86E-12 | 22.7308514 |
| 157 | HIGD1A | 8086451 | -4.0458808 | 5.74306115 | -24.096633 | 2.20E-16 | 4.59E-14 | 27.7287762 |
| 158 | IL6ST | 8112139 | -4.0300016 | 8.80715053 | -21.063643 | 3.04E-15 | 4.02E-13 | 25.0880373 |
| 159 | COL12A1 | 8127563 | -4.0174079 | 8.83210675 | -19.25193 | 1.73E-14 | 1.75E-12 | 23.325891 |
| 160 | CLDN11 | 8083887 | -4.0136898 | 8.89415942 | -23.585625 | 3.35E-16 | 6.49E-14 | 27.3080768 |
| 161 | NBPF15 | 7919412 | -4.0065628 | 6.96475379 | -23.901373 | 2.58E-16 | 5.25E-14 | 27.5691026 |
| 162 | TNFAIP6 | 8045688 | -3.9986373 | 6.09119397 | -24.31494 | 1.85E-16 | 4.03E-14 | 27.9057435 |
| 163 | NA | 7895283 | -3.9858081 | 6.13347641 | -12.977397 | 2.83E-11 | 9.52E-10 | 15.7484449 |
| 164 | GUSBP1 | 8104621 | -3.9744051 | 5.18291605 | -13.559897 | 1.27E-11 | 4.72E-10 | 16.572658 |
| 165 | FGF5 | 8096050 | -3.9570552 | 7.56452256 | -19.280082 | 1.68E-14 | 1.72E-12 | 23.3544719 |
| 166 | MX2 | 8068697 | -3.9565471 | 6.47126203 | -12.621366 | 4.69E-11 | 1.48E-09 | 15.2297424 |
| 167 | NBPF16 | 7904574 | -3.9326708 | 6.78159164 | -38.701951 | 1.84E-20 | 2.82E-17 | 36.8804558 |
| 168 | IFI6 | 7914127 | -3.9311385 | 9.65771559 | -26.852679 | 2.62E-17 | 7.84E-15 | 29.8505677 |
| 169 | CPED1 | 8135734 | -3.9208487 | 6.52214346 | -31.839164 | 8.99E-19 | 5.69E-16 | 33.1613969 |
| 170 | ZBTB38 | 8083092 | -3.9178784 | 7.49453986 | -15.652739 | 8.86E-13 | 4.89E-11 | 19.3070007 |
| 171 | NA | 7896269 | -3.9133289 | 5.18073554 | -22.761994 | 6.71E-16 | 1.11E-13 | 26.6102215 |
| 172 | EIF5AL1 | 7928600 | -3.8897572 | 7.04718418 | -14.940598 | 2.12E-12 | 1.02E-10 | 18.4139413 |
| 173 | FAM21B | 7927323 | -3.8759533 | 7.23238089 | -17.96084 | 6.54E-14 | 5.43E-12 | 21.9707757 |
| 174 | CDR1 | 8175531 | -3.8752061 | 5.30462264 | -21.354005 | 2.33E-15 | 3.27E-13 | 25.3566832 |
| 175 | BDKRB1 | 7976567 | -3.866821 | 7.27644287 | -22.9512 | 5.71E-16 | 9.77E-14 | 26.7727558 |
| 176 | NA | 8107204 | -3.8531187 | 7.80708484 | -25.42137 | 7.70E-17 | 1.94E-14 | 28.7783357 |
| 177 | MPLKIP | 8137008 | -3.8526701 | 6.33874453 | -15.217817 | 1.50E-12 | 7.57E-11 | 18.7659277 |
| 178 | PHLDA1 | 7965040 | -3.8506169 | 8.41755158 | -27.433682 | 1.72E-17 | 5.61E-15 | 30.2688374 |
| 179 | FBLN5 | 7980908 | -3.8502102 | 9.14367219 | -34.985312 | 1.38E-19 | 1.27E-16 | 34.9695726 |
| 180 | PTGR1 | 8163328 | -3.8221283 | 6.27095751 | -28.781507 | 6.65E-18 | 2.50E-15 | 31.2040554 |
| 181 | DDX60 | 8103563 | -3.8137866 | 4.84165504 | -12.685923 | 4.28E-11 | 1.36E-09 | 15.324662 |
| 182 | CD99 | 8176360 | -3.8135866 | 9.30599483 | -35.978564 | 7.88E-20 | 8.49E-17 | 35.5024563 |
| 183 | IFIT3 | 7929052 | -3.8033521 | 6.60241928 | -10.706135 | 8.66E-10 | 1.72E-08 | 12.225943 |
| 184 | RXFP1 | 8098060 | -3.7936964 | 5.67955576 | -14.694169 | 2.88E-12 | 1.31E-10 | 18.0962575 |
| 185 | RSAD2 | 8040080 | -3.7840596 | 6.13366211 | -11.358128 | 3.08E-10 | 7.06E-09 | 13.2910737 |
| 186 | OLFML1 | 7938225 | -3.7794338 | 5.36522042 | -18.242611 | 4.86E-14 | 4.23E-12 | 22.2741521 |
| 187 | IFI44L | 7902541 | -3.7742299 | 6.29078907 | -12.434531 | 6.14E-11 | 1.84E-09 | 14.9528406 |
| 188 | ITGBL1 | 7969861 | -3.7612497 | 7.70712238 | -23.04834 | 5.26E-16 | 9.24E-14 | 26.8556811 |
| 189 | DSEL | 8023727 | -3.7553429 | 7.49099817 | -18.887828 | 2.49E-14 | 2.39E-12 | 22.9526237 |
| 190 | NA | 7896310 | -3.7379549 | 7.95334935 | -21.052503 | 3.07E-15 | 4.05E-13 | 25.0776586 |
| 191 | CCBE1 | 8023575 | -3.7251746 | 7.33731725 | -20.451434 | 5.38E-15 | 6.44E-13 | 24.5095532 |
| 192 | NA | 7895275 | -3.7155827 | 8.72553524 | -18.95172 | 2.34E-14 | 2.27E-12 | 23.0186151 |
| 193 | LTBP1 | 8041383 | -3.7151631 | 8.89551774 | -18.924781 | 2.40E-14 | 2.31E-12 | 22.9908166 |
| 194 | NA | 7894661 | -3.7106109 | 9.54557942 | -14.457853 | 3.90E-12 | 1.68E-10 | 17.7872717 |
| 195 | NA | 7892817 | -3.7103515 | 10.1930184 | -10.589984 | 1.05E-09 | 2.00E-08 | 12.0312834 |
| 196 | RPLP1 | 7984562 | -3.705807 | 8.01081353 | -12.410393 | 6.36E-11 | 1.90E-09 | 14.9168265 |
| 197 | NA | 7894725 | -3.6897655 | 9.6789326 | -23.555881 | 3.43E-16 | 6.61E-14 | 27.2833062 |
| 198 | MMP14 | 7973336 | -3.6883887 | 8.99790337 | -20.667821 | 4.39E-15 | 5.45E-13 | 24.7159238 |
| 199 | PPAP2B | 7916493 | -3.679179 | 9.1647922 | -29.465959 | 4.18E-18 | 1.78E-15 | 31.6612022 |
| 200 | NA | 8165680 | -3.6789384 | 7.11469383 | -9.5525841 | 6.05E-09 | 8.97E-08 | 10.2232654 |
| 201 | DTX3L | 8082075 | -3.675315 | 6.5818874 | -10.438358 | 1.34E-09 | 2.46E-08 | 11.7748742 |
| 202 | SNORD33 | 8030362 | -3.6514298 | 8.93805239 | -26.287427 | 3.98E-17 | 1.12E-14 | 29.4344106 |
| 203 | FAM180A | 8143127 | -3.6483928 | 7.09708812 | -20.362841 | 5.85E-15 | 6.93E-13 | 24.4244488 |
| 204 | STEAP1B | 8138527 | -3.6239359 | 5.94852659 | -14.479585 | 3.79E-12 | 1.65E-10 | 17.8158675 |
| 205 | CTSB | 8149330 | -3.6236993 | 10.6967668 | -30.241634 | 2.50E-18 | 1.18E-15 | 32.1656126 |
| 206 | TMEM176A | 8137264 | -3.6141358 | 7.14618186 | -23.126503 | 4.92E-16 | 8.74E-14 | 26.9221513 |
| 207 | S100A4 | 7920271 | -3.5969509 | 8.54903177 | -25.533638 | 7.06E-17 | 1.84E-14 | 28.8646813 |
| 208 | NQO1 | 8002303 | -3.5955782 | 7.86364511 | -14.925878 | 2.16E-12 | 1.02E-10 | 18.3950928 |
| 209 | NIPAL2 | 8151952 | -3.5931132 | 5.94819733 | -22.831121 | 6.33E-16 | 1.06E-13 | 26.6697606 |
| 210 | DKK3 | 7946661 | -3.5881474 | 8.38209016 | -22.670489 | 7.26E-16 | 1.18E-13 | 26.5311301 |
| 211 | CD46 | 7909400 | -3.5846733 | 7.24782363 | -26.951104 | 2.44E-17 | 7.45E-15 | 29.9220912 |
| 212 | AKR1C1 | 8180376 | -3.5781128 | 6.83508783 | -16.548842 | 3.10E-13 | 2.01E-11 | 20.3809906 |
| 213 | NBPF16 | 7904874 | -3.5693587 | 6.39832752 | -15.491623 | 1.08E-12 | 5.73E-11 | 19.1081214 |
| 214 | WIPI1 | 8017850 | -3.5655221 | 8.2519007 | -22.639654 | 7.46E-16 | 1.20E-13 | 26.5044056 |
| 215 | ROBO1 | 8088919 | -3.5632132 | 7.18924435 | -27.0396 | 2.28E-17 | 7.17E-15 | 29.9861657 |
| 216 | C1R | 7960744 | -3.546873 | 8.73460913 | -20.420738 | 5.54E-15 | 6.60E-13 | 24.4801064 |
| 217 | NA | 8165667 | -3.5441805 | 5.90400167 | -8.8497111 | 2.14E-08 | 2.66E-07 | 8.92375575 |
| 218 | NDUFS5 | 7900228 | -3.5422174 | 8.03488543 | -28.808578 | 6.53E-18 | 2.50E-15 | 31.2223575 |
| 219 | PTGS1 | 8157650 | -3.5379824 | 7.05243351 | -15.34175 | 1.29E-12 | 6.70E-11 | 18.9214785 |
| 220 | DDX60L | 8103601 | -3.528771 | 5.32109411 | -18.209822 | 5.03E-14 | 4.35E-12 | 22.239075 |
| 221 | SOD2 | 8130556 | -3.5259266 | 9.00081961 | -19.010926 | 2.20E-14 | 2.16E-12 | 23.0795786 |
| 222 | CTBS | 7917240 | -3.5200388 | 5.79642757 | -24.515243 | 1.57E-16 | 3.53E-14 | 28.0666956 |
| 223 | IGFBP5 | 8058857 | -3.5182835 | 6.90212472 | -12.122261 | 9.69E-11 | 2.64E-09 | 14.4826058 |
| 224 | MYH9 | 8075728 | -3.5049499 | 8.65835617 | -33.068222 | 4.23E-19 | 2.91E-16 | 33.890665 |
| 225 | PARP9 | 8090018 | -3.504046 | 6.25248888 | -9.2488634 | 1.04E-08 | 1.43E-07 | 9.66935562 |
| 226 | ACTA2 | 7934906 | -3.5005613 | 7.52852497 | -23.848681 | 2.70E-16 | 5.35E-14 | 27.5257871 |
| 227 | NA | 7893608 | -3.4920593 | 4.99309441 | -12.311021 | 7.34E-11 | 2.11E-09 | 14.7679744 |
| 228 | AHR | 8131614 | -3.4905829 | 7.8833533 | -31.753894 | 9.48E-19 | 5.89E-16 | 33.1096468 |
| 229 | KGFLP1 | 8067839 | -3.4800158 | 5.38485251 | -16.191355 | 4.69E-13 | 2.80E-11 | 19.9589286 |
| 230 | GBP1 | 7917516 | -3.4742346 | 6.65534299 | -19.594148 | 1.23E-14 | 1.31E-12 | 23.6706481 |
| 231 | NA | 7893958 | -3.4713525 | 4.87703905 | -13.985556 | 7.20E-12 | 2.87E-10 | 17.1566079 |
| 232 | AEBP1 | 8132557 | -3.4662413 | 8.29191677 | -22.684948 | 7.17E-16 | 1.17E-13 | 26.5436485 |
| 233 | CD302 | 8056102 | -3.4584021 | 5.35166278 | -18.933206 | 2.38E-14 | 2.30E-12 | 22.9995147 |
| 234 | TIMMDC1 | 8081867 | -3.4558379 | 6.61657059 | -17.611643 | 9.51E-14 | 7.48E-12 | 21.5886023 |
| 235 | NA | 8165696 | -3.453822 | 9.74711003 | -18.189834 | 5.13E-14 | 4.44E-12 | 22.217663 |
| 236 | CASP1 | 7951397 | -3.448347 | 4.84875481 | -18.121552 | 5.52E-14 | 4.74E-12 | 22.1443508 |
| 237 | SAMD9 | 8140967 | -3.4463372 | 5.90281117 | -7.8431416 | 1.45E-07 | 1.41E-06 | 6.95176404 |
| 238 | F8A1 | 8170998 | -3.4458905 | 5.53968633 | -7.4644864 | 3.09E-07 | 2.70E-06 | 6.17527504 |
| 239 | CYBRD1 | 8046333 | -3.4431446 | 9.53813834 | -26.878544 | 2.57E-17 | 7.76E-15 | 29.8693899 |
| 240 | NA | 7895752 | -3.441322 | 5.89074294 | -13.326313 | 1.75E-11 | 6.25E-10 | 16.2457037 |
| 241 | XG | 8165808 | -3.4411092 | 7.94017787 | -19.742445 | 1.06E-14 | 1.18E-12 | 23.8182609 |
| 242 | TMEM167A | 8112967 | -3.4242861 | 6.84332494 | -16.732745 | 2.52E-13 | 1.71E-11 | 20.5949184 |
| 243 | BCAT1 | 7961829 | -3.4196006 | 6.62739018 | -18.648239 | 3.19E-14 | 2.93E-12 | 22.7032692 |
| 244 | LAMA4 | 8128991 | -3.4151652 | 7.85177372 | -21.410244 | 2.21E-15 | 3.15E-13 | 25.4083015 |
| 245 | UIMC1 | 8165703 | -3.4061503 | 8.01962334 | -18.239113 | 4.87E-14 | 4.23E-12 | 22.2704126 |
| 246 | OAS2 | 7958913 | -3.4047027 | 6.22492022 | -11.858452 | 1.44E-10 | 3.68E-09 | 14.0779193 |
| 247 | PLA2G4A | 7908351 | -3.404179 | 6.35002357 | -20.366942 | 5.83E-15 | 6.92E-13 | 24.4283957 |
| 248 | CAV1 | 8135594 | -3.387323 | 9.38502375 | -12.159809 | 9.17E-11 | 2.51E-09 | 14.5396472 |
| 249 | IFI44 | 7902553 | -3.3779299 | 5.54134028 | -10.929566 | 6.05E-10 | 1.27E-08 | 12.5961666 |
| 250 | PSD3 | 8149551 | -3.3733687 | 6.46574097 | -16.600674 | 2.92E-13 | 1.90E-11 | 20.4415026 |
| 251 | SQSTM1 | 8110569 | -3.3651078 | 8.52017266 | -25.21356 | 9.05E-17 | 2.23E-14 | 28.6174632 |
| 252 | NA | 8112914 | -3.3649005 | 5.26660394 | -16.225804 | 4.50E-13 | 2.73E-11 | 19.999963 |
| 253 | NA | 7894896 | -3.3610327 | 8.72913361 | -9.6355121 | 5.23E-09 | 7.90E-08 | 10.3725234 |
| 254 | COPZ2 | 8016390 | -3.358172 | 7.89732471 | -15.244657 | 1.45E-12 | 7.38E-11 | 18.7997095 |
| 255 | NA | 7895276 | -3.3559297 | 10.021538 | -23.887756 | 2.61E-16 | 5.28E-14 | 27.5579184 |
| 256 | NA | 8059376 | -3.3376667 | 9.48789602 | -24.138457 | 2.13E-16 | 4.50E-14 | 27.7628064 |
| 257 | EPAS1 | 8041781 | -3.3332371 | 9.0197568 | -30.734605 | 1.81E-18 | 9.44E-16 | 32.4789281 |
| 258 | GRIA3 | 8169717 | -3.3226109 | 5.52702131 | -19.596998 | 1.23E-14 | 1.31E-12 | 23.6734958 |
| 259 | NA | 8165658 | -3.3185353 | 6.83648419 | -11.382934 | 2.97E-10 | 6.84E-09 | 13.3306957 |
| 260 | SNORD13 | 8086752 | -3.3160017 | 5.50499297 | -12.331896 | 7.12E-11 | 2.06E-09 | 14.7993225 |
| 261 | TBX3 | 7966690 | -3.3093892 | 7.46091018 | -25.336381 | 8.23E-17 | 2.06E-14 | 28.7127076 |
| 262 | RNU5D-1 | 7915592 | -3.3031322 | 3.77438703 | -11.947796 | 1.26E-10 | 3.30E-09 | 14.2157477 |
| 263 | RPS25 | 7952129 | -3.2990528 | 9.51093862 | -21.194093 | 2.69E-15 | 3.67E-13 | 25.2091773 |
| 264 | EEF1A1 | 8127544 | -3.291742 | 6.1589312 | -15.265192 | 1.42E-12 | 7.25E-11 | 18.8255193 |
| 265 | SBDS | 8139891 | -3.2893443 | 7.23942137 | -16.722974 | 2.54E-13 | 1.72E-11 | 20.5836057 |
| 266 | SYT11 | 7906061 | -3.2852097 | 6.95643146 | -33.804159 | 2.73E-19 | 2.21E-16 | 34.3129895 |
| 267 | MGC72080 | 8045887 | -3.2807259 | 5.2246317 | -12.758346 | 3.86E-11 | 1.25E-09 | 15.4306863 |
| 268 | ALCAM | 8081431 | -3.2778438 | 7.08402951 | -24.168207 | 2.08E-16 | 4.45E-14 | 27.786976 |
| 269 | RND3 | 8055688 | -3.2588539 | 10.2366313 | -20.828013 | 3.78E-15 | 4.77E-13 | 24.867352 |
| 270 | NA | 7893371 | -3.2574808 | 10.7758673 | -22.048883 | 1.25E-15 | 1.89E-13 | 25.9852624 |
| 271 | ASAH1 | 8149534 | -3.2506924 | 7.95975734 | -39.676168 | 1.12E-20 | 2.12E-17 | 37.3459883 |
| 272 | NDUFC2 | 7950644 | -3.2487364 | 6.57337812 | -13.665909 | 1.10E-11 | 4.14E-10 | 16.7195082 |
| 273 | LTBP2 | 7980152 | -3.2403386 | 7.93283486 | -29.776175 | 3.40E-18 | 1.48E-15 | 31.8646387 |
| 274 | NA | 7894374 | -3.2297801 | 8.53186665 | -15.988298 | 5.94E-13 | 3.46E-11 | 19.7154549 |
| 275 | CDH6 | 8104663 | -3.2239774 | 7.30472445 | -14.078331 | 6.37E-12 | 2.59E-10 | 17.2819003 |
| 276 | IRAK3 | 7956878 | -3.2238437 | 6.22606287 | -14.354329 | 4.45E-12 | 1.89E-10 | 17.6505503 |
| 277 | NA | 7946567 | -3.2220085 | 6.8445829 | -14.831809 | 2.43E-12 | 1.13E-10 | 18.2742587 |
| 278 | NA | 7894666 | -3.217423 | 9.54243191 | -11.760284 | 1.66E-10 | 4.18E-09 | 13.9255532 |
| 279 | NA | 7910385 | -3.216299 | 9.15458577 | -15.821987 | 7.24E-13 | 4.10E-11 | 19.5139761 |
| 280 | HERC6 | 8096335 | -3.2112885 | 6.14315469 | -9.2032032 | 1.12E-08 | 1.54E-07 | 9.58508721 |
| 281 | KCNK2 | 7909730 | -3.1985434 | 6.55388804 | -10.788181 | 7.59E-10 | 1.54E-08 | 12.362535 |
| 282 | CABLES1 | 8020495 | -3.1980102 | 7.59459854 | -19.377793 | 1.52E-14 | 1.57E-12 | 23.4533628 |
| 283 | TNC | 8163637 | -3.1921256 | 7.04098344 | -14.68183 | 2.93E-12 | 1.33E-10 | 18.0802302 |
| 284 | FAT4 | 8097288 | -3.1844811 | 6.60508607 | -16.015054 | 5.76E-13 | 3.36E-11 | 19.7476936 |
| 285 | EDIL3 | 8112980 | -3.1825105 | 6.12087749 | -17.183183 | 1.52E-13 | 1.11E-11 | 21.1100019 |
| 286 | APOL1 | 8072735 | -3.1747947 | 6.93317409 | -21.290568 | 2.47E-15 | 3.41E-13 | 25.2982978 |
| 287 | KRTAP1-5 | 8019588 | -3.1731631 | 8.30474656 | -10.491068 | 1.23E-09 | 2.28E-08 | 11.8643058 |
| 288 | GUSBP1 | 8111455 | -3.1657058 | 4.94399688 | -12.352874 | 6.91E-11 | 2.02E-09 | 14.8307828 |
| 289 | PCOLCE | 8134869 | -3.1593596 | 8.7591597 | -24.977075 | 1.09E-16 | 2.61E-14 | 28.4327208 |
| 290 | SGCD | 8109490 | -3.1545073 | 6.22002797 | -15.976302 | 6.03E-13 | 3.50E-11 | 19.7009846 |
| 291 | NA | 7911341 | -3.1497546 | 4.26477085 | -6.6037549 | 1.85E-06 | 1.30E-05 | 4.34035396 |
| 292 | PDCD1LG2 | 8154245 | -3.1458182 | 6.09245438 | -11.717417 | 1.78E-10 | 4.41E-09 | 13.8587142 |
| 293 | NA | 7894068 | -3.1429944 | 8.04805625 | -17.690284 | 8.74E-14 | 6.96E-12 | 21.6752792 |
| 294 | SLFN5 | 8006531 | -3.1256695 | 8.25580973 | -18.470045 | 3.83E-14 | 3.43E-12 | 22.515847 |
| 295 | OGFRL1 | 8120602 | -3.1240417 | 6.71072789 | -23.148806 | 4.83E-16 | 8.67E-14 | 26.941076 |
| 296 | MAP1A | 7983228 | -3.1149381 | 7.29507892 | -20.455267 | 5.36E-15 | 6.44E-13 | 24.5132267 |
| 297 | ITGA4 | 8046695 | -3.1104459 | 7.16084604 | -23.133054 | 4.89E-16 | 8.74E-14 | 26.9277114 |
| 298 | CASP4 | 7951372 | -3.1041124 | 5.99445655 | -21.684581 | 1.73E-15 | 2.52E-13 | 25.6581985 |
| 299 | NA | 7895677 | -3.1006608 | 5.07957851 | -9.9624051 | 2.98E-09 | 4.89E-08 | 10.9527465 |
| 300 | FGF2 | 8097256 | -3.0983967 | 8.39587601 | -16.866855 | 2.16E-13 | 1.51E-11 | 20.7495831 |
| 301 | COL1A1 | 8016646 | -3.0935968 | 11.0213943 | -11.949252 | 1.25E-10 | 3.30E-09 | 14.2179884 |
| 302 | PTGFR | 7902527 | -3.0924279 | 6.0648592 | -19.723981 | 1.08E-14 | 1.19E-12 | 23.7999403 |
| 303 | EDNRB | 7972157 | -3.0903716 | 5.79466931 | -6.6502893 | 1.68E-06 | 1.19E-05 | 4.4419739 |
| 304 | IL7R | 8104901 | -3.085853 | 5.58013429 | -7.5497063 | 2.60E-07 | 2.33E-06 | 6.35168902 |
| 305 | SAMHD1 | 8066117 | -3.0832576 | 7.62933974 | -9.7040581 | 4.65E-09 | 7.11E-08 | 10.4952614 |
| 306 | NA | 7896044 | -3.0806524 | 9.10506331 | -16.851245 | 2.20E-13 | 1.53E-11 | 20.7316385 |
| 307 | NA | 7895729 | -3.0778015 | 5.76237204 | -9.8971594 | 3.33E-09 | 5.36E-08 | 10.8379654 |
| 308 | NA | 7895277 | -3.0767923 | 9.63478719 | -11.953189 | 1.25E-10 | 3.28E-09 | 14.224043 |
| 309 | ERAP2 | 8107044 | -3.0754499 | 6.46549874 | -17.972267 | 6.46E-14 | 5.38E-12 | 21.9831651 |
| 310 | CTSS | 7919800 | -3.0711921 | 5.96767374 | -17.260096 | 1.40E-13 | 1.04E-11 | 21.196718 |
| 311 | IFIT2 | 7929047 | -3.0540062 | 6.4806663 | -8.2014907 | 7.23E-08 | 7.67E-07 | 7.66909302 |
| 312 | MKX | 7932733 | -3.0519487 | 7.19263336 | -20.775522 | 3.97E-15 | 4.99E-13 | 24.8178579 |
| 313 | QPCT | 8041508 | -3.0513671 | 5.85602135 | -19.888653 | 9.22E-15 | 1.05E-12 | 23.9627563 |
| 314 | FADS2 | 7940565 | -3.0492988 | 7.2827714 | -22.238064 | 1.06E-15 | 1.63E-13 | 26.1529987 |
| 315 | GAS6 | 7970329 | -3.0457532 | 8.30780155 | -21.606358 | 1.85E-15 | 2.66E-13 | 25.5872631 |
| 316 | NA | 7894722 | -3.0403674 | 7.73045921 | -12.386091 | 6.58E-11 | 1.95E-09 | 14.8805109 |
| 317 | NBPF16 | 7919438 | -3.0348849 | 8.59446307 | -23.245472 | 4.45E-16 | 8.13E-14 | 27.0228897 |
| 318 | LPXN | 7948332 | -3.0295527 | 6.11049814 | -10.915675 | 6.18E-10 | 1.29E-08 | 12.5733098 |
| 319 | HGF | 8140556 | -3.0265163 | 5.3713942 | -14.519068 | 3.60E-12 | 1.58E-10 | 17.8677244 |
| 320 | NEXN | 7902495 | -3.024778 | 5.73857282 | -10.195412 | 2.01E-09 | 3.48E-08 | 11.3585329 |
| 321 | LOC388022 | 7977270 | -3.0223159 | 8.30158206 | -10.633514 | 9.75E-10 | 1.90E-08 | 12.1044128 |
| 322 | ABI3BP | 8089145 | -3.0190232 | 6.51661491 | -13.325763 | 1.75E-11 | 6.25E-10 | 16.2449289 |
| 323 | FLRT2 | 7976073 | -3.0139998 | 6.33599011 | -14.984605 | 2.00E-12 | 9.66E-11 | 18.470194 |
| 324 | NA | 7892565 | -3.0041077 | 6.44874443 | -13.824859 | 8.91E-12 | 3.43E-10 | 16.937921 |
| 325 | OAS3 | 7958895 | -2.9954506 | 6.90814679 | -10.900462 | 6.34E-10 | 1.32E-08 | 12.5482532 |
| 326 | SAMD9L | 8140971 | -2.9944849 | 5.81567071 | -8.6479055 | 3.10E-08 | 3.70E-07 | 8.53902231 |
| 327 | PANX1 | 7943218 | -2.9885518 | 7.91242413 | -28.856854 | 6.32E-18 | 2.48E-15 | 31.2549495 |
| 328 | RDH10 | 8146921 | -2.9876739 | 8.45218185 | -14.327656 | 4.61E-12 | 1.94E-10 | 17.6151876 |
| 329 | FIBIN | 7939052 | -2.9811275 | 6.14295559 | -13.149329 | 2.23E-11 | 7.72E-10 | 15.9948214 |
| 330 | STS | 8165866 | -2.978219 | 6.71052874 | -21.208687 | 2.66E-15 | 3.66E-13 | 25.2226847 |
| 331 | ANTXR2 | 8101260 | -2.9639094 | 8.04799145 | -18.241853 | 4.86E-14 | 4.23E-12 | 22.273342 |
| 332 | IL8 | 8095680 | -2.9626513 | 7.78297458 | -9.949442 | 3.05E-09 | 4.98E-08 | 10.9299822 |
| 333 | RFC1 | 8165672 | -2.9608027 | 3.49397007 | -7.0502688 | 7.22E-07 | 5.72E-06 | 5.30418333 |
| 334 | TPM2 | 8161044 | -2.9574663 | 9.01889019 | -22.477968 | 8.58E-16 | 1.35E-13 | 26.3636778 |
| 335 | PMS2P5 | 8140258 | -2.9561393 | 4.66207301 | -11.950409 | 1.25E-10 | 3.29E-09 | 14.2197666 |
| 336 | IL24 | 7909271 | -2.9557574 | 6.5605335 | -6.7499802 | 1.36E-06 | 9.93E-06 | 4.65876777 |
| 337 | SRPX2 | 8168749 | -2.9540861 | 7.44681953 | -21.163773 | 2.77E-15 | 3.75E-13 | 25.1810871 |
| 338 | PDGFRB | 8115099 | -2.9524947 | 7.87372317 | -18.56227 | 3.48E-14 | 3.18E-12 | 22.6130589 |
| 339 | NA | 7895001 | -2.9515394 | 8.06474099 | -11.794936 | 1.58E-10 | 4.01E-09 | 13.9794475 |
| 340 | CHCHD2 | 8139758 | -2.9498828 | 7.38233268 | -24.04239 | 2.30E-16 | 4.77E-14 | 27.6845517 |
| 341 | TFAP2C | 8063536 | -2.9480078 | 7.62839979 | -26.46795 | 3.48E-17 | 1.00E-14 | 29.5683275 |
| 342 | SLC16A7 | 7956658 | -2.9462264 | 6.71564785 | -26.94671 | 2.44E-17 | 7.45E-15 | 29.918904 |
| 343 | PVRL3 | 8081548 | -2.946117 | 8.27291739 | -18.03146 | 6.07E-14 | 5.17E-12 | 22.0472257 |
| 344 | DAB2 | 8111772 | -2.9297442 | 8.5771622 | -14.465516 | 3.86E-12 | 1.67E-10 | 17.79736 |
| 345 | NA | 7893086 | -2.913609 | 7.21346685 | -16.834211 | 2.24E-13 | 1.56E-11 | 20.7120397 |
| 346 | SDCBP | 8146550 | -2.9124497 | 8.95215968 | -21.929869 | 1.39E-15 | 2.09E-13 | 25.8790058 |
| 347 | STAT2 | 7964119 | -2.9102723 | 7.8779055 | -18.487849 | 3.76E-14 | 3.39E-12 | 22.5346492 |
| 348 | LIF | 8075310 | -2.9069102 | 6.78190115 | -17.179223 | 1.53E-13 | 1.12E-11 | 21.1055277 |
| 349 | EMP3 | 8030007 | -2.9062366 | 8.25851332 | -16.094401 | 5.25E-13 | 3.11E-11 | 19.8430196 |
| 350 | HERC5 | 8096361 | -2.9058993 | 5.36467956 | -11.860764 | 1.43E-10 | 3.67E-09 | 14.0814967 |
| 351 | CXCL12 | 7933194 | -2.9025693 | 7.15159274 | -14.424427 | 4.07E-12 | 1.75E-10 | 17.7432186 |
| 352 | SLC7A14 | 8092055 | -2.9020946 | 6.43476861 | -16.220875 | 4.53E-13 | 2.73E-11 | 19.9940973 |
| 353 | VEGFC | 8103822 | -2.9019635 | 8.69979945 | -17.641188 | 9.21E-14 | 7.30E-12 | 21.6212076 |
| 354 | IFI16 | 7906400 | -2.8959191 | 5.49667135 | -16.769691 | 2.41E-13 | 1.65E-11 | 20.6376389 |
| 355 | LRP10 | 7973352 | -2.8847162 | 8.54805101 | -29.069563 | 5.46E-18 | 2.24E-15 | 31.397859 |
| 356 | GSTM3 | 7918379 | -2.8836228 | 5.30711174 | -20.294547 | 6.24E-15 | 7.36E-13 | 24.3585989 |
| 357 | COL6A2 | 8069301 | -2.8801952 | 9.84535398 | -20.844294 | 3.72E-15 | 4.72E-13 | 24.8826787 |
| 358 | EYA1 | 8151310 | -2.8793217 | 6.70440404 | -21.756574 | 1.62E-15 | 2.40E-13 | 25.7232613 |
| 359 | NA | 7895088 | -2.8773635 | 8.58892361 | -15.344602 | 1.29E-12 | 6.70E-11 | 18.9250454 |
| 360 | PRSS12 | 8102468 | -2.8717365 | 8.53158134 | -27.836116 | 1.29E-17 | 4.34E-15 | 30.5531035 |
| 361 | SPATA18 | 8095021 | -2.87009 | 6.6988565 | -23.536395 | 3.49E-16 | 6.68E-14 | 27.2670602 |
| 362 | FLT1 | 7970763 | -2.8693419 | 5.40836587 | -13.539927 | 1.31E-11 | 4.84E-10 | 16.5448875 |
| 363 | CCND1 | 7942123 | -2.860988 | 9.48523052 | -14.266242 | 4.99E-12 | 2.09E-10 | 17.5335518 |
| 364 | FBN1 | 7988467 | -2.8600507 | 8.58222126 | -17.553127 | 1.01E-13 | 7.93E-12 | 21.5238754 |
| 365 | CCL5 | 8014316 | -2.855032 | 6.69494439 | -14.370595 | 4.36E-12 | 1.86E-10 | 17.6720879 |
| 366 | NA | 7894074 | -2.8540878 | 9.95562758 | -23.851611 | 2.69E-16 | 5.35E-14 | 27.528198 |
| 367 | NA | 7894496 | -2.8519564 | 8.38618694 | -20.572507 | 4.80E-15 | 5.90E-13 | 24.6252818 |
| 368 | VGLL3 | 8088979 | -2.8518177 | 7.09434998 | -15.496744 | 1.07E-12 | 5.72E-11 | 19.1144701 |
| 369 | EFEMP2 | 7949503 | -2.849926 | 8.446497 | -23.220207 | 4.55E-16 | 8.26E-14 | 27.0015398 |
| 370 | CD59 | 7947425 | -2.8490048 | 8.65051482 | -21.683457 | 1.73E-15 | 2.52E-13 | 25.6571811 |
| 371 | TRIM16L | 8005475 | -2.8487517 | 6.76622743 | -16.72308 | 2.54E-13 | 1.72E-11 | 20.5837285 |
| 372 | RGCC | 7968789 | -2.8336725 | 6.89349775 | -22.189636 | 1.10E-15 | 1.69E-13 | 26.1101954 |
| 373 | IMPAD1 | 8150906 | -2.8315909 | 6.5921056 | -14.715307 | 2.81E-12 | 1.28E-10 | 18.1236871 |
| 374 | NA | 7895818 | -2.8267544 | 8.32042264 | -11.092525 | 4.67E-10 | 1.01E-08 | 12.8627308 |
| 375 | SEMA3A | 8140668 | -2.8265168 | 7.50422595 | -9.5427462 | 6.15E-09 | 9.12E-08 | 10.2055026 |
| 376 | CPQ | 8147469 | -2.8226368 | 6.65532199 | -24.45543 | 1.65E-16 | 3.67E-14 | 28.0187745 |
| 377 | C2 | 8118324 | -2.8196709 | 4.09487918 | -8.8584165 | 2.10E-08 | 2.63E-07 | 8.94023399 |
| 378 | PMS2L2 | 8140280 | -2.8158382 | 6.27309804 | -21.404722 | 2.22E-15 | 3.15E-13 | 25.4032386 |
| 379 | FTL | 8030171 | -2.8124977 | 9.90743707 | -13.028337 | 2.64E-11 | 8.95E-10 | 15.8217157 |
| 380 | SHC3 | 8162216 | -2.8114761 | 7.28037862 | -23.395813 | 3.92E-16 | 7.38E-14 | 27.1494536 |
| 381 | SNORD13 | 8145793 | -2.8108777 | 7.25561735 | -12.345341 | 6.99E-11 | 2.04E-09 | 14.8194902 |
| 382 | COL6A1 | 8069269 | -2.8091861 | 10.2144074 | -19.257479 | 1.72E-14 | 1.75E-12 | 23.331528 |
| 383 | UCHL1 | 8094778 | -2.8055705 | 7.79123516 | -17.81354 | 7.65E-14 | 6.17E-12 | 21.8104134 |
| 384 | COL8A1 | 8081235 | -2.802985 | 5.34876735 | -14.56625 | 3.39E-12 | 1.51E-10 | 17.9295367 |
| 385 | TCTN3 | 7935251 | -2.7866878 | 7.50783513 | -20.220814 | 6.70E-15 | 7.84E-13 | 24.2872625 |
| 386 | MAB21L2 | 8097773 | -2.7790882 | 6.62652248 | -19.036506 | 2.14E-14 | 2.12E-12 | 23.105862 |
| 387 | NA | 7895490 | -2.773136 | 6.02488364 | -7.296126 | 4.35E-07 | 3.65E-06 | 5.82393189 |
| 388 | TMEM47 | 8172022 | -2.7689549 | 9.50290279 | -16.700265 | 2.61E-13 | 1.74E-11 | 20.5572918 |
| 389 | SERPING1 | 7940028 | -2.7650065 | 7.00732388 | -21.66862 | 1.75E-15 | 2.54E-13 | 25.643745 |
| 390 | ZEB2 | 8055624 | -2.7572791 | 7.23743454 | -21.347802 | 2.34E-15 | 3.27E-13 | 25.3509814 |
| 391 | RPL13AP5 | 7929593 | -2.7449621 | 10.2556748 | -15.212124 | 1.51E-12 | 7.60E-11 | 18.7587555 |
| 392 | FAM111A | 7940153 | -2.7426178 | 6.45915759 | -14.394168 | 4.23E-12 | 1.81E-10 | 17.7032636 |
| 393 | NA | 7894447 | -2.7423686 | 4.37709412 | -10.300413 | 1.69E-09 | 2.99E-08 | 11.5393142 |
| 394 | MASP1 | 8092661 | -2.7373024 | 6.54602729 | -23.281439 | 4.32E-16 | 7.98E-14 | 27.0532431 |
| 395 | USP18 | 8074606 | -2.7347609 | 6.80262207 | -8.9257402 | 1.86E-08 | 2.37E-07 | 9.06734049 |
| 396 | FAM26E | 8121601 | -2.7242618 | 6.1175423 | -11.567083 | 2.23E-10 | 5.38E-09 | 13.622827 |
| 397 | CXCL6 | 8095688 | -2.7235572 | 7.04509134 | -16.626244 | 2.84E-13 | 1.85E-11 | 20.4712911 |
| 398 | NBPF15 | 7919271 | -2.7230556 | 5.9983718 | -7.3859838 | 3.62E-07 | 3.11E-06 | 6.01191671 |
| 399 | TAGLN | 7944082 | -2.7206753 | 7.74755907 | -11.923269 | 1.30E-10 | 3.41E-09 | 14.1779903 |
| 400 | LACC1 | 7968883 | -2.7084967 | 5.21251021 | -12.887877 | 3.21E-11 | 1.06E-09 | 15.619112 |
| 401 | FZD7 | 8047487 | -2.707138 | 7.77942025 | -25.264957 | 8.70E-17 | 2.16E-14 | 28.6573784 |
| 402 | MYL9 | 8062312 | -2.7023036 | 8.60614812 | -20.976118 | 3.29E-15 | 4.31E-13 | 25.0063462 |
| 403 | ITGA2 | 8105267 | -2.6977761 | 7.13892342 | -15.139964 | 1.65E-12 | 8.20E-11 | 18.6676455 |
| 404 | SRPX | 8172043 | -2.6951548 | 7.44600882 | -17.193734 | 1.50E-13 | 1.10E-11 | 21.1219185 |
| 405 | PSMB8 | 8180049 | -2.6936124 | 6.54723031 | -14.303548 | 4.75E-12 | 2.00E-10 | 17.5831762 |
| 406 | CSTB | 8070701 | -2.6929797 | 8.91596081 | -24.522552 | 1.56E-16 | 3.53E-14 | 28.0725434 |
| 407 | KIT | 8095110 | -2.6926429 | 5.63552874 | -9.7183083 | 4.53E-09 | 6.97E-08 | 10.5207058 |
| 408 | SVIL | 7932796 | -2.6914992 | 7.70704143 | -14.97828 | 2.02E-12 | 9.73E-11 | 18.4621183 |
| 409 | SVEP1 | 8163202 | -2.6891894 | 7.59569839 | -14.805908 | 2.51E-12 | 1.16E-10 | 18.2408714 |
| 410 | POPDC3 | 8128565 | -2.6868154 | 5.94608092 | -19.869786 | 9.40E-15 | 1.07E-12 | 23.944168 |
| 411 | IGFBP7 | 8100541 | -2.6848972 | 8.75146103 | -20.556663 | 4.87E-15 | 5.92E-13 | 24.6101746 |
| 412 | C16orf45 | 7993458 | -2.6761008 | 6.47971889 | -25.459511 | 7.48E-17 | 1.92E-14 | 28.8077143 |
| 413 | NPIP | 7999769 | -2.6760464 | 6.89339568 | -30.855109 | 1.68E-18 | 9.18E-16 | 32.5546818 |
| 414 | RMRP | 8161024 | -2.6756006 | 9.23874536 | -30.089657 | 2.76E-18 | 1.27E-15 | 32.0678981 |
| 415 | RNF213 | 8010426 | -2.6749492 | 7.58720526 | -16.403951 | 3.66E-13 | 2.30E-11 | 20.210926 |
| 416 | DFNA5 | 8138602 | -2.673479 | 6.65286307 | -16.541249 | 3.13E-13 | 2.01E-11 | 20.3721122 |
| 417 | OAS1 | 7958884 | -2.6683326 | 7.20954944 | -8.5144188 | 3.99E-08 | 4.59E-07 | 8.28163191 |
| 418 | MYLK | 8090098 | -2.6643915 | 7.85593268 | -19.356393 | 1.56E-14 | 1.60E-12 | 23.4317453 |
| 419 | AKR1C3 | 7925929 | -2.6624912 | 6.32790118 | -12.094414 | 1.01E-10 | 2.74E-09 | 14.4402124 |
| 420 | P4HB | 8019762 | -2.6608839 | 9.31921666 | -22.794939 | 6.53E-16 | 1.09E-13 | 26.6386194 |
| 421 | MAN1A1 | 8129254 | -2.6542006 | 7.79206997 | -21.69454 | 1.71E-15 | 2.51E-13 | 25.6672111 |
| 422 | LHX8 | 7902353 | -2.6530914 | 6.2465228 | -16.136864 | 4.99E-13 | 2.97E-11 | 19.8938615 |
| 423 | SLC38A2 | 7962537 | -2.6454953 | 10.053838 | -13.176602 | 2.15E-11 | 7.49E-10 | 16.0336613 |
| 424 | KGFLP2 | 8161362 | -2.6438712 | 5.45025524 | -15.895246 | 6.63E-13 | 3.79E-11 | 19.602957 |
| 425 | GPR176 | 7987439 | -2.6418431 | 7.91467252 | -25.521578 | 7.13E-17 | 1.84E-14 | 28.8554244 |
| 426 | HSPA1A | 8179322 | -2.6403922 | 7.76742567 | -11.401665 | 2.88E-10 | 6.69E-09 | 13.3605718 |
| 427 | CNIH3 | 7910022 | -2.6389215 | 6.62434488 | -15.587384 | 9.58E-13 | 5.24E-11 | 19.2265462 |
| 428 | DUSP6 | 7965335 | -2.6383062 | 6.87777027 | -14.64161 | 3.08E-12 | 1.38E-10 | 18.027908 |
| 429 | RPL13AP20 | 7954063 | -2.634131 | 7.57141026 | -22.704123 | 7.05E-16 | 1.16E-13 | 26.5602383 |
| 430 | PHF11 | 7969129 | -2.6303635 | 6.23325223 | -14.406642 | 4.16E-12 | 1.78E-10 | 17.7197437 |
| 431 | NBPF16 | 7904999 | -2.6242765 | 5.82726568 | -12.612506 | 4.75E-11 | 1.49E-09 | 15.2166867 |
| 432 | XAF1 | 8004184 | -2.6195513 | 6.62723582 | -8.7277108 | 2.68E-08 | 3.26E-07 | 8.69179718 |
| 433 | NA | 8108180 | -2.6191968 | 7.68881135 | -19.78231 | 1.02E-14 | 1.14E-12 | 23.8577608 |
| 434 | NA | 7893777 | -2.6169325 | 6.54640191 | -9.5039237 | 6.59E-09 | 9.63E-08 | 10.1352905 |
| 435 | FBLN1 | 8073775 | -2.6109331 | 9.64464075 | -21.106689 | 2.92E-15 | 3.88E-13 | 25.1280919 |
| 436 | ZBTB38 | 8083090 | -2.6100224 | 9.24032706 | -11.870222 | 1.41E-10 | 3.64E-09 | 14.0961233 |
| 437 | BMPER | 8132250 | -2.6097921 | 6.83736432 | -13.758856 | 9.73E-12 | 3.70E-10 | 16.8474827 |
| 438 | KIAA1191 | 8115895 | -2.6064408 | 6.96576845 | -16.138242 | 4.99E-13 | 2.97E-11 | 19.8955095 |
| 439 | NA | 7973867 | -2.6024536 | 6.38867915 | -8.8663029 | 2.07E-08 | 2.60E-07 | 8.9551536 |
| 440 | IFITM2 | 7937330 | -2.6003378 | 8.4047792 | -17.419962 | 1.17E-13 | 8.97E-12 | 21.37583 |
| 441 | STEAP1 | 8134030 | -2.6003325 | 5.75731847 | -11.093604 | 4.66E-10 | 1.01E-08 | 12.8644857 |
| 442 | CA5B | 8166184 | -2.5968619 | 7.06553854 | -14.221399 | 5.29E-12 | 2.19E-10 | 17.4737542 |
| 443 | NA | 8112918 | -2.5944968 | 4.83159307 | -10.670355 | 9.18E-10 | 1.80E-08 | 12.1661396 |
| 444 | SCFD2 | 8100347 | -2.5913463 | 7.19515965 | -19.714299 | 1.09E-14 | 1.20E-12 | 23.7903272 |
| 445 | SNORA68 | 8026875 | -2.5908183 | 6.73887247 | -19.629731 | 1.19E-14 | 1.28E-12 | 23.7061644 |
| 446 | FBXO32 | 8152703 | -2.5885297 | 7.23708294 | -13.164963 | 2.18E-11 | 7.58E-10 | 16.0170941 |
| 447 | MGLL | 8090433 | -2.5866107 | 7.4271467 | -23.364838 | 4.03E-16 | 7.53E-14 | 27.1234445 |
| 448 | NA | 7894125 | -2.5788155 | 9.49446197 | -16.728406 | 2.53E-13 | 1.72E-11 | 20.5898961 |
| 449 | FAM198B | 8103415 | -2.5654716 | 5.40775898 | -15.922449 | 6.42E-13 | 3.69E-11 | 19.635906 |
| 450 | HIST1H3I | 8124531 | -2.5640313 | 7.53557488 | -11.463138 | 2.62E-10 | 6.15E-09 | 13.4583644 |
| 451 | VIM | 7926368 | -2.5567094 | 10.9725562 | -14.759371 | 2.66E-12 | 1.22E-10 | 18.1807574 |
| 452 | SNAI2 | 8150698 | -2.5558301 | 7.62798803 | -21.917628 | 1.40E-15 | 2.10E-13 | 25.8680447 |
| 453 | GSTO1 | 7930304 | -2.5556364 | 7.37629751 | -17.107724 | 1.65E-13 | 1.20E-11 | 21.0245795 |
| 454 | CSF1 | 7903786 | -2.5553702 | 8.27050498 | -21.88886 | 1.44E-15 | 2.14E-13 | 25.8422602 |
| 455 | RIPK3 | 7978312 | -2.5549255 | 6.47601716 | -12.987623 | 2.79E-11 | 9.39E-10 | 15.7631716 |
| 456 | HIGD1A | 7955719 | -2.5457695 | 6.85764676 | -12.584832 | 4.94E-11 | 1.54E-09 | 15.1758557 |
| 457 | RASA3 | 7972946 | -2.5426641 | 7.23573236 | -29.028478 | 5.62E-18 | 2.27E-15 | 31.3703434 |
| 458 | TIMP1 | 8167185 | -2.5397887 | 11.1523224 | -16.725496 | 2.54E-13 | 1.72E-11 | 20.5865264 |
| 459 | ERAP1 | 8113250 | -2.5392878 | 6.87937197 | -17.510159 | 1.06E-13 | 8.23E-12 | 21.4762188 |
| 460 | ENTPD1 | 7929511 | -2.5271772 | 5.93569202 | -7.6751871 | 2.03E-07 | 1.88E-06 | 6.60969565 |
| 461 | NA | 7892876 | -2.5262056 | 3.81731456 | -7.8341544 | 1.48E-07 | 1.43E-06 | 6.93355494 |
| 462 | IFIT5 | 7929072 | -2.5193429 | 6.31389997 | -10.892265 | 6.42E-10 | 1.33E-08 | 12.5347417 |
| 463 | DRAM1 | 7958019 | -2.5172837 | 8.21785203 | -9.7228493 | 4.50E-09 | 6.93E-08 | 10.5288089 |
| 464 | IFIH1 | 8056285 | -2.5163989 | 5.76793725 | -8.9387039 | 1.81E-08 | 2.32E-07 | 9.09174919 |
| 465 | GBP2 | 7917532 | -2.5160863 | 5.98542871 | -18.54931 | 3.53E-14 | 3.21E-12 | 22.5994256 |
| 466 | RARRES2 | 8143772 | -2.5157986 | 8.16239328 | -8.5483929 | 3.74E-08 | 4.35E-07 | 8.34736144 |
| 467 | TMEM119 | 7966122 | -2.5134536 | 7.43297625 | -20.934116 | 3.42E-15 | 4.44E-13 | 24.967026 |
| 468 | FAM129B | 8164217 | -2.5107956 | 8.91269446 | -20.606074 | 4.65E-15 | 5.73E-13 | 24.6572504 |
| 469 | PMS2P5 | 8140269 | -2.5019656 | 6.15088539 | -10.855696 | 6.81E-10 | 1.40E-08 | 12.4743749 |
| 470 | NA | 7895153 | -2.4978379 | 7.07913655 | -9.2254463 | 1.08E-08 | 1.48E-07 | 9.62617088 |
| 471 | UBE2D3 | 8180331 | -2.49757 | 5.86824344 | -6.1581916 | 4.85E-06 | 3.01E-05 | 3.35413761 |
| 472 | SLC15A3 | 7948493 | -2.4942285 | 6.90062879 | -13.184312 | 2.12E-11 | 7.42E-10 | 16.0446292 |
| 473 | NA | 7892650 | -2.4939548 | 10.7358391 | -17.316807 | 1.31E-13 | 9.88E-12 | 21.2604297 |
| 474 | LAYN | 7943749 | -2.4934123 | 7.37081689 | -12.256616 | 7.95E-11 | 2.25E-09 | 14.6860792 |
| 475 | SACS | 7970569 | -2.4899008 | 6.76150887 | -12.755891 | 3.87E-11 | 1.25E-09 | 15.4270991 |
| 476 | SCN9A | 8056491 | -2.4853846 | 6.43471514 | -10.916201 | 6.18E-10 | 1.29E-08 | 12.5741748 |
| 477 | MSC | 8151334 | -2.4765821 | 6.90674044 | -17.038254 | 1.79E-13 | 1.27E-11 | 20.9456315 |
| 478 | ANTXR1 | 8042439 | -2.4761795 | 8.91232128 | -19.701543 | 1.11E-14 | 1.20E-12 | 23.7776548 |
| 479 | WNT5B | 7953012 | -2.4713181 | 7.68330831 | -19.039829 | 2.14E-14 | 2.12E-12 | 23.1092745 |
| 480 | TAP2 | 8125483 | -2.4703686 | 4.5291607 | -8.3696765 | 5.24E-08 | 5.82E-07 | 7.99990656 |
| 481 | SLC24A3 | 8061227 | -2.4683304 | 6.29638758 | -16.648935 | 2.77E-13 | 1.81E-11 | 20.4976913 |
| 482 | NA | 7944765 | -2.4655839 | 3.42858997 | -13.162462 | 2.19E-11 | 7.58E-10 | 16.0135323 |
| 483 | NPIPL3 | 8000676 | -2.4627262 | 6.76409031 | -22.42276 | 9.00E-16 | 1.40E-13 | 26.3153944 |
| 484 | TWIST2 | 8180300 | -2.4626554 | 8.66586487 | -18.016528 | 6.16E-14 | 5.24E-12 | 22.0310847 |
| 485 | ANGPTL2 | 8164200 | -2.4620691 | 7.64852805 | -18.239333 | 4.87E-14 | 4.23E-12 | 22.2706483 |
| 486 | NA | 7895263 | -2.4604806 | 9.77513733 | -12.911877 | 3.11E-11 | 1.03E-09 | 15.6538577 |
| 487 | NA | 7892668 | -2.4596639 | 8.54001469 | -12.535534 | 5.30E-11 | 1.63E-09 | 15.1029422 |
| 488 | LOX | 8113709 | -2.4576279 | 9.85061989 | -11.27379 | 3.51E-10 | 7.94E-09 | 13.155874 |
| 489 | FRMD6 | 7974316 | -2.4571129 | 8.6551772 | -10.603087 | 1.02E-09 | 1.97E-08 | 12.0533187 |
| 490 | GSTK1 | 8136849 | -2.45539 | 6.835838 | -22.215286 | 1.08E-15 | 1.66E-13 | 26.1328779 |
| 491 | TSPYL5 | 8151931 | -2.4492397 | 5.8498037 | -13.711904 | 1.04E-11 | 3.90E-10 | 16.7829273 |
| 492 | NA | 7895444 | -2.4459095 | 9.05917463 | -10.517352 | 1.18E-09 | 2.20E-08 | 11.9087833 |
| 493 | EPSTI1 | 7971296 | -2.4433128 | 4.79748796 | -10.018362 | 2.71E-09 | 4.50E-08 | 11.0507817 |
| 494 | CFLAR | 8047401 | -2.442185 | 4.53876465 | -11.362365 | 3.06E-10 | 7.03E-09 | 13.2978455 |
| 495 | SMURF2 | 8017651 | -2.4344939 | 9.00145516 | -9.2652735 | 1.01E-08 | 1.39E-07 | 9.69957754 |
| 496 | NA | 7892682 | -2.4334385 | 3.49693868 | -5.5701583 | 1.80E-05 | 9.50E-05 | 2.0190226 |
| 497 | PRKG1 | 7927606 | -2.4307883 | 5.76867643 | -16.253796 | 4.36E-13 | 2.66E-11 | 20.0332496 |
| 498 | TMEM45A | 8081288 | -2.4302174 | 6.54121628 | -12.238579 | 8.17E-11 | 2.29E-09 | 14.6588653 |
| 499 | GAS1 | 8162179 | -2.4284925 | 8.07845732 | -17.637672 | 9.25E-14 | 7.31E-12 | 21.6173308 |
| 500 | FAS | 7929032 | -2.4276706 | 6.60115109 | -18.872363 | 2.53E-14 | 2.42E-12 | 22.9366185 |
| 501 | CACNA1C | 7953040 | -2.4228211 | 6.49475256 | -27.468968 | 1.67E-17 | 5.52E-15 | 30.2939379 |
| 502 | PLCB4 | 8060897 | -2.4185422 | 6.38121739 | -7.4128436 | 3.43E-07 | 2.96E-06 | 6.06790157 |
| 503 | SP100 | 8048940 | -2.4066519 | 6.08341199 | -11.918557 | 1.31E-10 | 3.43E-09 | 14.1707306 |
| 504 | DUSP10 | 7924450 | -2.4052925 | 6.53458122 | -14.471458 | 3.83E-12 | 1.66E-10 | 17.8051786 |
| 505 | USP53 | 8097098 | -2.4037112 | 8.54764419 | -8.7569237 | 2.53E-08 | 3.11E-07 | 8.74751451 |
| 506 | MRC2 | 8009040 | -2.3996185 | 8.33762174 | -13.845163 | 8.67E-12 | 3.36E-10 | 16.9656691 |
| 507 | GUSBP11 | 8074925 | -2.3989062 | 7.19453249 | -10.17557 | 2.08E-09 | 3.58E-08 | 11.324228 |
| 508 | NA | 7892836 | -2.3979792 | 10.0940522 | -13.952027 | 7.53E-12 | 2.99E-10 | 17.1111546 |
| 509 | ZNF83 | 8038967 | -2.3918494 | 5.43400176 | -15.330702 | 1.31E-12 | 6.78E-11 | 18.9076571 |
| 510 | GK | 8174103 | -2.3904726 | 7.5195791 | -10.601826 | 1.03E-09 | 1.97E-08 | 12.0511986 |
| 511 | TNFRSF10B | 8149733 | -2.3904137 | 7.21498531 | -17.605743 | 9.57E-14 | 7.51E-12 | 21.5820853 |
| 512 | GBA | 7920697 | -2.3876774 | 6.41917765 | -19.119722 | 1.97E-14 | 1.98E-12 | 23.1911357 |
| 513 | CREB3L1 | 7939642 | -2.3864646 | 8.04713687 | -16.125274 | 5.06E-13 | 3.00E-11 | 19.879996 |
| 514 | CLEC2B | 7961083 | -2.3856065 | 4.77944024 | -10.406681 | 1.41E-09 | 2.57E-08 | 11.7209737 |
| 515 | LPAR3 | 7917276 | -2.3812476 | 6.80311491 | -19.997476 | 8.30E-15 | 9.52E-13 | 24.0696435 |
| 516 | LAMB2 | 8087337 | -2.3787719 | 8.74628731 | -16.491385 | 3.31E-13 | 2.10E-11 | 20.313713 |
| 517 | NA | 7895599 | -2.3762942 | 5.8017976 | -10.509784 | 1.19E-09 | 2.22E-08 | 11.8959857 |
| 518 | MRGPRF | 7950005 | -2.3761818 | 7.11786073 | -19.892892 | 9.19E-15 | 1.05E-12 | 23.9669309 |
| 519 | NAV3 | 7957298 | -2.3746525 | 6.8858989 | -8.1348613 | 8.22E-08 | 8.56E-07 | 7.53700411 |
| 520 | NA | 7895885 | -2.3693325 | 8.67014265 | -14.837762 | 2.41E-12 | 1.12E-10 | 18.2819251 |
| 521 | SNORA22 | 8133106 | -2.3671309 | 5.11236694 | -14.286325 | 4.86E-12 | 2.04E-10 | 17.5602793 |
| 522 | APOL6 | 8072710 | -2.3667161 | 5.75945703 | -12.294263 | 7.53E-11 | 2.15E-09 | 14.7427802 |
| 523 | NA | 7894108 | -2.3647893 | 3.45791657 | -11.665099 | 1.92E-10 | 4.71E-09 | 13.7768856 |
| 524 | THBS1 | 7982597 | -2.3564533 | 12.0750441 | -9.9879081 | 2.85E-09 | 4.71E-08 | 10.9974737 |
| 525 | LOC654433 | 8044605 | -2.3558651 | 5.94251274 | -17.438698 | 1.15E-13 | 8.83E-12 | 21.3967227 |
| 526 | ADAM12 | 7936968 | -2.3540219 | 6.2820247 | -11.775334 | 1.63E-10 | 4.10E-09 | 13.9489763 |
| 527 | PTN | 8143144 | -2.3533028 | 6.86353781 | -17.014089 | 1.83E-13 | 1.30E-11 | 20.9180998 |
| 528 | RETSAT | 8053406 | -2.3521743 | 7.54253936 | -31.12143 | 1.41E-18 | 8.31E-16 | 32.7209583 |
| 529 | ARL6IP5 | 8080926 | -2.3510993 | 9.25698942 | -15.650182 | 8.88E-13 | 4.90E-11 | 19.3038576 |
| 530 | CACNA2D1 | 8140579 | -2.3479056 | 7.10343425 | -9.3573497 | 8.54E-09 | 1.21E-07 | 9.86852479 |
| 531 | MXRA5 | 8171172 | -2.3468953 | 7.1221185 | -8.0070247 | 1.05E-07 | 1.06E-06 | 7.28193071 |
| 532 | GPR124 | 8145865 | -2.3407104 | 8.01241033 | -21.931553 | 1.39E-15 | 2.09E-13 | 25.880513 |
| 533 | FBLN2 | 8077970 | -2.3372974 | 7.65901729 | -13.255511 | 1.93E-11 | 6.80E-10 | 16.1456684 |
| 534 | SGIP1 | 7902127 | -2.3361313 | 5.48158523 | -17.832158 | 7.50E-14 | 6.08E-12 | 21.8307507 |
| 535 | DDX58 | 8160559 | -2.3287401 | 6.58849967 | -6.6139445 | 1.81E-06 | 1.28E-05 | 4.36262857 |
| 536 | ANO10 | 8086467 | -2.3283734 | 7.42316448 | -12.4749 | 5.79E-11 | 1.76E-09 | 15.0129494 |
| 537 | NA | 7894315 | -2.3279428 | 6.25367296 | -5.4127716 | 2.57E-05 | 0.00012971 | 1.65609193 |
| 538 | ANKRD29 | 8022559 | -2.3215798 | 7.04046271 | -26.248348 | 4.10E-17 | 1.13E-14 | 29.4052944 |
| 539 | DZIP1 | 7972336 | -2.3207094 | 6.6147134 | -16.73323 | 2.52E-13 | 1.71E-11 | 20.5954801 |
| 540 | CD44 | 7939341 | -2.3200844 | 9.750338 | -12.019346 | 1.13E-10 | 3.03E-09 | 14.3255528 |
| 541 | PRCP | 7950731 | -2.3136224 | 6.80714875 | -15.551939 | 1.00E-12 | 5.44E-11 | 19.1827878 |
| 542 | PARP4 | 7970602 | -2.3105931 | 6.98212899 | -12.575954 | 5.01E-11 | 1.56E-09 | 15.1627405 |
| 543 | FAM21C | 7927233 | -2.3067485 | 6.88392088 | -7.1383051 | 6.02E-07 | 4.89E-06 | 5.49120085 |
| 544 | STAMBPL1 | 7929012 | -2.3036521 | 5.45354994 | -12.551473 | 5.18E-11 | 1.60E-09 | 15.1265408 |
| 545 | NA | 7893918 | -2.2994504 | 4.54334841 | -7.1594456 | 5.76E-07 | 4.70E-06 | 5.53595974 |
| 546 | NA | 7895810 | -2.2983243 | 5.65949002 | -11.176776 | 4.09E-10 | 9.04E-09 | 12.9994196 |
| 547 | FOLH1 | 7939897 | -2.2974681 | 8.70394803 | -15.27621 | 1.40E-12 | 7.17E-11 | 18.8393557 |
| 548 | SMPD1 | 7938100 | -2.2959484 | 8.02130335 | -17.070211 | 1.72E-13 | 1.24E-11 | 20.9819849 |
| 549 | FMOD | 7923578 | -2.2937565 | 6.14346117 | -17.995019 | 6.31E-14 | 5.32E-12 | 22.0078113 |
| 550 | ZNF587 | 8031827 | -2.2883569 | 5.03081203 | -15.492473 | 1.07E-12 | 5.73E-11 | 19.109175 |
| 551 | SLC2A10 | 8063177 | -2.2854033 | 8.0456899 | -17.537385 | 1.03E-13 | 8.03E-12 | 21.5064277 |
| 552 | ANGPT1 | 8152297 | -2.2851065 | 5.46010515 | -10.421944 | 1.38E-09 | 2.52E-08 | 11.7469587 |
| 553 | NA | 7895518 | -2.2782003 | 8.86043967 | -10.847161 | 6.90E-10 | 1.42E-08 | 12.4602646 |
| 554 | SGCB | 8100318 | -2.2779566 | 8.53881663 | -13.46573 | 1.44E-11 | 5.29E-10 | 16.4414143 |
| 555 | ABCA6 | 8017964 | -2.2762457 | 4.71420567 | -10.467734 | 1.28E-09 | 2.36E-08 | 11.8247555 |
| 556 | NA | 7894439 | -2.2712409 | 9.27090887 | -9.1008589 | 1.35E-08 | 1.80E-07 | 9.39525008 |
| 557 | NA | 7895873 | -2.2697268 | 9.98430529 | -10.326808 | 1.61E-09 | 2.88E-08 | 11.5845568 |
| 558 | RBMS1 | 8056201 | -2.2682432 | 7.78803408 | -16.278943 | 4.23E-13 | 2.60E-11 | 20.0631076 |
| 559 | UBB | 8005166 | -2.2668335 | 10.3713084 | -11.359128 | 3.08E-10 | 7.06E-09 | 13.2926714 |
| 560 | RAB3B | 7916112 | -2.2668209 | 8.00747433 | -16.934845 | 2.00E-13 | 1.41E-11 | 20.8275675 |
| 561 | FGFR1 | 8150318 | -2.2636125 | 7.99278959 | -26.275377 | 4.02E-17 | 1.12E-14 | 29.4254376 |
| 562 | SECTM1 | 8019486 | -2.261571 | 6.81764935 | -18.343983 | 4.37E-14 | 3.87E-12 | 22.3822271 |
| 563 | RAB27B | 8021301 | -2.2595223 | 6.01085411 | -16.298827 | 4.14E-13 | 2.55E-11 | 20.086688 |
| 564 | LOC100288114 | 8031646 | -2.2579528 | 5.66631593 | -10.264798 | 1.79E-09 | 3.15E-08 | 11.4781393 |
| 565 | NBPF10 | 7912808 | -2.2578369 | 6.88428387 | -14.420262 | 4.09E-12 | 1.76E-10 | 17.7377239 |
| 566 | SPON2 | 8098870 | -2.2569455 | 7.12214115 | -17.074874 | 1.71E-13 | 1.23E-11 | 20.987284 |
| 567 | GLRX | 8113214 | -2.2564128 | 7.11206186 | -12.599858 | 4.84E-11 | 1.51E-09 | 15.1980334 |
| 568 | ADM | 7938390 | -2.2558776 | 10.2121516 | -7.1645847 | 5.70E-07 | 4.65E-06 | 5.54683163 |
| 569 | RNF185 | 8072454 | -2.2524328 | 5.92387592 | -14.781508 | 2.58E-12 | 1.19E-10 | 18.2093732 |
| 570 | TBRG1 | 7944850 | -2.2521958 | 6.14931957 | -22.707403 | 7.04E-16 | 1.16E-13 | 26.5630749 |
| 571 | SAV1 | 7979033 | -2.2488097 | 7.32592333 | -14.548582 | 3.47E-12 | 1.54E-10 | 17.9064098 |
| 572 | MR1 | 7907893 | -2.2481114 | 5.77373423 | -15.507976 | 1.05E-12 | 5.67E-11 | 19.1283906 |
| 573 | SNORD54 | 8150877 | -2.2472141 | 3.62106101 | -13.174218 | 2.15E-11 | 7.51E-10 | 16.0302685 |
| 574 | SNX33 | 7985016 | -2.24591 | 6.7738616 | -20.238276 | 6.59E-15 | 7.74E-13 | 24.3041798 |
| 575 | NA | 7896423 | -2.2448066 | 5.2070651 | -8.6590636 | 3.04E-08 | 3.64E-07 | 8.56043245 |
| 576 | HIST1H1B | 8124527 | -2.2430677 | 6.75088309 | -8.1847414 | 7.46E-08 | 7.89E-07 | 7.63594373 |
| 577 | SUB1 | 8104738 | -2.2426445 | 8.1482851 | -10.180974 | 2.06E-09 | 3.55E-08 | 11.3335748 |
| 578 | BDKRB2 | 7976560 | -2.2419177 | 7.71357456 | -22.851627 | 6.22E-16 | 1.05E-13 | 26.6873877 |
| 579 | RPL21 | 7936727 | -2.2399409 | 10.0402811 | -15.388155 | 1.22E-12 | 6.39E-11 | 18.9794387 |
| 580 | NA | 7892688 | -2.2346203 | 5.85937206 | -12.181925 | 8.87E-11 | 2.45E-09 | 14.5731811 |
| 581 | NA | 7896228 | -2.2330162 | 6.3284523 | -5.4312582 | 2.47E-05 | 0.00012487 | 1.69882855 |
| 582 | IFNAR1 | 8068266 | -2.2322503 | 6.55041679 | -10.513718 | 1.19E-09 | 2.21E-08 | 11.902639 |
| 583 | ETV1 | 8138289 | -2.2286488 | 5.61114564 | -9.1786008 | 1.17E-08 | 1.60E-07 | 9.53957339 |
| 584 | PSTPIP2 | 8023043 | -2.2277944 | 4.9420087 | -12.437223 | 6.11E-11 | 1.84E-09 | 14.9568539 |
| 585 | DPYD | 7917912 | -2.2249271 | 6.59644443 | -16.665064 | 2.72E-13 | 1.80E-11 | 20.516437 |
| 586 | NA | 7892941 | -2.220972 | 10.2956075 | -12.90448 | 3.14E-11 | 1.04E-09 | 15.6431535 |
| 587 | TRIM16 | 8012953 | -2.2193388 | 7.68673984 | -12.737046 | 3.98E-11 | 1.28E-09 | 15.3995535 |
| 588 | OPTN | 7926239 | -2.2192211 | 8.25318073 | -23.342014 | 4.11E-16 | 7.63E-14 | 27.1042578 |
| 589 | LRRK2 | 7954810 | -2.2170948 | 4.82436374 | -13.901575 | 8.05E-12 | 3.16E-10 | 17.0425862 |
| 590 | S100A11 | 7920128 | -2.2151419 | 9.8410954 | -10.102991 | 2.35E-09 | 3.96E-08 | 11.1983473 |
| 591 | CPT1A | 7949971 | -2.2132479 | 7.19317897 | -12.584898 | 4.94E-11 | 1.54E-09 | 15.1759522 |
| 592 | CYBASC3 | 7948565 | -2.2129295 | 8.41529143 | -16.497483 | 3.29E-13 | 2.09E-11 | 20.3208634 |
| 593 | LOXL4 | 7935553 | -2.2124807 | 6.57340491 | -16.293561 | 4.16E-13 | 2.56E-11 | 20.0804459 |
| 594 | RABL2A | 8044669 | -2.2124263 | 4.41022518 | -4.9935954 | 6.74E-05 | 0.00030199 | 0.68051418 |
| 595 | SUCLA2 | 7971541 | -2.2120449 | 7.22662928 | -15.137545 | 1.66E-12 | 8.22E-11 | 18.6645846 |
| 596 | LRRN3 | 8135488 | -2.2074651 | 5.6691222 | -11.938186 | 1.27E-10 | 3.34E-09 | 14.2009611 |
| 597 | NA | 7986527 | -2.2064087 | 8.25666395 | -14.881166 | 2.28E-12 | 1.07E-10 | 18.3377409 |
| 598 | NA | 7894355 | -2.2057411 | 7.0633787 | -7.6171063 | 2.27E-07 | 2.08E-06 | 6.49053278 |
| 599 | MYOF | 7935058 | -2.2015377 | 9.34770796 | -9.7784128 | 4.08E-09 | 6.39E-08 | 10.6277545 |
| 600 | ARSB | 8112807 | -2.2000801 | 7.36558076 | -17.935559 | 6.72E-14 | 5.52E-12 | 21.9433407 |
| 601 | NA | 7893908 | -2.1989608 | 3.57983532 | -6.2087192 | 4.35E-06 | 2.73E-05 | 3.46714468 |
| 602 | CD14 | 8114612 | -2.1975037 | 6.68467495 | -12.254457 | 7.98E-11 | 2.26E-09 | 14.6828237 |
| 603 | NBPF15 | 7904976 | -2.1932859 | 8.48911376 | -15.191408 | 1.55E-12 | 7.76E-11 | 18.732638 |
| 604 | CARD16 | 7951408 | -2.1915511 | 4.91247192 | -16.517642 | 3.21E-13 | 2.06E-11 | 20.3444847 |
| 605 | MAB21L1 | 7970949 | -2.1903991 | 5.86753064 | -10.415374 | 1.39E-09 | 2.54E-08 | 11.7357768 |
| 606 | NA | 7893131 | -2.1876347 | 9.30013055 | -8.0381681 | 9.91E-08 | 1.01E-06 | 7.34427087 |
| 607 | TBC1D3F | 8014603 | -2.1868742 | 4.90019283 | -7.4302829 | 3.31E-07 | 2.88E-06 | 6.10419998 |
| 608 | HSBP1 | 7997520 | -2.1865198 | 7.75249793 | -24.677574 | 1.38E-16 | 3.16E-14 | 28.1961515 |
| 609 | COX6B1 | 8027932 | -2.1821512 | 7.65995743 | -23.690686 | 3.07E-16 | 6.02E-14 | 27.3953214 |
| 610 | FAM111B | 7940147 | -2.177663 | 4.72366041 | -7.1410614 | 5.99E-07 | 4.86E-06 | 5.49703978 |
| 611 | NA | 7966068 | -2.1755471 | 4.90933451 | -10.873491 | 6.62E-10 | 1.37E-08 | 12.5037694 |
| 612 | NA | 8013523 | -2.1742394 | 4.01484017 | -12.138775 | 9.46E-11 | 2.58E-09 | 14.5077107 |
| 613 | PITPNC1 | 8009353 | -2.1724186 | 6.27251 | -12.334334 | 7.10E-11 | 2.06E-09 | 14.8029814 |
| 614 | NA | 7896094 | -2.1723578 | 6.74619333 | -13.079974 | 2.46E-11 | 8.39E-10 | 15.8957533 |
| 615 | RGPD5 | 8054532 | -2.1702135 | 5.64788056 | -6.3053506 | 3.52E-06 | 2.27E-05 | 3.68245025 |
| 616 | RPS23 | 8112961 | -2.168261 | 7.50753719 | -13.60353 | 1.20E-11 | 4.49E-10 | 16.6332147 |
| 617 | IRS1 | 8059470 | -2.1677255 | 6.54282332 | -19.061649 | 2.09E-14 | 2.08E-12 | 23.1316638 |
| 618 | AOX1 | 8047300 | -2.1662511 | 6.18779755 | -16.80557 | 2.32E-13 | 1.59E-11 | 20.679045 |
| 619 | PARD3B | 8047709 | -2.1634821 | 6.9766543 | -9.3603282 | 8.49E-09 | 1.20E-07 | 9.8739722 |
| 620 | CDCP1 | 8086517 | -2.1618478 | 7.07162038 | -10.835665 | 7.03E-10 | 1.44E-08 | 12.4412459 |
| 621 | FCF1 | 7975713 | -2.159714 | 5.0860596 | -13.426507 | 1.52E-11 | 5.54E-10 | 16.3865231 |
| 622 | UFM1 | 7968670 | -2.1570778 | 7.88366021 | -16.564305 | 3.05E-13 | 1.98E-11 | 20.3990616 |
| 623 | FAM20C | 8130993 | -2.1568252 | 8.11836534 | -17.075762 | 1.71E-13 | 1.23E-11 | 20.9882924 |
| 624 | SLC22A4 | 8107909 | -2.1565441 | 6.00216253 | -10.525691 | 1.16E-09 | 2.18E-08 | 11.922878 |
| 625 | ELTD1 | 7917182 | -2.1563235 | 4.11452046 | -9.4325881 | 7.47E-09 | 1.08E-07 | 10.0057921 |
| 626 | CXCL10 | 8101126 | -2.1499298 | 4.34708407 | -4.3555776 | 0.00029879 | 0.00109663 | -0.8187968 |
| 627 | CYP7B1 | 8151056 | -2.1484275 | 7.19345833 | -10.786013 | 7.61E-10 | 1.55E-08 | 12.3589344 |
| 628 | CAB39L | 7971590 | -2.1462056 | 5.43605607 | -10.43244 | 1.36E-09 | 2.48E-08 | 11.7648134 |
| 629 | SPARC | 8115327 | -2.1408287 | 10.6779814 | -15.94717 | 6.24E-13 | 3.60E-11 | 19.6658043 |
| 630 | SNORD114-3 | 7976816 | -2.1404401 | 4.61797578 | -12.753763 | 3.88E-11 | 1.25E-09 | 15.4239912 |
| 631 | SLC38A5 | 8172425 | -2.1402502 | 6.85719483 | -13.261618 | 1.91E-11 | 6.76E-10 | 16.1543149 |
| 632 | SEC22C | 8086406 | -2.1379618 | 6.86126596 | -15.929234 | 6.37E-13 | 3.67E-11 | 19.6441159 |
| 633 | GSTM5 | 7903777 | -2.1348355 | 5.45736267 | -12.614082 | 4.74E-11 | 1.49E-09 | 15.2190091 |
| 634 | MEG3 | 7976795 | -2.1326166 | 6.70151945 | -12.191188 | 8.75E-11 | 2.42E-09 | 14.5872117 |
| 635 | EDNRA | 8097692 | -2.1305976 | 4.95741189 | -10.401577 | 1.43E-09 | 2.59E-08 | 11.7122785 |
| 636 | CCDC80 | 8089544 | -2.1292964 | 7.37253745 | -11.526812 | 2.38E-10 | 5.67E-09 | 13.5592421 |
| 637 | C1orf198 | 7924996 | -2.1291637 | 8.1285806 | -20.154841 | 7.14E-15 | 8.25E-13 | 24.2232218 |
| 638 | PGF | 7980233 | -2.1290348 | 7.55492276 | -11.237339 | 3.72E-10 | 8.32E-09 | 13.0972066 |
| 639 | NA | 7895090 | -2.1238884 | 7.22186173 | -6.6987251 | 1.51E-06 | 1.09E-05 | 4.54746047 |
| 640 | SOCS2 | 7957551 | -2.1237814 | 7.03130557 | -12.968829 | 2.87E-11 | 9.60E-10 | 15.7360975 |
| 641 | NPR2 | 8155121 | -2.1209315 | 6.87762868 | -15.638985 | 9.00E-13 | 4.94E-11 | 19.2900927 |
| 642 | NA | 7896439 | -2.1208269 | 7.01366788 | -11.67612 | 1.89E-10 | 4.64E-09 | 13.7941458 |
| 643 | CTSZ | 8067279 | -2.1173222 | 8.70217969 | -24.196295 | 2.03E-16 | 4.38E-14 | 27.8097681 |
| 644 | DPYSL3 | 8114920 | -2.1171018 | 7.47668342 | -17.531561 | 1.04E-13 | 8.06E-12 | 21.4999699 |
| 645 | TGFBR1 | 8156826 | -2.116349 | 7.56986595 | -14.541048 | 3.50E-12 | 1.55E-10 | 17.8965419 |
| 646 | NA | 7895972 | -2.1139469 | 9.69118579 | -10.024907 | 2.68E-09 | 4.46E-08 | 11.0622243 |
| 647 | ZNF521 | 8022612 | -2.109121 | 6.2575969 | -14.388846 | 4.26E-12 | 1.82E-10 | 17.6962287 |
| 648 | BIRC3 | 7943413 | -2.1084967 | 5.5045057 | -11.716148 | 1.78E-10 | 4.42E-09 | 13.8567326 |
| 649 | PRTFDC1 | 7932584 | -2.1071817 | 4.72782411 | -17.911969 | 6.89E-14 | 5.64E-12 | 21.9177071 |
| 650 | SLC7A14 | 8092053 | -2.1010898 | 5.35661243 | -9.9825872 | 2.88E-09 | 4.75E-08 | 10.9881482 |
| 651 | MFSD6 | 8047078 | -2.1004146 | 6.49181417 | -14.747291 | 2.70E-12 | 1.24E-10 | 18.1651268 |
| 652 | CAMK2N1 | 7913237 | -2.0988134 | 8.63935876 | -18.738841 | 2.90E-14 | 2.70E-12 | 22.7979176 |
| 653 | SNORD113-4 | 7976812 | -2.098202 | 3.69235576 | -10.121826 | 2.27E-09 | 3.86E-08 | 11.2310738 |
| 654 | FAM46A | 8127778 | -2.0965917 | 6.93861253 | -14.931364 | 2.14E-12 | 1.02E-10 | 18.4021185 |
| 655 | MIR221 | 8172266 | -2.0962513 | 5.33344643 | -9.2867362 | 9.68E-09 | 1.35E-07 | 9.73905335 |
| 656 | GULP1 | 8046906 | -2.0951701 | 5.28938682 | -14.27267 | 4.95E-12 | 2.07E-10 | 17.5421092 |
| 657 | GGH | 8151032 | -2.0949228 | 6.65428207 | -13.575723 | 1.24E-11 | 4.63E-10 | 16.5946402 |
| 658 | PDZRN3 | 8088848 | -2.0880877 | 6.60548865 | -13.407635 | 1.56E-11 | 5.66E-10 | 16.3600655 |
| 659 | TTC3 | 8068522 | -2.0880524 | 6.83952999 | -14.362847 | 4.40E-12 | 1.87E-10 | 17.6618307 |
| 660 | PDE4DIP | 8180359 | -2.0854882 | 8.25939017 | -17.13456 | 1.60E-13 | 1.17E-11 | 21.054998 |
| 661 | HIST1H2BK | 8124492 | -2.0845245 | 6.47204165 | -6.8717076 | 1.05E-06 | 7.92E-06 | 4.92178655 |
| 662 | CXCL2 | 8100994 | -2.0831078 | 6.581694 | -6.5205068 | 2.21E-06 | 1.52E-05 | 4.15789504 |
| 663 | SNX19 | 7952768 | -2.0821009 | 7.22538688 | -14.407372 | 4.16E-12 | 1.78E-10 | 17.7207082 |
| 664 | SNORD49B | 8005200 | -2.0782166 | 3.77308033 | -7.7106962 | 1.89E-07 | 1.77E-06 | 6.68232839 |
| 665 | IGFBP6 | 7955694 | -2.0770919 | 8.94592328 | -16.70383 | 2.60E-13 | 1.74E-11 | 20.5614249 |
| 666 | PGRMC1 | 8169617 | -2.0749716 | 9.78943192 | -14.013806 | 6.94E-12 | 2.78E-10 | 17.1948329 |
| 667 | NA | 7892776 | -2.0700493 | 7.60758836 | -9.8267263 | 3.76E-09 | 5.96E-08 | 10.7134861 |
| 668 | SNORD68 | 7997940 | -2.0696073 | 5.96363633 | -13.507566 | 1.36E-11 | 5.03E-10 | 16.4998148 |
| 669 | DOCK4 | 8142345 | -2.0688689 | 6.51472846 | -14.573849 | 3.36E-12 | 1.50E-10 | 17.9394758 |
| 670 | RAB1B | 7941583 | -2.0672912 | 8.74222251 | -16.82738 | 2.26E-13 | 1.56E-11 | 20.704175 |
| 671 | CACNA2D3 | 8080578 | -2.0669856 | 5.79884657 | -17.718981 | 8.47E-14 | 6.76E-12 | 21.706819 |
| 672 | NA | 7895290 | -2.0665267 | 7.24389087 | -7.9664782 | 1.14E-07 | 1.14E-06 | 7.20057567 |
| 673 | NA | 7895149 | -2.0657823 | 10.1497138 | -14.085274 | 6.32E-12 | 2.57E-10 | 17.2912489 |
| 674 | MSRB2 | 7926661 | -2.0647748 | 7.72755309 | -15.446188 | 1.14E-12 | 6.01E-11 | 19.0517081 |
| 675 | NA | 7893074 | -2.061693 | 5.84627839 | -8.0179555 | 1.03E-07 | 1.04E-06 | 7.30382573 |
| 676 | NA | 7894837 | -2.061294 | 5.93083231 | -8.4487849 | 4.51E-08 | 5.11E-07 | 8.15422266 |
| 677 | ZC3H12C | 7943715 | -2.0606503 | 5.86149521 | -11.385846 | 2.95E-10 | 6.81E-09 | 13.3353428 |
| 678 | ISM1 | 8061013 | -2.0597081 | 6.3168226 | -15.806673 | 7.37E-13 | 4.15E-11 | 19.4953292 |
| 679 | LIMA1 | 7963187 | -2.0596382 | 8.62242838 | -15.600842 | 9.43E-13 | 5.16E-11 | 19.2431371 |
| 680 | GCNT4 | 8112668 | -2.0594438 | 5.10993232 | -9.6689496 | 4.94E-09 | 7.51E-08 | 10.4324679 |
| 681 | KCNMA1 | 7934570 | -2.0577525 | 7.28637983 | -12.019281 | 1.13E-10 | 3.03E-09 | 14.3254534 |
| 682 | RPL23AP82 | 8074157 | -2.0576652 | 7.37890141 | -16.973822 | 1.92E-13 | 1.36E-11 | 20.8721455 |
| 683 | ARPC3 | 7966315 | -2.0524941 | 8.72673332 | -10.909811 | 6.24E-10 | 1.30E-08 | 12.5636538 |
| 684 | TFPI | 8057599 | -2.0519163 | 6.06107951 | -11.609486 | 2.09E-10 | 5.08E-09 | 13.6895948 |
| 685 | FGF10 | 8111993 | -2.0470306 | 5.50246235 | -9.3738211 | 8.29E-09 | 1.18E-07 | 9.89863598 |
| 686 | ARF4 | 8088339 | -2.0454462 | 9.8922523 | -20.210324 | 6.77E-15 | 7.89E-13 | 24.2770936 |
| 687 | ABHD2 | 7985809 | -2.0454239 | 7.69719026 | -13.809162 | 9.10E-12 | 3.49E-10 | 16.9164457 |
| 688 | RPL36AL | 7978833 | -2.0449355 | 7.7555888 | -16.204775 | 4.61E-13 | 2.78E-11 | 19.9749238 |
| 689 | NUPR1 | 8000574 | -2.0420876 | 7.5294601 | -16.625845 | 2.84E-13 | 1.85E-11 | 20.4708266 |
| 690 | NR4A2 | 8055952 | -2.0415619 | 6.10899326 | -5.6955011 | 1.36E-05 | 7.42E-05 | 2.30649972 |
| 691 | CFLAR | 8047381 | -2.037592 | 5.85256598 | -16.652563 | 2.76E-13 | 1.81E-11 | 20.5019094 |
| 692 | NA | 7895928 | -2.0359585 | 11.2632641 | -10.232297 | 1.89E-09 | 3.30E-08 | 11.4221854 |
| 693 | ARPC1B | 8134552 | -2.0344152 | 8.61159562 | -12.303394 | 7.43E-11 | 2.12E-09 | 14.7565119 |
| 694 | RPL41 | 7956159 | -2.0335053 | 4.87878541 | -13.805254 | 9.15E-12 | 3.50E-10 | 16.9110964 |
| 695 | ANXA6 | 8115234 | -2.0319888 | 9.70145299 | -14.924561 | 2.16E-12 | 1.02E-10 | 18.3934047 |
| 696 | AVPI1 | 7935521 | -2.0319385 | 7.00663316 | -10.587189 | 1.05E-09 | 2.00E-08 | 12.0265807 |
| 697 | CROT | 8133938 | -2.0283283 | 6.90317437 | -6.9037023 | 9.81E-07 | 7.46E-06 | 4.99060569 |
| 698 | AKR1B1 | 8143054 | -2.0282696 | 8.66226456 | -7.2433172 | 4.85E-07 | 4.02E-06 | 5.71295899 |
| 699 | ANXA5 | 8102619 | -2.0269686 | 11.381097 | -11.863587 | 1.42E-10 | 3.66E-09 | 14.0858626 |
| 700 | LGALS3BP | 8018975 | -2.0268738 | 8.70055384 | -15.074532 | 1.79E-12 | 8.81E-11 | 18.5847009 |
| 701 | CTSO | 8103389 | -2.022819 | 5.60287665 | -14.334213 | 4.57E-12 | 1.93E-10 | 17.6238858 |
| 702 | AGSK1 | 7985431 | -2.02125 | 4.97573223 | -7.8977877 | 1.30E-07 | 1.28E-06 | 7.06225275 |
| 703 | PAK2 | 8084963 | -2.0209741 | 7.07280472 | -10.509599 | 1.19E-09 | 2.22E-08 | 11.8956732 |
| 704 | TNFRSF19 | 7968015 | -2.0173305 | 7.19531317 | -17.066656 | 1.73E-13 | 1.24E-11 | 20.9779436 |
| 705 | NA | 7894416 | -2.0162672 | 9.13877134 | -8.3581157 | 5.36E-08 | 5.92E-07 | 7.97728625 |
| 706 | GK | 8166632 | -2.0157494 | 5.23825793 | -13.162684 | 2.19E-11 | 7.58E-10 | 16.013849 |
| 707 | NA | 7896622 | -2.0155794 | 10.91948 | -10.312652 | 1.65E-09 | 2.94E-08 | 11.5603026 |
| 708 | NA | 7895331 | -2.0112512 | 3.12406971 | -5.1033256 | 5.23E-05 | 0.00024157 | 0.93698001 |
| 709 | NA | 7892639 | -2.0077857 | 6.47076349 | -4.3415259 | 0.00030881 | 0.00113037 | -0.8518543 |
| 710 | IFI27 | 7976443 | -2.0077557 | 7.50614207 | -10.614734 | 1.01E-09 | 1.94E-08 | 12.0728889 |
| 711 | TMEM123 | 7951207 | -2.0070116 | 8.42101386 | -15.898013 | 6.61E-13 | 3.78E-11 | 19.6063106 |
| 712 | RPS27 | 7905691 | -2.006656 | 7.8053699 | -11.530463 | 2.36E-10 | 5.65E-09 | 13.5650137 |
| 713 | NA | 7892663 | -2.004811 | 10.8165964 | -13.036998 | 2.61E-11 | 8.86E-10 | 15.8341516 |
| 714 | LHFP | 7971150 | -2.0047679 | 8.1252013 | -26.287811 | 3.98E-17 | 1.12E-14 | 29.4346966 |
| 715 | SNORD15B | 7942594 | -2.0041152 | 6.0289029 | -13.8178 | 8.99E-12 | 3.45E-10 | 16.9282663 |
| 716 | ACIN1 | 7973371 | -2.0023002 | 6.42713565 | -15.045702 | 1.86E-12 | 9.04E-11 | 18.5480566 |
| 717 | TMX2 | 7940066 | -2.0011834 | 7.34051452 | -9.6152519 | 5.42E-09 | 8.13E-08 | 10.3361358 |
| 718 | CD109 | 8120719 | -1.9963907 | 8.27956856 | -14.74619 | 2.70E-12 | 1.24E-10 | 18.1637017 |
| 719 | NA | 7895611 | -1.9943768 | 3.71525749 | -5.5165356 | 2.03E-05 | 0.00010556 | 1.89560586 |
| 720 | SLC35F5 | 8054771 | -1.9925734 | 8.30326277 | -13.609363 | 1.19E-11 | 4.46E-10 | 16.6412979 |
| 721 | NA | 8114211 | -1.9912661 | 6.46196481 | -8.7158859 | 2.73E-08 | 3.32E-07 | 8.66921239 |
| 722 | LY96 | 8146934 | -1.9912357 | 4.04936237 | -9.4704629 | 6.99E-09 | 1.01E-07 | 10.0746261 |
| 723 | FAM35A | 7928909 | -1.9906831 | 5.2898716 | -7.4638873 | 3.09E-07 | 2.71E-06 | 6.17403129 |
| 724 | PCNXL4 | 7974771 | -1.9890312 | 6.82215114 | -9.1618076 | 1.21E-08 | 1.64E-07 | 9.50846237 |
| 725 | NA | 7894171 | -1.9817433 | 9.22215874 | -10.545728 | 1.12E-09 | 2.11E-08 | 11.9567126 |
| 726 | HECW2 | 8057898 | -1.9776722 | 5.83060123 | -10.941101 | 5.94E-10 | 1.25E-08 | 12.6151305 |
| 727 | APCDD1 | 8020141 | -1.9771351 | 7.57100873 | -12.373005 | 6.71E-11 | 1.97E-09 | 14.8609328 |
| 728 | MFGE8 | 7991234 | -1.9768041 | 9.10217068 | -17.943588 | 6.66E-14 | 5.52E-12 | 21.9520574 |
| 729 | SSFA2 | 8046726 | -1.9763925 | 8.37871121 | -15.342658 | 1.29E-12 | 6.70E-11 | 18.9226143 |
| 730 | MGST1 | 7954196 | -1.9738108 | 5.34198301 | -14.093883 | 6.25E-12 | 2.55E-10 | 17.3028353 |
| 731 | ITPRIPL2 | 7993622 | -1.9732009 | 7.26270971 | -14.567772 | 3.39E-12 | 1.51E-10 | 17.9315277 |
| 732 | NA | 7896082 | -1.9728162 | 10.2092553 | -9.2112437 | 1.11E-08 | 1.52E-07 | 9.59994536 |
| 733 | APH1B | 7984124 | -1.969999 | 6.91601017 | -11.242336 | 3.69E-10 | 8.30E-09 | 13.1052572 |
| 734 | NFKB1 | 8096635 | -1.9686016 | 6.95875131 | -8.7637054 | 2.50E-08 | 3.08E-07 | 8.7604334 |
| 735 | DYNLT3 | 8172035 | -1.9681003 | 7.31060723 | -9.2111006 | 1.11E-08 | 1.52E-07 | 9.59968102 |
| 736 | PIK3IP1 | 8075483 | -1.9675917 | 6.64959313 | -15.42567 | 1.16E-12 | 6.15E-11 | 19.0261837 |
| 737 | UBE2D3 | 8180329 | -1.9675031 | 5.88221107 | -5.5677591 | 1.81E-05 | 9.55E-05 | 2.01350598 |
| 738 | SNORA40 | 7951038 | -1.9646333 | 3.67827176 | -6.9771626 | 8.41E-07 | 6.53E-06 | 5.14811904 |
| 739 | NOV | 8148049 | -1.9620548 | 7.15213305 | -9.2833845 | 9.74E-09 | 1.36E-07 | 9.73289253 |
| 740 | APOL2 | 8075720 | -1.957411 | 5.75766381 | -14.511428 | 3.64E-12 | 1.59E-10 | 17.8576993 |
| 741 | USP12 | 7970696 | -1.9566476 | 5.49310785 | -6.6056115 | 1.84E-06 | 1.30E-05 | 4.34441361 |
| 742 | PLAT | 8150509 | -1.9543532 | 8.26315393 | -7.7272089 | 1.83E-07 | 1.72E-06 | 6.71604748 |
| 743 | IL12A | 8083690 | -1.9516578 | 5.78790901 | -6.5206541 | 2.21E-06 | 1.52E-05 | 4.15821866 |
| 744 | NA | 7893721 | -1.9505887 | 6.40580862 | -6.0323175 | 6.40E-06 | 3.84E-05 | 3.07137388 |
| 745 | ADAMTS12 | 8111387 | -1.9497423 | 6.07665521 | -10.828943 | 7.11E-10 | 1.46E-08 | 12.4301177 |
| 746 | NA | 7895012 | -1.9497398 | 8.80783893 | -7.8826711 | 1.34E-07 | 1.31E-06 | 7.03172831 |
| 747 | GPC6 | 7969613 | -1.9478025 | 6.8201813 | -13.256341 | 1.92E-11 | 6.80E-10 | 16.1468439 |
| 748 | FTH1 | 8170360 | -1.9422936 | 12.0447864 | -13.281904 | 1.86E-11 | 6.59E-10 | 16.1830099 |
| 749 | NA | 7893407 | -1.9403025 | 8.75509903 | -6.5505294 | 2.07E-06 | 1.43E-05 | 4.22379494 |
| 750 | CYLD | 7995552 | -1.9386949 | 6.82852596 | -11.124714 | 4.44E-10 | 9.72E-09 | 12.9150448 |
| 751 | TLN1 | 8161056 | -1.93822 | 8.9901768 | -15.491309 | 1.08E-12 | 5.73E-11 | 19.107732 |
| 752 | LINC00152 | 8054611 | -1.9372417 | 8.52163377 | -12.182289 | 8.87E-11 | 2.45E-09 | 14.5737324 |
| 753 | NA | 7948995 | -1.9325261 | 7.93524238 | -9.5803736 | 5.76E-09 | 8.56E-08 | 10.2733763 |
| 754 | NA | 7896047 | -1.9321709 | 8.2427655 | -22.645423 | 7.42E-16 | 1.20E-13 | 26.5094081 |
| 755 | RSU1 | 7932311 | -1.9318488 | 8.06250048 | -12.702584 | 4.18E-11 | 1.33E-09 | 15.3490953 |
| 756 | HIST1H4E | 8117402 | -1.9310588 | 5.92374782 | -10.181727 | 2.06E-09 | 3.54E-08 | 11.3348776 |
| 757 | PDE7B | 8122222 | -1.9303055 | 5.73592901 | -14.940354 | 2.12E-12 | 1.02E-10 | 18.4136286 |
| 758 | NDUFA5 | 8142663 | -1.9301542 | 5.54242157 | -13.486649 | 1.40E-11 | 5.16E-10 | 16.4706352 |
| 759 | RCN1 | 7939120 | -1.9290282 | 9.59706097 | -14.925796 | 2.16E-12 | 1.02E-10 | 18.3949867 |
| 760 | LAMA2 | 8121949 | -1.9289005 | 5.87679614 | -9.4162769 | 7.69E-09 | 1.10E-07 | 9.97609309 |
| 761 | DCTD | 8103859 | -1.9285247 | 8.62272703 | -12.248153 | 8.05E-11 | 2.27E-09 | 14.6733135 |
| 762 | MYADM | 8031047 | -1.9271846 | 8.23929127 | -17.988872 | 6.35E-14 | 5.33E-12 | 22.001156 |
| 763 | TMEM140 | 8136388 | -1.9239002 | 6.0984483 | -15.072518 | 1.80E-12 | 8.82E-11 | 18.5821433 |
| 764 | NA | 7895824 | -1.9230209 | 9.60936349 | -5.7121552 | 1.31E-05 | 7.18E-05 | 2.34458607 |
| 765 | NA | 7946565 | -1.9213727 | 13.4007797 | -8.5484193 | 3.74E-08 | 4.35E-07 | 8.34741237 |
| 766 | TMEM14B | 8116867 | -1.9200988 | 7.6052282 | -10.585972 | 1.05E-09 | 2.01E-08 | 12.024532 |
| 767 | DENND2A | 8143397 | -1.91959 | 6.7770754 | -18.263644 | 4.75E-14 | 4.17E-12 | 22.2966227 |
| 768 | TGFBI | 8108217 | -1.9168096 | 10.390833 | -13.088299 | 2.43E-11 | 8.30E-10 | 15.9076679 |
| 769 | TMBIM6 | 7955277 | -1.9103224 | 10.448451 | -13.823617 | 8.92E-12 | 3.43E-10 | 16.9362226 |
| 770 | RPL26 | 8012469 | -1.9102642 | 6.19652325 | -10.335628 | 1.59E-09 | 2.85E-08 | 11.5996574 |
| 771 | DSE | 8121588 | -1.9098285 | 7.43367983 | -12.4827 | 5.72E-11 | 1.74E-09 | 15.0245462 |
| 772 | PTGES | 8164580 | -1.9094979 | 7.25507587 | -10.141837 | 2.20E-09 | 3.75E-08 | 11.2657985 |
| 773 | PS1TP4 | 8054769 | -1.9091927 | 5.78924178 | -12.335609 | 7.09E-11 | 2.06E-09 | 14.8048933 |
| 774 | NA | 7895298 | -1.9091224 | 4.25213682 | -7.0344401 | 7.46E-07 | 5.88E-06 | 5.27045141 |
| 775 | FBXO30 | 8130032 | -1.9076662 | 6.98108427 | -10.84511 | 6.92E-10 | 1.42E-08 | 12.4568729 |
| 776 | SPRY2 | 7972217 | -1.9071939 | 6.34189978 | -17.973991 | 6.45E-14 | 5.38E-12 | 21.9850334 |
| 777 | SPATA13 | 7968035 | -1.9036338 | 6.67676275 | -13.433298 | 1.51E-11 | 5.49E-10 | 16.3960364 |
| 778 | MB21D1 | 8127534 | -1.9009264 | 4.38027953 | -11.144174 | 4.30E-10 | 9.46E-09 | 12.9466173 |
| 779 | PLEKHM1 | 8016239 | -1.9003279 | 6.17424585 | -10.795843 | 7.49E-10 | 1.53E-08 | 12.3752524 |
|  |  |  |  |  |  |  |  |  |
| **Genes Upregulated** | | |  |  |  |  |  |  |
| 1 | NA | 7894683 | 1.90039563 | 6.34852976 | 5.17971028 | 4.38E-05 | 0.00020704 | 1.11509553 |
| 2 | NA | 7902365 | 1.90693621 | 4.53190007 | 8.8536303 | 2.12E-08 | 2.65E-07 | 8.93117554 |
| 3 | PLK1 | 7994109 | 1.90847644 | 8.29161137 | 8.43618981 | 4.62E-08 | 5.22E-07 | 8.12970853 |
| 4 | SPAG5 | 8013671 | 1.91105384 | 7.33304659 | 9.53034687 | 6.29E-09 | 9.26E-08 | 10.183098 |
| 5 | NA | 7893477 | 1.91181218 | 8.0593504 | 7.3082059 | 4.25E-07 | 3.58E-06 | 5.84926513 |
| 6 | NA | 7895422 | 1.91255549 | 10.3564285 | 10.6035538 | 1.02E-09 | 1.97E-08 | 12.0541031 |
| 7 | BUB1B | 7982663 | 1.91631046 | 7.11300675 | 9.1029568 | 1.35E-08 | 1.79E-07 | 9.39915473 |
| 8 | HOXD10 | 8046536 | 1.91635226 | 5.57954121 | 18.9281366 | 2.39E-14 | 2.31E-12 | 22.9942811 |
| 9 | PPP1R9A | 8134351 | 1.917077 | 5.22789277 | 14.6985728 | 2.87E-12 | 1.30E-10 | 18.1019752 |
| 10 | NA | 7895214 | 1.91822076 | 10.6166058 | 8.93644387 | 1.82E-08 | 2.33E-07 | 9.08749548 |
| 11 | PHKA1 | 8173551 | 1.91947687 | 6.21844405 | 15.2410771 | 1.46E-12 | 7.40E-11 | 18.7952062 |
| 12 | NA | 7894008 | 1.92242229 | 6.09513213 | 9.42372972 | 7.59E-09 | 1.09E-07 | 9.98966705 |
| 13 | NA | 8143461 | 1.92406092 | 4.87275358 | 18.0854175 | 5.73E-14 | 4.91E-12 | 22.1054493 |
| 14 | PIK3CB | 8091009 | 1.92800818 | 7.98671936 | 13.512344 | 1.36E-11 | 5.01E-10 | 16.5064759 |
| 15 | PDK3 | 8166511 | 1.93153689 | 6.14920795 | 16.2644212 | 4.30E-13 | 2.63E-11 | 20.0458702 |
| 16 | NA | 7895373 | 1.93432602 | 11.426966 | 11.0539089 | 4.96E-10 | 1.07E-08 | 12.7998242 |
| 17 | NA | 7893639 | 1.93516509 | 10.7109894 | 17.055348 | 1.75E-13 | 1.25E-11 | 20.9650845 |
| 18 | DLGAP5 | 7979307 | 1.93994411 | 7.59425895 | 6.43645001 | 2.65E-06 | 1.77E-05 | 3.97280798 |
| 19 | CA9 | 8155083 | 1.94052696 | 7.74995679 | 9.95026319 | 3.04E-09 | 4.98E-08 | 10.9314248 |
| 20 | KIAA0895 | 8139021 | 1.94077485 | 6.39464436 | 17.8022644 | 7.75E-14 | 6.23E-12 | 21.7980873 |
| 21 | NA | 7894516 | 1.9422261 | 10.3540828 | 11.0655081 | 4.87E-10 | 1.05E-08 | 12.8187365 |
| 22 | TUBGCP4 | 7983206 | 1.94785199 | 7.31323832 | 12.4169556 | 6.30E-11 | 1.88E-09 | 14.9266236 |
| 23 | NA | 7893248 | 1.95049049 | 7.41066138 | 12.7715673 | 3.79E-11 | 1.22E-09 | 15.4499888 |
| 24 | NA | 7895726 | 1.95078626 | 8.58149576 | 10.7631056 | 7.90E-10 | 1.59E-08 | 12.3208684 |
| 25 | FLRT3 | 8065071 | 1.95485386 | 6.0519868 | 9.58032956 | 5.76E-09 | 8.56E-08 | 10.2732969 |
| 26 | PRKCI | 8083854 | 1.95562637 | 8.0204574 | 14.7449537 | 2.71E-12 | 1.24E-10 | 18.1621012 |
| 27 | NA | 7894451 | 1.95944741 | 7.64998292 | 4.87208624 | 8.93E-05 | 0.00038648 | 0.39581563 |
| 28 | CENPW | 8121911 | 1.96307743 | 6.14883205 | 8.63951585 | 3.15E-08 | 3.75E-07 | 8.52291366 |
| 29 | NA | 8112070 | 1.9630936 | 5.83643767 | 8.89238121 | 1.97E-08 | 2.49E-07 | 9.00443178 |
| 30 | NA | 7892764 | 1.96743427 | 10.0089072 | 11.8181585 | 1.53E-10 | 3.88E-09 | 14.015498 |
| 31 | KIAA1324L | 8140709 | 1.96895811 | 5.57510507 | 10.1839277 | 2.05E-09 | 3.53E-08 | 11.3386832 |
| 32 | GCLC | 8127158 | 1.9703615 | 7.47715362 | 7.62953703 | 2.22E-07 | 2.04E-06 | 6.5160742 |
| 33 | DOCK9 | 7972487 | 1.97753757 | 6.9841222 | 10.9809207 | 5.57E-10 | 1.18E-08 | 12.6804829 |
| 34 | MED12L | 8083360 | 1.97900406 | 5.46732797 | 13.7204717 | 1.02E-11 | 3.87E-10 | 16.7947207 |
| 35 | NA | 8104625 | 1.97911213 | 6.02696187 | 10.5011215 | 1.21E-09 | 2.25E-08 | 11.8813281 |
| 36 | WDR76 | 7983306 | 1.98195082 | 6.86280731 | 11.0495009 | 5.00E-10 | 1.07E-08 | 12.7926333 |
| 37 | JPH1 | 8151423 | 1.98779172 | 6.02340685 | 14.0631132 | 6.50E-12 | 2.63E-10 | 17.261397 |
| 38 | HOXA2 | 8138718 | 1.99154209 | 5.23727686 | 9.73034663 | 4.44E-09 | 6.85E-08 | 10.5421817 |
| 39 | CGNL1 | 7983867 | 1.99443954 | 5.43257351 | 20.8552572 | 3.68E-15 | 4.72E-13 | 24.8929925 |
| 40 | CDK2 | 7956076 | 1.99814618 | 7.91646398 | 13.4346525 | 1.51E-11 | 5.49E-10 | 16.3979334 |
| 41 | GPR56 | 7996081 | 1.99870443 | 6.27380066 | 17.8297831 | 7.52E-14 | 6.08E-12 | 21.8281573 |
| 42 | NA | 7893882 | 2.00240344 | 4.04506216 | 6.8463991 | 1.11E-06 | 8.30E-06 | 4.86725681 |
| 43 | GPR64 | 8171624 | 2.00628706 | 5.87411781 | 17.2013016 | 1.49E-13 | 1.10E-11 | 21.1304617 |
| 44 | NUF2 | 7906930 | 2.01239237 | 7.36103045 | 8.61329749 | 3.31E-08 | 3.92E-07 | 8.47251364 |
| 45 | TATDN1 | 8180269 | 2.01503028 | 7.433877 | 11.3715923 | 3.02E-10 | 6.93E-09 | 13.3125874 |
| 46 | NA | 7896341 | 2.01673547 | 7.37593229 | 14.8147717 | 2.48E-12 | 1.15E-10 | 18.2523029 |
| 47 | LPPR5 | 7917946 | 2.01725986 | 4.70306321 | 9.7421417 | 4.35E-09 | 6.75E-08 | 10.5632065 |
| 48 | PRIM1 | 7964271 | 2.01796686 | 7.35003263 | 10.8029951 | 7.41E-10 | 1.51E-08 | 12.3871181 |
| 49 | FGFR2 | 7936734 | 2.02816874 | 5.68457987 | 14.6751931 | 2.95E-12 | 1.33E-10 | 18.0716049 |
| 50 | RBL1 | 8066136 | 2.02941266 | 7.22785712 | 11.2206488 | 3.82E-10 | 8.52E-09 | 13.0702974 |
| 51 | RPS28 | 8005471 | 2.03219655 | 9.28093336 | 12.2386148 | 8.17E-11 | 2.29E-09 | 14.658919 |
| 52 | TBC1D1 | 8094574 | 2.03242446 | 7.99830463 | 14.6141621 | 3.19E-12 | 1.43E-10 | 17.9921288 |
| 53 | NA | 8005632 | 2.03383359 | 4.39936237 | 13.8934734 | 8.13E-12 | 3.19E-10 | 17.0315558 |
| 54 | NA | 7893334 | 2.03639696 | 7.34207167 | 6.96786958 | 8.58E-07 | 6.63E-06 | 5.12823127 |
| 55 | NA | 7893933 | 2.0385894 | 7.98741452 | 9.8409734 | 3.67E-09 | 5.82E-08 | 10.7387137 |
| 56 | CHMP4C | 8147057 | 2.04150054 | 5.75453935 | 18.4307922 | 3.99E-14 | 3.55E-12 | 22.4743326 |
| 57 | NA | 8097953 | 2.05133219 | 3.21932223 | 9.60033329 | 5.56E-09 | 8.31E-08 | 10.3093097 |
| 58 | DCDC2 | 8124196 | 2.0549051 | 5.55133735 | 8.90383452 | 1.93E-08 | 2.45E-07 | 9.02604666 |
| 59 | ATG4C | 7901895 | 2.06529729 | 6.61414047 | 12.3280455 | 7.16E-11 | 2.07E-09 | 14.7935435 |
| 60 | NCAPG | 8094278 | 2.06930989 | 8.2309742 | 6.36341555 | 3.10E-06 | 2.04E-05 | 3.81130289 |
| 61 | DGKD | 8049317 | 2.07589142 | 6.93815614 | 23.9675343 | 2.45E-16 | 5.01E-14 | 27.6233543 |
| 62 | EXO1 | 7910997 | 2.0768496 | 6.91296181 | 9.17733702 | 1.18E-08 | 1.60E-07 | 9.53723329 |
| 63 | GABRQ | 8170538 | 2.08456036 | 5.49902308 | 20.8463136 | 3.71E-15 | 4.72E-13 | 24.8845789 |
| 64 | NCAM1 | 7943892 | 2.08720083 | 5.66983152 | 16.9504667 | 1.97E-13 | 1.39E-11 | 20.845445 |
| 65 | NA | 7894836 | 2.09187915 | 4.45232856 | 7.87627613 | 1.36E-07 | 1.33E-06 | 7.01880597 |
| 66 | TFAP2A | 8123864 | 2.10199089 | 7.0033406 | 17.2350621 | 1.44E-13 | 1.06E-11 | 21.1685321 |
| 67 | NDC80 | 8019857 | 2.10206257 | 6.24637024 | 9.81751025 | 3.82E-09 | 6.03E-08 | 10.6971541 |
| 68 | NA | 8173164 | 2.10233848 | 6.39551257 | 11.1795211 | 4.07E-10 | 9.01E-09 | 13.0038605 |
| 69 | SLC44A4 | 8179861 | 2.10284238 | 6.49891559 | 18.5239887 | 3.62E-14 | 3.28E-12 | 22.5727629 |
| 70 | SNORA3 | 7938291 | 2.10696166 | 8.25982188 | 16.9323681 | 2.01E-13 | 1.41E-11 | 20.8247313 |
| 71 | TINAGL1 | 7899627 | 2.10780118 | 7.27832446 | 17.9824438 | 6.39E-14 | 5.35E-12 | 21.9941929 |
| 72 | RNF43 | 8016994 | 2.1098366 | 5.64734751 | 14.5891283 | 3.30E-12 | 1.47E-10 | 17.9594467 |
| 73 | MST4 | 8169949 | 2.11013058 | 7.25811421 | 19.2530526 | 1.73E-14 | 1.75E-12 | 23.3270316 |
| 74 | RNF19A | 8152041 | 2.11658585 | 8.47535343 | 20.1732967 | 7.01E-15 | 8.15E-13 | 24.2411573 |
| 75 | NA | 8157139 | 2.12357351 | 9.2342628 | 21.6279154 | 1.82E-15 | 2.62E-13 | 25.6068374 |
| 76 | HSP90AA6P | 8103722 | 2.13002843 | 7.82281933 | 9.21030756 | 1.11E-08 | 1.52E-07 | 9.59821596 |
| 77 | NA | 7895590 | 2.13269738 | 7.17830725 | 7.52629436 | 2.73E-07 | 2.44E-06 | 6.30331965 |
| 78 | SPC25 | 8056572 | 2.13863179 | 6.24274932 | 7.44896559 | 3.19E-07 | 2.78E-06 | 6.14304187 |
| 79 | MCAM | 7952205 | 2.14174252 | 6.6626124 | 19.3931362 | 1.50E-14 | 1.55E-12 | 23.4688484 |
| 80 | HES1 | 8084880 | 2.14755897 | 7.62273605 | 13.1927161 | 2.10E-11 | 7.35E-10 | 16.0565791 |
| 81 | PRPF38A | 7901447 | 2.14984206 | 8.40204239 | 18.8366264 | 2.63E-14 | 2.50E-12 | 22.8995871 |
| 82 | ECT2 | 8083941 | 2.15045971 | 8.46064536 | 11.8948621 | 1.36E-10 | 3.52E-09 | 14.1341849 |
| 83 | PTGR2 | 7975632 | 2.15153623 | 6.67131859 | 17.7257159 | 8.41E-14 | 6.73E-12 | 21.7142145 |
| 84 | SNRPG | 8026339 | 2.15160757 | 7.85054722 | 6.92546157 | 9.37E-07 | 7.16E-06 | 5.037334 |
| 85 | SLC4A4 | 8095585 | 2.15332274 | 7.87280074 | 12.4057857 | 6.40E-11 | 1.91E-09 | 14.909946 |
| 86 | SNORD14C | 7952339 | 2.16252635 | 7.12580895 | 8.20898909 | 7.12E-08 | 7.58E-07 | 7.68392146 |
| 87 | CLU | 8149927 | 2.16298843 | 7.58881558 | 21.3046903 | 2.43E-15 | 3.38E-13 | 25.3113104 |
| 88 | NA | 7895247 | 2.16501475 | 6.1306815 | 13.3143191 | 1.78E-11 | 6.34E-10 | 16.2287891 |
| 89 | TPD52 | 8151475 | 2.16539453 | 4.9995389 | 20.6792948 | 4.34E-15 | 5.42E-13 | 24.7268082 |
| 90 | NA | 7893637 | 2.16866714 | 5.72386616 | 6.74889636 | 1.36E-06 | 9.95E-06 | 4.65641742 |
| 91 | RFC1 | 8099860 | 2.16974301 | 8.27792806 | 15.2513784 | 1.44E-12 | 7.35E-11 | 18.8081604 |
| 92 | PKP2 | 7962212 | 2.17210741 | 6.68547325 | 15.3187555 | 1.33E-12 | 6.87E-11 | 18.892701 |
| 93 | NR2C2AP | 8035628 | 2.17374118 | 6.56625487 | 16.0411636 | 5.59E-13 | 3.28E-11 | 19.7791075 |
| 94 | NA | 7894384 | 2.18386641 | 5.88910298 | 7.43130488 | 3.31E-07 | 2.87E-06 | 6.10632596 |
| 95 | NA | 7896489 | 2.18618552 | 7.75171425 | 8.48084148 | 4.25E-08 | 4.85E-07 | 8.21652166 |
| 96 | NA | 7896671 | 2.18641051 | 3.83926105 | 8.85790264 | 2.10E-08 | 2.63E-07 | 8.9392616 |
| 97 | ATAD5 | 8006187 | 2.18761129 | 5.75179036 | 14.4076008 | 4.16E-12 | 1.78E-10 | 17.7210099 |
| 98 | EGLN3 | 7978544 | 2.18761988 | 6.9427904 | 13.9032446 | 8.03E-12 | 3.16E-10 | 17.0448585 |
| 99 | CFDP1 | 8002865 | 2.18823411 | 7.07015612 | 19.4067852 | 1.48E-14 | 1.54E-12 | 23.4826141 |
| 100 | HLTF | 8091354 | 2.18835147 | 8.0353112 | 11.424569 | 2.78E-10 | 6.48E-09 | 13.3970542 |
| 101 | VAMP8 | 8043197 | 2.19167894 | 6.54864394 | 16.647067 | 2.77E-13 | 1.81E-11 | 20.495519 |
| 102 | CNOT10 | 8078412 | 2.19833816 | 7.51326494 | 11.4206335 | 2.80E-10 | 6.51E-09 | 13.3907895 |
| 103 | MTF2 | 7903032 | 2.20437232 | 7.58706887 | 14.1396823 | 5.88E-12 | 2.41E-10 | 17.3643737 |
| 104 | GINS4 | 8146130 | 2.20464185 | 6.6553818 | 12.2376024 | 8.18E-11 | 2.29E-09 | 14.6573906 |
| 105 | RAD21L1 | 8060395 | 2.20969824 | 4.40014143 | 10.4827553 | 1.25E-09 | 2.31E-08 | 11.8502234 |
| 106 | CDSN | 8178442 | 2.21349898 | 8.23912874 | 6.41104431 | 2.80E-06 | 1.86E-05 | 3.91669907 |
| 107 | VLDLR | 8154100 | 2.2187117 | 7.06027521 | 11.4013092 | 2.88E-10 | 6.69E-09 | 13.3600048 |
| 108 | DAPK1 | 8156199 | 2.22464272 | 6.98728409 | 13.4727036 | 1.43E-11 | 5.25E-10 | 16.4511592 |
| 109 | MLLT11 | 7905329 | 2.22538339 | 7.47016403 | 16.1588008 | 4.87E-13 | 2.91E-11 | 19.9200795 |
| 110 | NIPAL1 | 8094938 | 2.22548886 | 5.75442288 | 15.6461155 | 8.93E-13 | 4.91E-11 | 19.2988599 |
| 111 | NA | 7896175 | 2.22990215 | 9.62099474 | 10.0363225 | 2.63E-09 | 4.39E-08 | 11.0821702 |
| 112 | NA | 7892719 | 2.2379985 | 7.46274651 | 14.206168 | 5.39E-12 | 2.23E-10 | 17.4534073 |
| 113 | NA | 7896243 | 2.24006728 | 11.5347677 | 19.7091486 | 1.10E-14 | 1.20E-12 | 23.7852112 |
| 114 | NA | 7894089 | 2.24786942 | 6.6373161 | 8.74256068 | 2.60E-08 | 3.18E-07 | 8.72013394 |
| 115 | NA | 7925432 | 2.25318279 | 3.81672019 | 13.5691831 | 1.26E-11 | 4.67E-10 | 16.5855589 |
| 116 | TRIM24 | 8136473 | 2.25472646 | 7.61377049 | 19.8069911 | 9.99E-15 | 1.13E-12 | 23.8821775 |
| 117 | NA | 7895981 | 2.26064699 | 7.42894572 | 8.3961376 | 4.98E-08 | 5.57E-07 | 8.05161548 |
| 118 | MALT1 | 8021418 | 2.26066974 | 7.91999315 | 11.6248785 | 2.04E-10 | 4.98E-09 | 13.7137873 |
| 119 | VRK1 | 7976621 | 2.26515612 | 6.89382508 | 12.4731797 | 5.80E-11 | 1.76E-09 | 15.0103909 |
| 120 | NA | 7896553 | 2.27140394 | 6.24902763 | 8.41997113 | 4.76E-08 | 5.36E-07 | 8.09811099 |
| 121 | NEO1 | 7984704 | 2.27170483 | 8.1910722 | 19.700759 | 1.11E-14 | 1.20E-12 | 23.7768752 |
| 122 | CMIP | 7997427 | 2.27256598 | 7.72459866 | 17.2978417 | 1.34E-13 | 1.00E-11 | 21.2391445 |
| 123 | PPP4R1L | 8067248 | 2.27266055 | 6.28166237 | 18.6163754 | 3.29E-14 | 3.01E-12 | 22.6698795 |
| 124 | EZH2 | 8143663 | 2.27447549 | 7.39310191 | 12.6015278 | 4.82E-11 | 1.51E-09 | 15.200497 |
| 125 | WDHD1 | 7979281 | 2.27465729 | 7.18969177 | 14.7769687 | 2.60E-12 | 1.20E-10 | 18.2035088 |
| 126 | MYO5B | 8023267 | 2.27467443 | 5.59508532 | 15.025516 | 1.90E-12 | 9.23E-11 | 18.5223614 |
| 127 | NA | 7892903 | 2.28503647 | 6.65757649 | 13.010088 | 2.71E-11 | 9.15E-10 | 15.7954938 |
| 128 | STAT4 | 8057771 | 2.30175379 | 6.07447466 | 14.2826738 | 4.88E-12 | 2.05E-10 | 17.5554228 |
| 129 | NA | 8023526 | 2.31468466 | 8.50641431 | 17.6505107 | 9.12E-14 | 7.24E-12 | 21.6314861 |
| 130 | NA | 8153039 | 2.32825212 | 6.99122955 | 10.0817764 | 2.43E-09 | 4.09E-08 | 11.161435 |
| 131 | B4GALNT1 | 7964484 | 2.33068802 | 6.8222726 | 18.5254406 | 3.62E-14 | 3.28E-12 | 22.5742926 |
| 132 | SLC17A7 | 8038367 | 2.33442432 | 6.94958534 | 20.5459405 | 4.92E-15 | 5.96E-13 | 24.5999448 |
| 133 | DSG2 | 8020779 | 2.33536942 | 4.97399631 | 16.3497591 | 3.90E-13 | 2.43E-11 | 20.1469706 |
| 134 | NA | 7893782 | 2.33719479 | 9.10876637 | 8.69701798 | 2.83E-08 | 3.42E-07 | 8.63313828 |
| 135 | CDH1 | 7996837 | 2.34200541 | 5.57277478 | 15.2892755 | 1.38E-12 | 7.08E-11 | 18.8557515 |
| 136 | NA | 7893766 | 2.34407302 | 7.86504898 | 13.7413396 | 9.96E-12 | 3.78E-10 | 16.8234203 |
| 137 | HMGB2 | 8103728 | 2.34420762 | 7.74269028 | 19.7664074 | 1.04E-14 | 1.16E-12 | 23.8420132 |
| 138 | POLA1 | 8166525 | 2.35686848 | 7.25314025 | 11.8514179 | 1.45E-10 | 3.71E-09 | 14.0670344 |
| 139 | SLC24A2 | 8160321 | 2.3592179 | 5.81201524 | 16.6852303 | 2.66E-13 | 1.77E-11 | 20.5398514 |
| 140 | NA | 7952750 | 2.36354619 | 4.23346877 | 6.36324894 | 3.10E-06 | 2.04E-05 | 3.81093372 |
| 141 | HMGN1 | 8176191 | 2.38455222 | 10.2078731 | 26.9771666 | 2.39E-17 | 7.43E-15 | 29.9409845 |
| 142 | HABP2 | 7930561 | 2.38817162 | 6.55986288 | 15.4062563 | 1.19E-12 | 6.28E-11 | 19.0020064 |
| 143 | GREB1L | 8020384 | 2.38879267 | 6.48137167 | 16.5424837 | 3.12E-13 | 2.01E-11 | 20.3735563 |
| 144 | CRABP1 | 7985159 | 2.3948198 | 6.8501425 | 14.7957838 | 2.54E-12 | 1.17E-10 | 18.2278078 |
| 145 | CLSTN2 | 8083034 | 2.40076952 | 6.80825528 | 21.5782665 | 1.90E-15 | 2.72E-13 | 25.5617269 |
| 146 | ERMP1 | 8159992 | 2.41524299 | 7.56251825 | 18.4252645 | 4.01E-14 | 3.56E-12 | 22.4684797 |
| 147 | NA | 7953965 | 2.42214693 | 5.82706107 | 8.9014529 | 1.94E-08 | 2.46E-07 | 9.0215534 |
| 148 | INTS7 | 7924119 | 2.42350734 | 7.4145726 | 10.5962377 | 1.04E-09 | 1.98E-08 | 12.0418024 |
| 149 | TOPBP1 | 8090772 | 2.43402211 | 8.07309577 | 14.056508 | 6.56E-12 | 2.65E-10 | 17.2524915 |
| 150 | EEF1A2 | 8067652 | 2.44376126 | 8.1574881 | 19.731789 | 1.07E-14 | 1.19E-12 | 23.8076898 |
| 151 | ENY2 | 8147883 | 2.45203403 | 8.76405521 | 12.212737 | 8.48E-11 | 2.36E-09 | 14.6198197 |
| 152 | HNRNPA1 | 7916562 | 2.45344834 | 10.9786515 | 10.5154622 | 1.18E-09 | 2.21E-08 | 11.9055885 |
| 153 | PIK3C2B | 7923662 | 2.45857049 | 6.32270469 | 14.3011537 | 4.77E-12 | 2.00E-10 | 17.579995 |
| 154 | ATAD2 | 8152668 | 2.45964897 | 7.94847628 | 12.9638343 | 2.89E-11 | 9.66E-10 | 15.7288966 |
| 155 | NA | 8127991 | 2.46836839 | 5.89868904 | 20.5580068 | 4.86E-15 | 5.92E-13 | 24.6114567 |
| 156 | EPHX2 | 8145532 | 2.46910238 | 5.79774447 | 22.3076519 | 9.95E-16 | 1.55E-13 | 26.2143413 |
| 157 | EZR | 8130505 | 2.47494856 | 9.30867191 | 13.784553 | 9.40E-12 | 3.59E-10 | 16.8827362 |
| 158 | DTL | 7909568 | 2.49227512 | 7.84065685 | 8.36473287 | 5.29E-08 | 5.86E-07 | 7.99023584 |
| 159 | HBEGF | 8114572 | 2.49595974 | 6.91649862 | 11.5272757 | 2.37E-10 | 5.67E-09 | 13.5599755 |
| 160 | PDZK1 | 7904843 | 2.5032381 | 4.7031964 | 11.4731409 | 2.58E-10 | 6.07E-09 | 13.4742398 |
| 161 | SNX10 | 8131957 | 2.50390595 | 6.23501202 | 18.8203416 | 2.67E-14 | 2.53E-12 | 22.8826902 |
| 162 | NA | 7894331 | 2.51366392 | 7.80978219 | 13.9904877 | 7.16E-12 | 2.86E-10 | 17.1632856 |
| 163 | AK4 | 7962183 | 2.51957393 | 8.86175883 | 13.4357867 | 1.50E-11 | 5.49E-10 | 16.3995217 |
| 164 | CEP85 | 7899134 | 2.53780497 | 6.75753244 | 22.8608931 | 6.17E-16 | 1.04E-13 | 26.6953477 |
| 165 | STRBP | 8164013 | 2.54017924 | 5.53757968 | 17.2762168 | 1.37E-13 | 1.02E-11 | 21.2148481 |
| 166 | CTDSPL2 | 7983335 | 2.5487624 | 7.95489599 | 14.3290868 | 4.60E-12 | 1.94E-10 | 17.6170853 |
| 167 | ENO3 | 8004043 | 2.55522182 | 6.31982927 | 17.6190101 | 9.44E-14 | 7.44E-12 | 21.5967375 |
| 168 | ANK2 | 8096959 | 2.55911681 | 6.20062929 | 12.4473349 | 6.02E-11 | 1.81E-09 | 14.9719224 |
| 169 | NA | 8013356 | 2.57070156 | 4.15160584 | 11.7485962 | 1.69E-10 | 4.24E-09 | 13.9073485 |
| 170 | HKDC1 | 7927998 | 2.57094428 | 6.16982698 | 10.2961124 | 1.70E-09 | 3.01E-08 | 11.5319346 |
| 171 | PRKAA2 | 7901720 | 2.57684183 | 7.59017507 | 17.9373652 | 6.70E-14 | 5.52E-12 | 21.9453016 |
| 172 | MAPRE3 | 8040742 | 2.58209009 | 7.72963603 | 15.4029862 | 1.20E-12 | 6.29E-11 | 18.9979312 |
| 173 | PLCE1 | 7929388 | 2.58464927 | 5.67916875 | 12.341484 | 7.03E-11 | 2.05E-09 | 14.8137067 |
| 174 | COL11A1 | 7918064 | 2.60137198 | 7.72058994 | 13.960777 | 7.44E-12 | 2.96E-10 | 17.1230251 |
| 175 | H19 | 7945680 | 2.60462938 | 7.62491688 | 12.4985812 | 5.59E-11 | 1.71E-09 | 15.0481381 |
| 176 | NA | 8150204 | 2.60945942 | 6.53416203 | 16.8720939 | 2.15E-13 | 1.50E-11 | 20.7556026 |
| 177 | NA | 7892832 | 2.62400357 | 6.77544106 | 8.48190415 | 4.24E-08 | 4.84E-07 | 8.21858455 |
| 178 | NA | 7894877 | 2.64061387 | 8.64860253 | 9.186077 | 1.16E-08 | 1.58E-07 | 9.55341219 |
| 179 | ALDOC | 8013660 | 2.64089905 | 8.05324971 | 10.0865332 | 2.41E-09 | 4.06E-08 | 11.169716 |
| 180 | KRT7 | 7955613 | 2.65165063 | 7.57247399 | 14.1912709 | 5.50E-12 | 2.26E-10 | 17.4334888 |
| 181 | PGM2 | 8094556 | 2.65364239 | 7.71450119 | 16.3306955 | 3.99E-13 | 2.48E-11 | 20.124427 |
| 182 | NA | 7896650 | 2.6550286 | 4.49542607 | 6.97205584 | 8.50E-07 | 6.58E-06 | 5.13719155 |
| 183 | SEMA7A | 7990345 | 2.68293503 | 7.30966957 | 18.2906765 | 4.62E-14 | 4.07E-12 | 22.3254659 |
| 184 | NA | 7895511 | 2.68593511 | 4.34317136 | 5.88541011 | 8.87E-06 | 5.11E-05 | 2.73919201 |
| 185 | NA | 7893073 | 2.68669906 | 9.81756213 | 20.1341687 | 7.28E-15 | 8.37E-13 | 24.2031129 |
| 186 | CPA4 | 8136200 | 2.69306364 | 6.44254587 | 11.8927461 | 1.36E-10 | 3.53E-09 | 14.1309187 |
| 187 | NA | 8147990 | 2.69968237 | 6.41600542 | 10.9854657 | 5.53E-10 | 1.18E-08 | 12.6879312 |
| 188 | CHD7 | 8146579 | 2.7023417 | 6.37758843 | 18.9875014 | 2.25E-14 | 2.21E-12 | 23.05548 |
| 189 | NA | 7981512 | 2.70307368 | 8.19740841 | 18.7521283 | 2.86E-14 | 2.68E-12 | 22.8117621 |
| 190 | HOXA9 | 8138749 | 2.72430247 | 6.61352671 | 15.8540693 | 6.97E-13 | 3.96E-11 | 19.5529888 |
| 191 | TUFT1 | 7905428 | 2.72440467 | 7.1760908 | 15.9702157 | 6.07E-13 | 3.52E-11 | 19.6936392 |
| 192 | NA | 7992893 | 2.72456837 | 6.93000483 | 6.83687504 | 1.13E-06 | 8.45E-06 | 4.84671512 |
| 193 | CKS1B | 8112327 | 2.72857123 | 10.0741405 | 16.2950555 | 4.15E-13 | 2.56E-11 | 20.0822176 |
| 194 | ZNF711 | 8168589 | 2.73330023 | 5.32778909 | 22.9922757 | 5.51E-16 | 9.48E-14 | 26.8078637 |
| 195 | EPCAM | 8041853 | 2.74330616 | 5.57020937 | 16.4168206 | 3.61E-13 | 2.27E-11 | 20.2260865 |
| 196 | NA | 8055198 | 2.76402264 | 3.55326383 | 16.6928346 | 2.63E-13 | 1.75E-11 | 20.548674 |
| 197 | SUMO1P3 | 7906574 | 2.79192939 | 4.99827761 | 12.2180591 | 8.42E-11 | 2.34E-09 | 14.6278663 |
| 198 | FAM49B | 8152845 | 2.80471354 | 7.76935604 | 18.8147946 | 2.69E-14 | 2.54E-12 | 22.8769315 |
| 199 | ELOVL7 | 8112274 | 2.80560934 | 6.03950173 | 28.097254 | 1.07E-17 | 3.85E-15 | 30.73524 |
| 200 | FNBP1L | 7903092 | 2.80588058 | 6.24089538 | 16.828627 | 2.26E-13 | 1.56E-11 | 20.7056105 |
| 201 | NA | 7892847 | 2.81232289 | 7.76264369 | 15.0342726 | 1.88E-12 | 9.15E-11 | 18.5335113 |
| 202 | SERPINA1 | 7981068 | 2.83394064 | 6.90907404 | 16.0392504 | 5.60E-13 | 3.28E-11 | 19.7768071 |
| 203 | NA | 7894440 | 2.85451374 | 5.61186968 | 8.40352642 | 4.91E-08 | 5.50E-07 | 8.06603788 |
| 204 | MAD2L1 | 8102560 | 2.8636118 | 6.70687361 | 13.3829965 | 1.62E-11 | 5.84E-10 | 16.3254777 |
| 205 | CXADR | 8067955 | 2.88160919 | 6.92584053 | 15.7640969 | 7.75E-13 | 4.35E-11 | 19.4434034 |
| 206 | MYBL2 | 8062766 | 2.88260334 | 7.70486674 | 18.4607531 | 3.87E-14 | 3.45E-12 | 22.5060269 |
| 207 | ABCB1 | 8140782 | 2.90098817 | 6.38354345 | 13.8802746 | 8.28E-12 | 3.24E-10 | 17.0135743 |
| 208 | TES | 8135576 | 2.9024494 | 8.22514705 | 12.1973516 | 8.68E-11 | 2.40E-09 | 14.5965429 |
| 209 | HOOK1 | 7901765 | 2.9896499 | 4.77183361 | 18.7895759 | 2.76E-14 | 2.60E-12 | 22.8507301 |
| 210 | FZD3 | 8145611 | 3.02338498 | 5.7968414 | 28.7627965 | 6.74E-18 | 2.50E-15 | 31.1913953 |
| 211 | FKBP5 | 8125919 | 3.02538123 | 6.4677039 | 14.0922541 | 6.26E-12 | 2.55E-10 | 17.300644 |
| 212 | NRCAM | 8142270 | 3.02642838 | 5.57622351 | 19.1004738 | 2.01E-14 | 2.01E-12 | 23.1714432 |
| 213 | SLCO2A1 | 8090823 | 3.03452623 | 6.9133516 | 16.2315966 | 4.47E-13 | 2.72E-11 | 20.0068557 |
| 214 | ANAPC1 | 8043322 | 3.03562364 | 6.88322171 | 12.8114003 | 3.58E-11 | 1.17E-09 | 15.5080484 |
| 215 | LIPH | 8092541 | 3.03622456 | 5.39065043 | 17.2373845 | 1.43E-13 | 1.06E-11 | 21.1711484 |
| 216 | PELI1 | 8052654 | 3.03737259 | 7.06621251 | 28.2147596 | 9.86E-18 | 3.58E-15 | 30.8166099 |
| 217 | RNU2-1 | 8019709 | 3.04178266 | 8.36151966 | 8.71218139 | 2.75E-08 | 3.34E-07 | 8.66213326 |
| 218 | NPNT | 8096704 | 3.10837452 | 6.02794409 | 17.1439472 | 1.59E-13 | 1.16E-11 | 21.0656283 |
| 219 | IQGAP2 | 8106354 | 3.11634208 | 5.50294369 | 16.1925889 | 4.68E-13 | 2.80E-11 | 19.9604 |
| 220 | PCOLCE2 | 8091243 | 3.12111239 | 5.8795057 | 25.1797326 | 9.29E-17 | 2.26E-14 | 28.5911471 |
| 221 | NA | 8159963 | 3.13449381 | 6.96267564 | 13.6570157 | 1.12E-11 | 4.19E-10 | 16.7072254 |
| 222 | MET | 8135601 | 3.17651123 | 7.69522789 | 13.2464207 | 1.95E-11 | 6.87E-10 | 16.1327935 |
| 223 | TSPAN12 | 8142524 | 3.18283607 | 6.98319106 | 14.7602829 | 2.65E-12 | 1.22E-10 | 18.1819373 |
| 224 | ZNF669 | 7925677 | 3.20341339 | 7.22552375 | 33.1913705 | 3.93E-19 | 2.76E-16 | 33.9620656 |
| 225 | NA | 8083455 | 3.21504656 | 4.87307273 | 19.0352053 | 2.15E-14 | 2.12E-12 | 23.1045264 |
| 226 | BAMBI | 7926875 | 3.22058022 | 7.37824868 | 19.7844626 | 1.02E-14 | 1.14E-12 | 23.8598916 |
| 227 | HOXA5 | 8138735 | 3.2904172 | 7.01321255 | 15.2333935 | 1.47E-12 | 7.45E-11 | 18.7855388 |
| 228 | SNORD14E | 7952335 | 3.34098593 | 5.56133484 | 10.7500204 | 8.07E-10 | 1.62E-08 | 12.2990974 |
| 229 | NA | 8167878 | 3.41034894 | 5.03351189 | 18.9395955 | 2.37E-14 | 2.30E-12 | 23.0061083 |
| 230 | DEPTOR | 8148059 | 3.43483841 | 6.80199931 | 12.5142933 | 5.47E-11 | 1.68E-09 | 15.0714562 |
| 231 | CDK1 | 7927710 | 3.44954002 | 6.88381778 | 12.135184 | 9.51E-11 | 2.59E-09 | 14.5022537 |
| 232 | CPE | 8098204 | 3.45495176 | 7.84432506 | 13.990523 | 7.15E-12 | 2.86E-10 | 17.1633334 |
| 233 | KRT18 | 8154725 | 3.4657099 | 7.79777234 | 13.3661062 | 1.65E-11 | 5.95E-10 | 16.3017364 |
| 234 | ANXA8L2 | 7927307 | 3.47571089 | 9.38151072 | 20.8573703 | 3.68E-15 | 4.72E-13 | 24.8949798 |
| 235 | SEL1L3 | 8099721 | 3.48916359 | 5.75938785 | 22.0837707 | 1.21E-15 | 1.85E-13 | 26.0163025 |
| 236 | FAM25B | 7933423 | 3.50209042 | 8.2344305 | 19.5151953 | 1.33E-14 | 1.40E-12 | 23.5916232 |
| 237 | AGPAT9 | 8096116 | 3.56766896 | 6.06119466 | 15.5842698 | 9.62E-13 | 5.25E-11 | 19.2227048 |
| 238 | GABRA1 | 8109663 | 3.59157484 | 5.90762193 | 14.5423122 | 3.50E-12 | 1.54E-10 | 17.8981978 |
| 239 | ANKRD1 | 7934979 | 3.61699369 | 6.04150967 | 14.7967664 | 2.53E-12 | 1.17E-10 | 18.2290761 |
| 240 | ATP1B1 | 7907160 | 3.69229025 | 7.43813179 | 19.1954688 | 1.83E-14 | 1.84E-12 | 23.2684501 |
| 241 | LIMCH1 | 8094789 | 3.7225156 | 6.27857279 | 29.8591579 | 3.21E-18 | 1.44E-15 | 31.9186691 |
| 242 | GPR89B | 7904930 | 3.73208404 | 7.0480906 | 11.2416666 | 3.69E-10 | 8.30E-09 | 13.1041794 |
| 243 | NA | 7893789 | 3.74236574 | 9.72611928 | 26.6811275 | 2.97E-17 | 8.81E-15 | 29.7252434 |
| 244 | ANXA8L1 | 7933312 | 3.87936408 | 7.38843001 | 19.6937366 | 1.12E-14 | 1.21E-12 | 23.7698951 |
| 245 | PLS1 | 8083146 | 3.97494677 | 5.93749144 | 39.3709906 | 1.30E-20 | 2.22E-17 | 37.2016298 |
| 246 | FAM60A | 8180256 | 4.11881633 | 7.25351082 | 24.3972475 | 1.73E-16 | 3.80E-14 | 27.9720446 |
| 247 | CHRDL1 | 8174513 | 4.14131956 | 7.99146333 | 25.7414969 | 6.02E-17 | 1.62E-14 | 29.0235127 |
| 248 | CDH2 | 8022674 | 4.15258618 | 8.14501061 | 19.4786733 | 1.38E-14 | 1.45E-12 | 23.5549642 |
| 249 | NA | 8058662 | 4.18405389 | 4.46638561 | 23.6495556 | 3.18E-16 | 6.19E-14 | 27.3612127 |
| 250 | PMEL | 7963970 | 4.2516152 | 6.55368383 | 24.8717991 | 1.18E-16 | 2.81E-14 | 28.3498995 |
| 251 | GPC4 | 8175217 | 4.38124401 | 7.34451059 | 28.0645606 | 1.10E-17 | 3.85E-15 | 30.7125361 |
| 252 | CCDC88C | 7980828 | 4.39396247 | 5.17508211 | 21.1426135 | 2.82E-15 | 3.79E-13 | 25.1614594 |
| 253 | NA | 7973743 | 4.53730473 | 7.41731202 | 20.4614052 | 5.33E-15 | 6.43E-13 | 24.5191091 |
| 254 | GPR126 | 8122365 | 4.99044584 | 7.33454948 | 28.638736 | 7.34E-18 | 2.70E-15 | 31.1072269 |
| 255 | NA | 8156056 | 5.05442468 | 5.60858417 | 24.4403268 | 1.67E-16 | 3.69E-14 | 28.0066551 |
| 256 | RBP1 | 8091078 | 5.06056682 | 7.61680006 | 26.21774 | 4.20E-17 | 1.15E-14 | 29.3824578 |
| 257 | TSPAN7 | 8166784 | 5.17315788 | 6.96853771 | 35.0931152 | 1.30E-19 | 1.23E-16 | 35.0282412 |
| 258 | EPCAM | 8098439 | 5.69643312 | 6.39864001 | 29.3687549 | 4.46E-18 | 1.87E-15 | 31.59698 |
| 259 | CDH17 | 8151795 | 5.74087667 | 6.57163961 | 43.1727062 | 2.06E-21 | 6.05E-18 | 38.9103634 |
| 260 | NA | 7951339 | 5.8696436 | 7.0937068 | 27.312275 | 1.87E-17 | 6.05E-15 | 30.1822121 |
|  |  |  |  |  |  |  |  |  |
