# Supplementary material for: CEMP1 Induces Transformation in Human Gingival Fibroblasts
Source: PLoS One. 2015 May 26;10(5):e0127286. doi: 10.1371/journal.pone.0127286 (PMC4444236; doi:10.1371/journal.pone.0127286)
Supplement: S2 Table — CEMP1 overexpression in human cancer samples. (DOCX) [file pone.0127286.s005.docx]

Table S2.

| **CANCER TYPE** | **STUDY NAME** | **TOTAL SAMPLES** | **EXPRESSING CEMP1** | **MICROARRAY DATABASE ID** |
| --- | --- | --- | --- | --- |
| **Oral cancer** | Cigarette smoke effect on the oral mucosa | 79 | 6 | ID: 65498196 |
| **Benzene exposure** | Occupational benzene exposure: peripheral blood mononuclear cells (HG-U133B) | 16 | 3 | ID: 60916232 |
|  | 1,2,4-benzenetriol effect on peripheral blood mononuclear cells in vitro | 16 | 4 | ID: 40532669 |
| **Cervical cancer** | Preinvasive and invasive cervical squamous cell carcinomas | 38 | 5 | ID: 53614669 |
|  | Cervical cancer tumorigenesis | 61 | 14 | ID: 52157069 |
|  | Different areas of cervical cancer tumors | 33 | 9 | ID: 32027590 |
| **Breast cancer** | Inflammatory breast cancer: tumor | 48 | 14 | ID: 48506869 |
|  | Inflammatory breast cancer: stroma | 47 | 6 | ID: 48483869 |
| **Leukemia** | Imatinib effect on K562 leukemia cell line (V) | 6 | 12 | ID: 47255869 |
|  | Imatinib effect on K562 leukemia cell line (IV) | 6 | 3 | ID: 47232869 |
|  | Imatinib effect on K562 leukemia cell line (III) | 6 | 3 | ID: 47209869 |
|  | Imatinib effect on K562 leukemia cell line (II) | 6 | 3 | ID: 47186869 |
|  | Rapamycin effect on a glucorticoid-resistant T cell lymphoblastic leukemia cell line: time course | 9 | 4 | ID: 33847969 |
| **Ovarian endometriosis** | Ovarian endometriosis | 20 | 6 | ID: 41964196 |
| **Prostate cancer** | Prostate cancer | 16 | 12 | ID: 39391416 |
|  | Metastatic prostate cancer (HG-U95C) | 164 | 10 | ID: 34888299 |
| **Lung cancer** | Gefitinib effect on various non-small cell lung cancer cell lines (HG-U133A) | 45 | 24 | ID: 29741469 |
|  | Asbestos effect on epithelial and mesothelial lung cell lines: time course | 27 | 9 | ID: 35698396 |
